# Tethered Indoxyl-Glucuronides for Enzymatically Triggered Cross-Linking

**DOI:** 10.3390/molecules28104143

**Published:** 2023-05-17

**Authors:** Juno Son, Zhiyuan Wu, Jinghuai Dou, Hikaru Fujita, Phuong-Lien Doan Cao, Qihui Liu, Jonathan S. Lindsey

**Affiliations:** Department of Chemistry, North Carolina State University, Raleigh, NC 27695, USA; json3@ncsu.edu (J.S.); zwu12@ncsu.edu (Z.W.); jdou@ncsu.edu (J.D.); 45kaku@gmail.com (H.F.); ldcao@ncsu.edu (P.-L.D.C.); qliu26@ncsu.edu (Q.L.)

**Keywords:** chromogenic, dimerization, β-glucuronidase, indigoid, indole, oxidation

## Abstract

Indoxyl-glucuronides, upon treatment with β-glucuronidase under physiological conditions, are well known to afford the corresponding indigoid dye via oxidative dimerization. Here, seven indoxyl-glucuronide target compounds have been prepared along with 22 intermediates. Of the target compounds, four contain a conjugatable handle (azido-PEG, hydroxy-PEG, or BCN) attached to the indoxyl moiety, while three are isomers that include a PEG-ethynyl group at the 5-, 6-, or 7-position. All seven target compounds have been examined in indigoid-forming reactions upon treatment with β-glucuronidase from two different sources and rat liver tritosomes. Taken together, the results suggest the utility of tethered indoxyl-glucuronides for use in bioconjugation chemistry with a chromogenic readout under physiological conditions.

## 1. Introduction

**Scheme 1:**
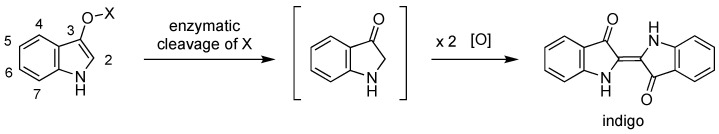
Indigo formation upon enzymatic cleavage and oxidative dimerization.

The ability to cross-link molecules under physiological conditions may underpin a range of applications in bioorganic chemistry [1,2,3,4,5,6,7,8,9]. One class of molecules that has served well in this regard contains the indoxyl motif, the spontaneous oxidative dimerization of which affords the indigoid dye. The formation of the indoxyl species can be triggered by enzymatic action from the corresponding indoxyl-X compound, where X = phosphate, sulfate, glucoside, or glucuronide (Scheme 1). A comprehensive review of such compounds has been reported by Kiernan [10]. We have been interested in extending the core indoxyl chemistry beyond its chief present use in histology (to identify the location of enzymes in cells and tissues) and enabling applications for bioconjugation chemistry. Such applications require the presence of a tether attached to the indoxyl moiety. 

**Chart 1:**
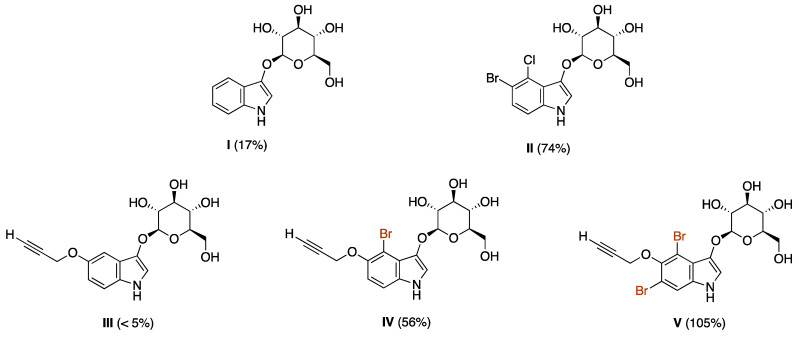
Yields of indigoid dye from various indoxyl-glucosides.

The yields of indigoid formation can range considerably depending on the nature of substituents in the indoxyl moiety (Chart 1). The unsubstituted indoxyl-glucoside (**I**) affords indigo in 17% yield upon treatment with a β-glucosidase (pH 5), whereas the 4-chloro-5-bromo derivative (**II**) affords the corresponding indigoid in 74% yield [5]. We prepared indoxyl-glucosides that bear an alkoxy tether at the indoxyl 5-position [5,6]. For the propargyloxy substituent, the yield was <5% (**III**), but increased upon inclusion of flanking bromine atoms, affording 56% with one bromine (**IV**) or an essentially quantitative yield with two bromines (**V**). Nearly identical results were obtained with the methoxycarbonyl substituent [5], and similar results have been observed with various derivatized PEG groups [9].

In the work leading to the present paper, we sought to pursue two lines of inquiry. In one, we switched from an indoxyl-glucoside to an indoxyl-glucuronide with the objective of increasing the water solubility afforded by the carboxylic acid/carboxylate moiety of the latter. The requisite β-glucuronidase enzymes are known to be prevalent in various tissues, including cancerous tumors [11,12,13,14,15,16,17]. Two, we sought to investigate the utility of tethers positioned at the 5-, 6-, or 7-positions. The thinking behind the latter study was double-pronged: (i) perhaps a substituent positioned more distal to the putative site of enzymatic action (for cleavage of the glycoside) might enable higher yields, and (ii) given that a chief rationale for use of the 5-alkoxy substituent was the convenience of 5-benzyloxyindole-3-carbaldehyde as a commercially available starting material, perhaps starting afresh with the 5-, 6-, or 7-bromoindoxyl-3-carbaldehyde for substitution with an ethyne might enable simplification of the synthesis without requiring flanking halogen substituents.

Here we report the synthesis of seven indoxyl-glucuronide target compounds. Four are equipped with 5-alkoxy substituents and flanking bromine atoms (4,6-dibromo substitution). Three are positional isomers with a PEG-ethyne linker at the 5-, 6-, or 7-positions. Each indoxyl-glucuronide has been examined upon treatment with β-glucuronidase to form the corresponding indigoid dye. Characterization of the yields by absorption spectroscopy has enabled evaluation of the effects of the various substituents.

## 2. Results and Discussion

### 2.1. Synthesis of Indoxyl-Glucuronide Scaffolds

**Scheme 2:**
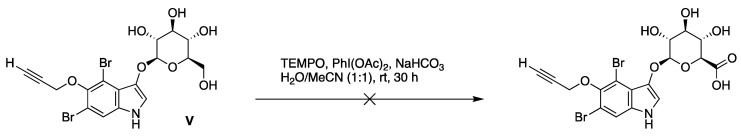
Attempted selective oxidation of an indoxyl-glucoside.

We previously prepared 25 indoxyl-glucosides [5,6,9], and here we considered whether indoxyl β-glucosides could be converted directly to the corresponding indoxyl β-glucuronides. The primary hydroxy group (6-position) of a methyl α-glucoside or phenyl β-glucoside was reported to undergo selective oxidation to give the corresponding glucuronide upon treatment with 2,2,6,6-tetramethyl-1-piperidinyloxy free radical (TEMPO) and a co-oxidant such as PhI(OAc)_2_ or *t*-BuOCl [18,19]. On the other hand, oxidation of indoxyl β-glucoside **V** with TEMPO/PhI(OAc)_2_ was unsuccessful (Scheme 2), which we attributed to the presence of the free N–H moiety of the electron-rich indole.

**Scheme 3:**
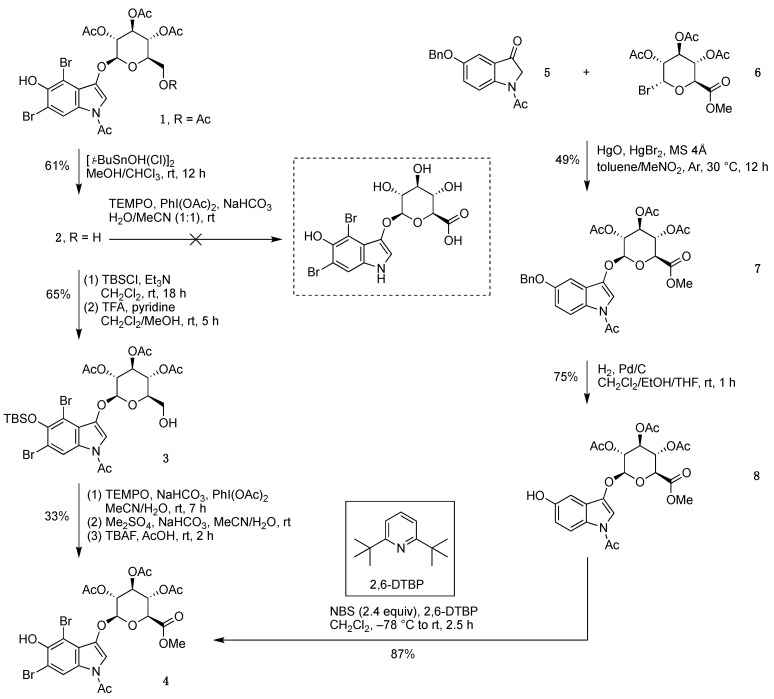
Two routes to indoxyl-glucuronide methyl ester **4**.

We decided to apply the same reaction conditions to a fully protected substrate. Thus, indoxyl-glucoside **1** [6] was subjected to selective deacetylation of the 6-*O*-acetyl group by treatment with [*t*-BuSnOH(Cl)]_2_ in methanol [20,21], which afforded **2** in 61% yield (Scheme 3). Attempted oxidation of **2** by treatment with TEMPO/PhI(OAc)_2_ was unsuccessful as well, which could stem from the presence of the free 5-OH group of the indoxyl moiety. The 5-OH was then protected with a *tert*-butyldimethylsilyl (TBS) group in the presence of triethylamine to give **3** in 65% yield. Treatment of **3**—wherein both indole functional groups are protected—with TEMPO/PhI(OAc)_2_ resulted in oxidation of the primary hydroxyl group of the glucoside moiety; methylation of the resulting carboxylate with dimethyl sulfate and cleavage of the TBS group with tetra-*n*-butylammonium fluoride (TBAF) gave the indoxyl-glucuronide methyl ester **4** in 33% yield.

In an alternate route (developed to prepare indoxyl-glucosides) [7], a solution of 5-benzyloxyindolinone **5** [6] in toluene/nitromethane was treated with acetobromo-α-D-glucuronic acid methyl ester (**6**) in the presence of mercury(II) oxide, mercury(II) bromide, and molecular sieves 4 Å at 30 °C to give indoxyl-glucuronide methyl ester **7** in 49% yield (Scheme 3). Debenzylation in CH_2_Cl_2_/ethanol/tetrahydrofuran (THF) containing Pd/C with H_2_ (1 atm) gave the 5-hydroxy product **8**. While ^1^H NMR spectroscopy showed tiny peaks in the 5.55–5.60 ppm region consistent with the α-anomer [6] of **8**, such peaks corresponded at most to the 2–3% level and were not observed following recrystallization in hexanes/CH_2_Cl_2_. In this manner, indoxyl-glucuronide **8** was obtained in 75% yield. Treatment of **8** with 2.4 equiv of *N*-bromosuccinimide (NBS) in CH_2_Cl_2_ containing the acid scavenger 2,6-di-*tert*-butylpyridine (2,6-DTBP) [22] at −78 °C gave **4** in 87% yield.

### 2.2. Introduction of Linkers and Handles for Conjugation

**Scheme 4:**
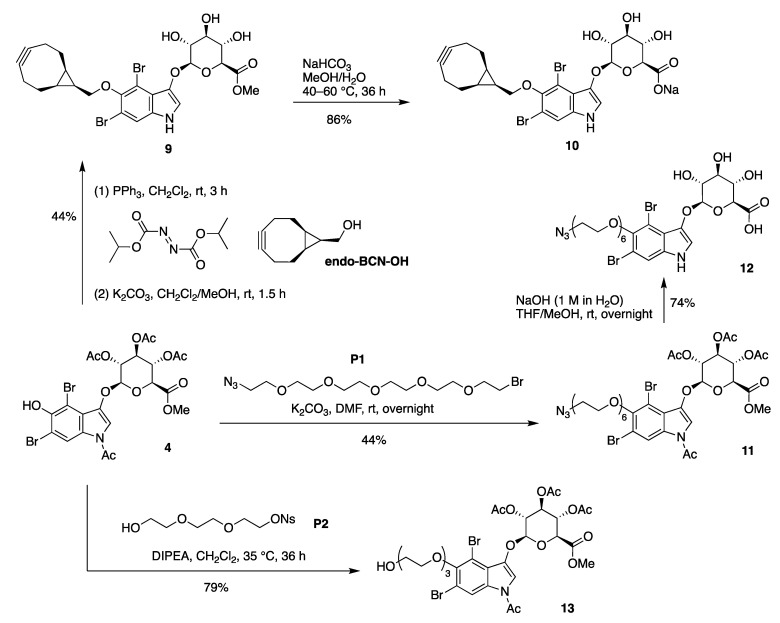
Introduction of the BCN, azido, and PEG groups.

The indoxyl-glucuronide scaffold **4** was derivatized to construct bioconjugatable compounds (Scheme 4). First, the Mitsunobu reaction [23,24] of (1*R*,8*S*,9*s*)-bicyclo [6.1.0]non-4-yn-9-ylmethanol (**endo-BCN-OH**) and **4** gave the corresponding BCN-tethered indoxyl-glucuronide methyl ester (isolated in 69% yield; not shown). Exposure to mild basic conditions (K_2_CO_3_ in CH_2_Cl_2_/methanol at room temperature for 1.5 h) caused exhaustive deacetylation without affecting the methyl ester to give **9** in 44% overall yield. Treatment of **9** with somewhat stronger conditions (NaHCO_3_ in aqueous methanol at 40–60 °C for 36 h) caused saponification to give the BCN-tethered indoxyl β-glucuronide sodium salt target compound **10** in 86% yield.

The installation of an azido-bearing tether was carried out by treatment of **4** with bromo-PEG_5_-azide (**P1**) in the presence of K_2_CO_3_ in *N*,*N*-dimethylformamide (DMF), which enabled alkylation of the phenolic hydroxy group without cleavage of the *N*-acetyl or *O*-acetyl protecting groups (Scheme 4). In this manner, the protected azido-PEG-indoxyl-glucuronide methyl ester **11** was obtained in 44% yield. Global deprotection was carried out under relatively strong basic conditions (aqueous NaOH in THF/methanol) to give target compound **12** in 74% yield. Treatment of **4** with nosyl-PEG_3_-OH (**P2**) [5] in the presence of *N*,*N*-diisopropylethylamine (DIPEA) gave **13** in 79% yield. 

**Scheme 5:**
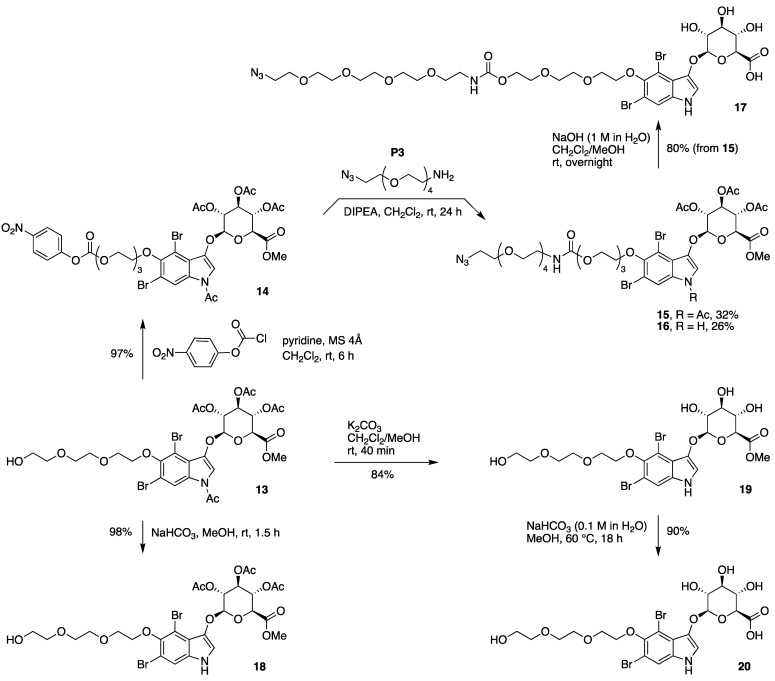
Synthesis of an azido-indoxyl-glucuronide with a longer spacer (**17**), and partial deprotection of **13**.

To install a longer spacer between the azido group and indoxyl moiety, the alcohol of the short PEG moiety of **13** was activated by treatment with 4-nitrophenyl chloroformate in the presence of pyridine to give carbonate **14** in 97% yield (Scheme 5). The reaction of **14** with the commercially available azido-PEG_4_-amine (**P3**) gave **15** and **16** in 32% and 26% yield, respectively, upon separation by preparative thin layer chromatography (TLC), where the latter product stems from the loss of the *N*-acetyl group under these conditions. Treatment of **15** with relatively strong basic conditions (aqueous NaOH in CH_2_Cl_2_/methanol) caused global deprotection and gave target compound **17** in 80% yield.

The indoxyl-glucuronide **13** is equipped with three types of protecting groups: one *N*-acetyl, three *O*-acetyl, and one ester *O*-methyl unit. Selective manipulation was achieved as follows (Scheme 5):

- Mild basic conditions (NaHCO_3_ suspended in methanol at room temperature for 1.5 h) gave selective cleavage of the *N*-acetyl group while leaving the *O*-acetyl groups intact, converting **13** to **18**;
- Somewhat stronger, but still mild, basic conditions (K_2_CO_3_ in CH_2_Cl_2_/methanol at room temperature for 40 min) gave exhaustive cleavage of the *N*-acetyl and *O*-acetyl groups, converting **13** to **19**;
- Stronger basic conditions (0.1 M aqueous NaHCO_3_ dissolved in methanol at 60 °C for 18 h) caused saponification of the methyl ester, converting **19** to the target compound **20**. In short, the aqueous methanol solution containing dissolved NaHCO_3_ affords a stronger basic condition than a suspension of NaHCO_3_ in methanol.

### 2.3. Synthesis of 5-, 6-, and 7-PEG Indoxyl β-D-Glucuronides

**Scheme 6:**
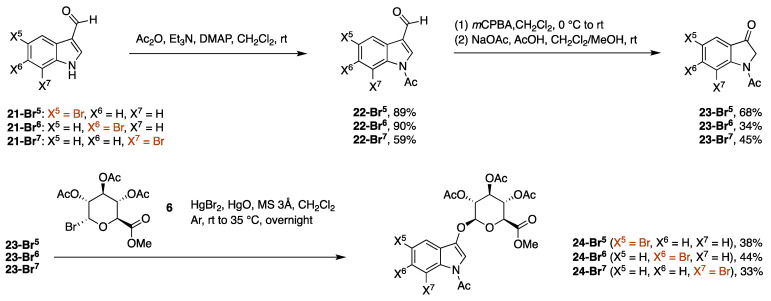
Synthesis of bromoindoxyl β-D-glucuronides.

The synthesis of 5-, 6-, and 7-PEG-ethynyl indoxyl-glucuronides started with the *N*-acetylation of the corresponding 5-, 6-, and 7-bromo derivatives of 3-formylindole (Scheme 6). Thus, each of the commercially available 5-, 6-, or 7-bromo derivative of 3-formylindole (**21-Br^5^**, **21-Br^6^**, or **21-Br^7^**) was treated with acetic anhydride in the presence of triethylamine and 4-dimethylaminopyridine (DMAP) to give the *N*-acetyl derivative **22-Br^5^**, **22-Br^6^**, or **22-Br^7^** in 89%, 90%, or 59% yield, respectively. The poor solubility of **22-Br^5^** and **22-Br^6^** in CH_2_Cl_2_ enabled simple purification by washing the crude product with a small amount of cold CH_2_Cl_2_, whereas **22-Br^7^** was purified by column chromatography. The Baeyer-Villiger oxidation of **22-Br^5^** was previously reported by Bourlot et al. [25] and applied here to all three substrates with minor modifications. Thus, **22-Br^5^**, **22-Br^6^**, or **22-Br^7^** was treated with *m*-chloroperbenzoic acid (*m*CPBA) followed by sodium acetate in acetic acid/CH_2_Cl_2_/methanol to give the indolinone **23-Br^5^**, **23-Br^6^**, or **23-Br^7^** in 68%, 34%, or 45% yield, respectively. We note that the indolyl formate (not shown) produced upon Baeyer-Villiger oxidation did not readily convert to the indolinone after silica preparative column chromatography, as described in the previous study [25]. Treatment of the indolyl formate with sodium acetate in a 5–10% acetic acid solution in CH_2_Cl_2_/methanol at <0.03 M afforded the indolinone without contaminating byproducts such as the *N*-deacetylated indole or the indigoid dye.

The procedure for glucosidation of *N*-acetyl indolinones [6] was applied with minor modifications to generate the indoxyl β-D-glucuronides. Thus, the glucuronidation of **23-Br^5^**, **23-Br^6^,** or **23-Br^7^** with acetobromo-α-D-glucuronic acid methyl ester in the presence of mercury(II) oxide and mercury(II) bromide in CH_2_Cl_2_ gave **24-Br^5^**, **24-Br^6^,** or **24-Br^7^** in 38%, 44%, or 33% yield, respectively. In each case, the isolated indoxyl-glucuronide did not show any of the unwanted indoxyl α-D-glucuronide anomer upon analysis via ^1^H NMR spectroscopy.

**Scheme 7:**
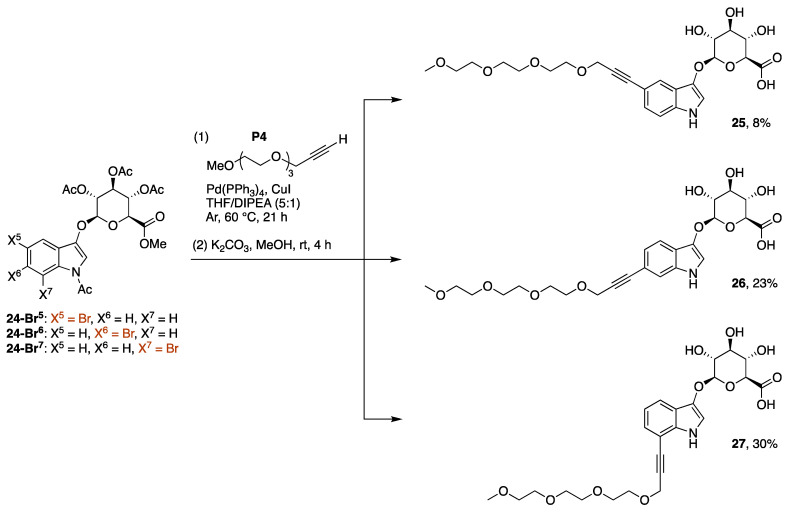
Synthesis of a 5-, 6-, or 7-PEGylated indoxyl-glucuronide.

The Sonogashira coupling reaction [26] was carried out by treating each indoxyl β-D-glucuronide (**24-Br^5^**, **24-Br^6^**, or **24-Br^7^**) with propargyl-PEG_3_-OMe (**P4**) in the presence of palladium(0) tetrakis(triphenylphosphine) and copper iodide at 60 °C. Subsequent exposure to a basic condition (a suspension of K_2_CO_3_ in methanol at room temperature for 4 h) caused exhaustive deacetylation as well as saponification to afford the 5-, 6-, or 7-PEGylated indoxyl β-D-glucuronide (**25**, **26**, or **27**) in 8%, 23%, or 30% yield, respectively (Scheme 7). The hydrolysis of the methyl ester was surprising, albeit desirable, and may stem from adventitious water in the methanol employed.

**Scheme 8:**
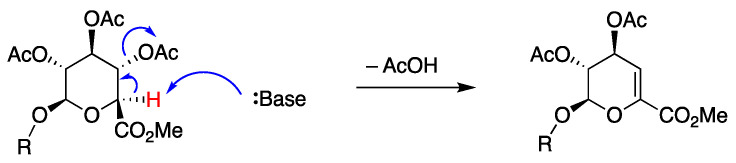
Proposed elimination process leading to an unsaturated glucuronide.

The presence of a byproduct was observed after Sonogashira coupling and exhaustive deacetylation reactions. In the synthesis of **25**, normal phase silica chromatography was not effective for separating **25** and the byproduct due to their similar chromatographic mobilities; however, C_18_-reversed phase silica chromatography (H_2_O/acetonitrile) enabled better separation. The byproduct in crude form was assigned to the structure **25-elim** on the basis of ^1^H NMR spectroscopy and LC-MS analysis (*m/z* = 491.18 for **25-elim** versus *m/z* = 509.19 for **25**). The ^1^H NMR spectrum of **25-elim** showed a broad singlet (5.92 ppm) assigned to H^1^, an apparent doublet (5.29 ppm) assigned to H^4^, and the absence of the anomeric proton (H^1^) at 4.68 ppm characteristic of **25** (see the Supplementary Materials, Figure S4). A byproduct of this type is precedented [27] in glucuronide chemistry and is readily understood to form by deprotonation of the acidic proton α- to the carbonyl group followed by β-elimination (shown for acetate elimination in Scheme 8). The ^1^H NMR data for the crude reaction product following deprotection was observed for each reaction leading to **25**–**27**. While we did not study this issue in depth, the elimination is believed to occur during the Sonogashira reaction. In the reaction of **24-Br^7^**, the ratio of the corresponding **27** and **27-elim** was 4:1 upon reaction at 60 °C versus 2:3 at 80 °C, as indicated by LC-MS data (see the Supplementary Materials, Figure S7). 

### 2.4. Enzymatic Tests

Enzymatic assays were performed to evaluate the efficacy of indigoid formation depending on the nature of the various substituents. Three enzyme treatments were employed: β-glucuronidase from bovine liver (at pH 5.0), β-glucuronidase from *Escherichia coli* (at pH 7.0), and rat liver tritosomes (hepatic lysosomes that have been loaded with Triton WR 1339; examined at pH 4.9). The reaction time and enzyme concentration were determined by time course and enzyme concentration-dependent studies (see the Supplementary Materials, Figures S1–S3). In general, 40 U/mL of enzyme sufficed, and the half-life for indigoid formation was 5 h (pH 5.0) or <30 min (pH 7.0). A mixture of indoxyl-glucuronide (100 μM) and β-glucuronidase (40 U/mL) or rat liver tritosomes (0.125 mg protein/mL) in the corresponding buffer with 1% dimethyl sulfoxide (DMSO) was incubated at 37 °C for 24 h. The resulting indigoid dye often precipitates, in which case the product was dissolved in an organic solvent for quantitative evaluation. The yields of indigoid are listed in Table 1.

The key findings are as follows:

- The standard control compound (**VI**, 4-chloro-5-bromo-1*H*-indol-3-yl β-D-glucopyranosiduronic acid, the glucuronide analogue of **II**) gave yields of 75%, 133%, and 35% under the three conditions (β-glucuronidase from bovine liver or *E. coli* and use of rat tritosomes, respectively). The yield >100% must reflect inaccuracies in the molar absorption coefficient value or experimental error.
- The 4,6-dibromo-5-alkoxyindoxyl-glucuronide methyl esters (**13**, **18**, and **19**) bearing 4, 3, or 0 acetyl groups, respectively, gave little or no indigoid formation, indicating the necessity for the carboxylic acid of the glucuronide moiety. No indigoid was observed even with the inclusion of an esterase in a cocktail experiment (Table S1). On the other hand, the fully deprotected analogue 4,6-dibromo-5-alkoxyindoxyl-glucuronide (**20**) gave a good yield of indigoid under the three conditions.
- Compound **20** contains a short PEG group. Two other 4,6-dibromo-5-alkoxyindoxyl-glucuronides bearing PEG groups (**12** and **17**) gave reasonable yields, but the shorter linker design of **12** afforded up to a 2.5-fold higher indigoid yield compared to the longer PEG linker compound (**17**) under acidic conditions. A more pronounced distinction occurred with the BCN-indoxyl-glucuronide (**10**), which gave no observable indigoid, an effect attributed to the presence of the bulky BCN group. In general, indigoid formation is more favorable (higher yield and shorter t_1/2_) under neutral (pH 7.0) or basic versus acidic (pH 5.0) conditions [28,29]. The time course for indigoid formation upon reaction of **12** is shown in Figures S2 and S3.
- The indoxyl-glucuronide isomers **25**, **26,** and **27** gave high, if not quantitative, indigoid yields under neutral conditions but yielded ~1/5 of that under acidic conditions. With tritosomes, the indigoid yield was 3- and 4-fold higher for **26** versus **27** and **25**. On the basis of these results, installation of the ethynyl group at the indoxyl 6-position is somewhat more favorable versus the 5- or 7-positions for indigoid formation.

**Table 1:** Enzymatic test of all glucuronide monomers.

Compd	Structural Feature	Yields of Indigoid Product *^a^*		
		β-Glucuronidase (bovine, pH 5.0)	β-Glucuronidase (*E. coli*, pH 7.0)	Tritosomes (pH 4.9)
**VI** *^b^*	Standard control	75% *^c^*	133% *^c^*	35% *^c^*
**13**	methyl ester	– *^d^*	<1% *^e^*	<1% *^e^*
**18**	methyl ester	– *^d^*	<1% *^e^*	<1% *^e^*
**19**	methyl ester	<1% *^e^*	18 ± 2%	<1% *^e^*
**20**	4,6-Br_2_-5-PEG	53 ± 1%	109 ± 3%	87%
**10**	4,6-Br_2_-5-hindered	<1% *^e^*	<1% *^e^*	– *^d^*
**12**	4,6-Br_2_-5-PEG	69 ± 6%	88 ± 9%	33 ± 1%
**17**	4,6-Br_2_-5-PEG	26 ± 3%	78 ± 5%	14 ± 2%
**25** *^b^*	5-ethynyl	16 ± 2% (22 ± 3%) *^f^*	81 ± 9% (106 ± 12%) *^f^*	9.3 ± 2.4% (12 ± 3%) *^f^*
**26** *^b^*	6-ethynyl	20 ± 5% (21 ± 5%) *^g^*	97 ± 11% (101 ± 11%) *^g^*	38 ± 1% (40 ± 1%) *^g^*
**27** *^b^*	7-ethynyl	7.4 ± 0.5% (11 ± 1%) *^h^*	60 ± 2% (93 ± 4%) *^h^*	11 ± 3% (18 ± 4%) *^h^*

*^a^* The yield was estimated from absorption spectroscopy following centrifugation and solubilization of any precipitates in DMF/H_2_O (2:1) with ε = 2.6 × 10^4^ M^−1^ cm^−1^ (measured for 4,4′6,6′-tetrabromo-5,5′-bis(8-hydroxy-3,6-dioxaoctyloxy)indigo) [5] for the dominant red-region absorption band in each case unless noted otherwise. *^b^* The precipitate (indigoid) was dissolved in DMF for absorbance measurements. *^c^* Calculated with ε = 2.00 × 10^4^ M^−1^ cm^−1^ reported for 5,5′-dibromo-4,4′-dichloroindigo [30]. *^d^* Not conducted. *^e^* No blue color was observed by visual inspection, and no absorption peak was observed spectroscopically. *^f^* Calculated with ε = 19,800 M^−1^ cm^−1^ (DMF) measured for **Ind(25)_2_**. *^g^* Calculated with ε = 24,900 M^−1^ cm^−1^ (DMF) measured for **Ind(26)_2_**. *^h^* Calculated with ε = 16,900 M^−1^ cm^−1^ (DMF) measured for **Ind(27)_2_**.

**Figure 1:**
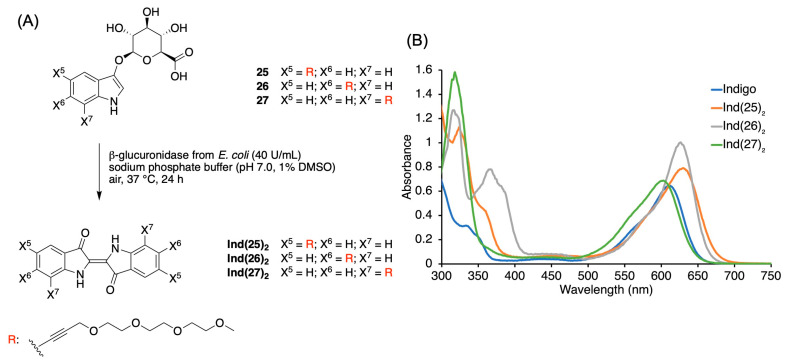
(**A**) Synthesis of indigoid derivatives from indoxyl-glucuronides. (**B**) Absorption spectra (40 μM in DMF, room temperature) of indigoid derivatives derived from **25**–**27**, as well as that of indigo (which lacks any substituents: X^5^ = X^6^ = X^7^ = H).

Reactions of **25**, **26,** or **27** (~2 mg each) were carried out with β-glucuronidase from *E. coli* under neutral conditions to obtain the corresponding indigoid **Ind(25)_2_**, **Ind(26)_2_**, or **Ind(27)_2_** (Figure 1). The isolated yields of 93%, 94%, or 90% were in accordance with those determined by absorption spectroscopy using a generic value of the molar absorption coefficient (26,000 M^−1^ cm^−1^) in Table 1. All three indigoid derivatives exhibited a characteristic indigoid absorption peak in DMF solution. The absorption peak (630, 626, and 603 nm) for **Ind(25)_2_**–**Ind(27)_2_** changes as the ethynyl substituent position changes from 5–7 on the indole ring (Figure 1). The molar absorption coefficient of each indigoid was determined in DMF and found to be 19,800 M^−1^ cm^−1^ (**Ind(25)_2_**, 24,900 M^−1^ cm^−1^ (**Ind(26)_2_**), and 16,900 M^−1^ cm^−1^ (**Ind(27)_2_**). The spectrum of indigo itself in DMF (610 nm, ε =16,000 M^−1^ cm^−1^) is also shown in Figure 1 for comparison. The yields in Table 1 were then calculated using the obtained molar absorption coefficient. Under the neutral condition (pH 7.0), the yield of indigoids was almost the same for all three substrates. However, **26** gave a better yield for the indigogenic reactions under the acidic condition compared to that of **25** or **27**, up to 3-fold.

## 3. Materials and Methods

### 3.1. General Methods

^1^H NMR and ^13^C{^1^H} NMR spectra were collected at room temperature unless noted otherwise. Chemical shifts for ^1^H NMR spectra are reported in parts per million (*δ*) relative to tetramethylsilane or a solvent signal [CD_3_OD, *δ* = 3.31 ppm] [31]. Chemical shifts for ^13^C{^1^H} NMR spectra are reported in parts per million (*δ*) relative to tetramethylsilane or a solvent signal [CD_3_OD, *δ* = 49.00 ppm; CDCl_3_, *δ* = 77.16 ppm] [31].

Silica (SiliaFlash^®^ P60, 40–63 μm, Silicycle, R12030B) was used for preparative column chromatography unless noted otherwise. Buchi Pure C810 or C850 with silica (50 μm, irregular) or C_18_-reversed phase silica (50 μm, spherical) was used for automated column chromatography. Preparative TLC separations were carried out on Merck analytical plates precoated with silica gel 60 F_254_.

All solvents were reagent grade and were used as received unless noted otherwise. *m*-Chloroperbenzoic acid (*m*CPBA, 75%) was purchased from Oakwood Chemical and purified [32] prior to use, unless noted otherwise.

β-glucuronidase from bovine liver and β-glucuronidase from *E. coli* were purchased from Sigma. The enzymatic activity of β-glucuronidase was determined by the standard substrate, phenolphthalein β-D-glucuronide. Rat liver tritosomes and 10X catabolic buffer were purchased from Xenotech.

Commercial compounds were used as received, unless noted otherwise. Known compounds **1**, **5,** and **P2** were prepared as described in the literature [5,6].

### 3.2. Synthesis

*1-Acetyl-4,6-dibromo-5-hydroxy-1H-indol-3-yl 2,3,4-tri-O-acetyl-β-D-glucopyranoside* (**2**). Following a general procedure [20] with slight modification, dichlorotetrakis(1,1-dimethylethyl)di-*μ*-hydroxyditin [21] (6.8 mg, 0.012 mmol) was added to a solution of **1** (81.5 mg, 0.12 mmol) in methanol/CHCl_3_ (0.80 mL, 5:3) at room temperature. After 12 h, the reaction mixture was diluted with ethyl acetate and passed through silica (ethyl acetate as the eluent). The eluent was concentrated under reduced pressure. Column chromatography [silica, hexanes/acetone (2:1)] followed by trituration with hexanes/acetone afforded a white solid (46.7 mg, 61%): ^1^H NMR (700 MHz, CD_3_OD) *δ* 8.54 (s, 1H), 7.50 (s, 1H), 5.38 (dd, *J* = 9.0, 9.5 Hz, 1H), 5.32 (dd, *J* = 8.3, 9.0 Hz, 1H), 5.22 (d, *J* = 8.3 Hz, 1H), 5.08 (dd, *J* = 9.5, 9.7 Hz, 1H), 3.97–3.90 (m, 1H), 3.75 (dd, *J* = 1.5, 12.1 Hz, 1H), 3.65 (dd, *J* = 6.9, 12.1 Hz, 1H), 2.55 (s, 3H), 2.08 (s, 3H), 2.07 (s, 3H), 2.00 (s, 3H); ^13^C{^1^H} NMR (175 MHz, CD_3_OD) *δ* 171.7, 171.3, 171.2, 170.4, 148.9, 141.8, 129.6, 123.9, 120.8, 112.9, 110.9, 101.4, 100.8, 76.3, 74.6, 72.5, 70.3, 61.9, 23.6, 21.0, 20.58, 20.57; ESI-MS obsd 657.9532, calcd 657.9530 [(M + Na)^+^, M = C_22_H_23_Br_2_NO_11_].

*1-Acetyl-4,6-dibromo-5-(tert-butyldimethylsilyloxy)-1H-indol-3-yl 2,3,4-tri-O-acetyl-β-D-glucopyranoside* (**3**). A sample of triethylamine (14.9 μL, 0.107 mmol) was added to a suspension of **2** (34.1 mg, 0.0535 mmol) and *tert*-butylchlorodimethylsilane (16.1 mg, 0.107 mmol) in CH_2_Cl_2_ (535 μL) at room temperature. After 18 h, trifluoroacetic acid (16.4 μL, 0.214 mmol), pyridine (4.3 μL, 0.53 mmol), and methanol (535 μL) were added. After 5 h, the reaction mixture was diluted with ethyl acetate and passed through silica (ethyl acetate as the eluent). The eluent was concentrated under reduced pressure. Column chromatography [silica, hexanes/ethyl acetate (2:3)] afforded a white solid (26.3 mg, 65%): ^1^H NMR (700 MHz, CDCl_3_) *δ* 8.57 (br s, 1H), 7.22 (s, 1H), 5.37 (dd, *J* = 8.6, 9.3 Hz, 1H), 5.32 (dd, *J* = 8.6, 9.3 Hz, 1H), 5.15 (dd, *J* = 9.3, 9.3 Hz, 1H), 4.99 (d, *J* = 7.8 Hz, 1H), 3.82–3.75 (m, 3H), 2.67 (br s, 1H), 2.52 (s, 3H), 2.08 (s, 3H), 2.07 (s, 3H), 2.05 (s, 3H), 1.05 (s, 9H), 0.36 (s, 3H), 0.35 (s, 3H); ^13^C{^1^H} NMR (175 MHz, CDCl_3_) *δ* 170.3, 170.0, 169.5, 167.9, 147.0, 140.9, 129.1, 122.9, 120.4, 114.4, 110.9, 104.5, 100.6, 75.0, 72.7, 70.8, 68.7, 61.4, 29.7, 26.3, 23.6, 20.9, 20.6, 19.0, -2.0; ESI-MS obsd 750.0581, calcd 750.0575 [(M + H)^+^, M = C_28_H_37_Br_2_NO_11_Si].

*1-Acetyl-4,6-dibromo-5-hydroxy-1H-indol-3-yl 2,3,4-tri-O-acetyl-β-D-glucopyranosiduronic acid methyl ester* (**4**, from **3**). Following a reported procedure [19] with modification, (diacetoxyiodo)benzene (26.4 mg, 82 μmol) was added to a suspension of **3** (28.0 mg, 37 μmol), 2,2,6,6-tetramethylpiperidine 1-oxyl (1.7 mg, 11 μmol), and NaHCO_3_ (3.1 mg, 37 μmol) in acetonitrile/H_2_O (3:1, 273 μL) at room temperature. After 3 h, NaHCO_3_ (6.2 mg, 74 μmol) was added. After 2 h, 2,2,6,6-tetramethylpiperidine 1-oxyl (1.2 mg, 7.7 μmol) was added. After 1.5 h, (diacetoxyiodo)benzene (12.0 mg, 37 μmol) was added. After 30 min, NaHCO_3_ (15.7 mg, 0.19 mmol) and dimethyl sulfate (28.3 μL, 0.30 mmol) were added. After 4 h, the reaction mixture was diluted with ethyl acetate and passed through silica (ethyl acetate as the eluent). The eluent was concentrated under reduced pressure. The resulting residue was dissolved in THF (317 μL). Acetic acid (4.3 μL, 75 μmol) and TBAF (1.0 M in THF, 56 μL, 56 μmol) were added at room temperature. After 2 h, the reaction mixture was diluted with ethyl acetate and passed through silica (ethyl acetate as the eluent). The eluent was concentrated under reduced pressure. Column chromatography [silica, hexanes/acetone (3:2)] afforded a pale brown solid (8.2 mg, 33%): mp 202–204 °C; ^1^H NMR (700 MHz, CDCl_3_) *δ* 8.67 (br s, 1H), 7.32 (s, 1H), 6.00 (br s, 1H), 5.47–5.32 (m, 3H), 5.13 (d, *J* = 6.8 Hz, 1H), 4.26 (d, *J* = 9.2 Hz, 1H), 3.78 (s, 3H), 2.57 (s, 3H), 2.10 (s, 3H), 2.07 (s, 6H); ^13^C{^1^H} NMR (175 MHz, CDCl_3_) *δ* 170.1, 169.3, 169.2, 167.9, 166.8, 146.2, 139.8, 128.6, 122.4, 120.0, 112.2, 108.5, 100.2, 98.2, 72.5, 71.9, 70.6, 68.8, 53.2, 23.6, 20.9, 20.64, 20.55; ESI-MS obsd 685.9469, calcd 685.9479 [(M + Na)^+^, M = C_23_H_23_Br_2_NO_12_].

*1-Acetyl-5-benzyloxy-1H-indol-3-yl 2,3,4-tri-O-acetyl-β-D-glucopyranosiduronic acid methyl ester* (**7**). Following a reported method [6] with some modification, activated molecular sieves 4Å (250 mg), **5** (557 mg, 2.0 mmol), **6** (2.4 g, 6.0 mmol), and mercury(II) oxide (645 mg, 3.0 mmol) were placed in a flask and treated under argon with toluene/nitromethane (4:1, 20 mL). The resulting orange suspension was then treated with mercury(II) bromide (7.0 mg, 19 μmol), heated to 30 °C, and stirred for 12 h. The reaction was quenched by the addition of pyridine (2.0 mL). The mixture was filtered through a silica pad (2 cm × 2 cm, CH_2_Cl_2_/acetone 1:1). The filtrate was concentrated and subjected to column chromatography [silica, 1% to 3% acetone in CH_2_Cl_2_], followed by recrystallization (hexanes/CH_2_Cl_2_), which afforded a white solid (583 mg, 49%): mp 189–191 °C; ^1^H NMR (600 MHz, CDCl_3_) *δ* 8.31 (br s, 1H), 7.46 (d, *J* = 7.3 Hz, 2H), 7.39 (t, *J* = 7.7 Hz, 2H), 7.33 (t, *J* = 7.3 Hz, 1H), 7.15 (br s, 1H), 7.07–7.01 (m, 2H), 5.41–5.30 (m, 3H), 5.11 (s, 2H), 5.08 (d, *J* = 7.0 Hz, 1H), 4.19 (d, *J* = 8.9 Hz, 1H), 3.74 (s, 3H), 2.55 (s, 3H), 2.08 (s, 3H), 2.07 (s, 3H), 2.05 (s, 3H); ^13^C{^1^H} NMR (150 MHz, CDCl_3_) *δ* 170.1, 169.3, 169.1, 167.9, 166.8, 155.6, 141.1, 136.9, 128.6, 128.0, 127.5, 124.7, 117.7, 115.7, 110.6, 101.5, 100.8, 72.7, 71.7, 70.9, 70.5, 69.0, 53.1, 23.7, 20.7, 20.6, 20.5 (one expected carbon is missing); ESI-MS obsd 620.1742, calcd 620.1739 [(M + Na)^+^, M = C_30_H_31_NO_12_].

*1-Acetyl-5-hydroxy-1H-indol-3-yl 2,3,4-tri-O-acetyl-β-D-glucuronic acid methyl ester* (**8**). A suspension of **7** (170 mg, 0.28 mmol) and Pd/C (10 wt%, 30 mg, 28 μmol) in CH_2_Cl_2_/ethanol/THF (4:1:5, 11 mL) was stirred under an atmosphere of H_2_ (1 atm) for 1 h. The reaction mixture was filtered through a silica pad (2 cm × 2 cm, acetone). The filtrate was concentrated and recrystallized (hexanes/CH_2_Cl_2_) to afford a white solid (106 mg, 75%): mp 200–202 °C; ^1^H NMR (600 MHz, CDCl_3_) *δ* 8.24 (br s, 1H), 7.10 (br s, 1H), 6.92 (d, *J* = 2.5 Hz, 1H), 6.89 (dd, *J* = 8.9, 2.5 Hz, 1H), 6.13 (s, 1H), 5.41–5.32 (m, 3H), 5.07 (d, *J* = 7.1 Hz, 1H), 4.22 (d, *J* = 9.2 Hz, 1H), 3.76 (s, 3H), 2.54 (s, 3H), 2.10 (s, 3H), 2.07 (s, 3H), 2.06 (s, 3H); ^13^C{^1^H} NMR (150 MHz, CDCl_3_) *δ* 170.2, 169.5, 169.4, 168.2, 167.0, 152.7, 141.0, 128.2, 125.0, 117.7, 115.0, 110.6, 102.9, 100.7, 72.5, 71.8, 70.9, 69.0, 53.1, 23.6, 20.7, 20.6, 20.5; ESI-MS obsd 530.1264, calcd 530.1269 [(M + Na)^+^, M = C_23_H_25_NO_12_].

*1-Acetyl-4,6-dibromo-5-hydroxy-1H-indol-3-yl 2,3,4-tri-O-acetyl-β-D-glucopyranosiduronic acid methyl ester* (**4**, from **8**). Following a reported method [5] with some modification, a solution of NBS (156 mg, 0.88 mmol) in CH_2_Cl_2_ (10.0 mL) was added dropwise over 30 min to a solution of **8** (215 mg, 0.42 mmol) and 2,6-DTBP (92 μL, 0.42 mmol) in CH_2_Cl_2_ (6.0 mL) at −78 °C. The reaction mixture was allowed to warm to room temperature, stirred for 2.5 h, and quenched by the addition of 10% aqueous Na_2_S_2_O_3_. The mixture was washed with brine, dried (Na_2_SO_4_), and concentrated. Column chromatography (silica, CH_2_Cl_2_ with 1% to 4% acetone) afforded a white solid (242 mg, 87%): the ^1^H NMR data were consistent with the product from **3**; ESI-MS obsd 685.9489, calcd 685.9479 [(M + Na)^+^, M = C_23_H_23_Br_2_NO_12_].

*5-{[(1R,8S,9s)-Bicyclo [6.1.0]non-4-yn-9-yl]methoxy}-4,6-dibromo-1H-indole-3-yl β-D-glucopyranosiduronic acid methyl ester* (**9**). In an initial procedure, diisopropyl azodicarboxylate (13 μL, 63 μmol) was added to a solution containing **4** (34 mg, 51 μmol), **endo-BCN-OH** (9.3 mg, 62 μmol), and PPh_3_ (17 mg, 65 μmol) in CH_2_Cl_2_ (0.51 mL) at room temperature. The reaction mixture was stirred for 1 h and then quenched by the addition of H_2_O. The organic layer was washed with brine, dried (Na_2_SO_4_), and concentrated. Column chromatography (silica, hexanes with 0% to 4% acetone) afforded the Mitsunobu coupling product (1-acetyl-5-{[(1*R*,8*S*,9*s*)-bicyclo [6.1.0]non-4-yn-9-yl]methoxy}-4,6-dibromo-5-hydroxy-1*H*-indol-3-yl 2,3,4-tri-*O*-acetyl-β-D-glucopyranosiduronic acid methyl ester) as a white solid (28 mg, 69%), which was characterized by ^1^H NMR spectroscopy (500 MHz, CDCl_3_): *δ* 8.71 (s, 1H), 7.35 (s, 1H), 5.47–5.31 (m, 3H), 5.14 (d, *J* = 7.0 Hz, 1H), 4.27 (d, *J* = 9.6 Hz, 1H), 4.10 (d, *J* = 7.8 Hz, 2H), 3.78 (s, 3H), 2.58 (s, 3H), 2.37–2.28 (m, 4H), 2.28–2.20 (m, 2H), 2.11 (s, 3H), 2.07 (s, 3H), 2.06 (s, 3H), 1.80–1.65 (m, 3H), 1.10–1.01 (m, 2H). In a subsequent procedure to directly obtain the title compound via a one-flask process, diisopropyl azodicarboxylate (3.5 μL, 0.018 mmol) was added to a solution of **4** (7.0 mg, 0.11 mmol), **endo-BCN-OH** (1.9 mg, 0.013 mmol), and PPh_3_ (4.7 mg, 0.018 mmol) in CH_2_Cl_2_ (105 μL) at room temperature. After 3 h, methanol (420 μL) and K_2_CO_3_ (1.5 mg) were added. After 1.5 h, the reaction mixture was passed through silica [CH_2_Cl_2_/methanol (2:1)]. The eluent was concentrated under reduced pressure. Preparative thin layer chromatography [silica, 0.25 mm thick, 20 cm wide × 10 cm tall, CHCl_3_/methanol (10:1)] afforded the title compound as a white solid (2.9 mg, 44%): ^1^H NMR (700 MHz, CD_3_OD) *δ* 7.50 (s, 1H), 7.12 (s, 1H), 4.82 (d, *J* = 8.0 Hz, 1H), 4.09 (d, *J* = 7.8 Hz, 2H), 3.96 (d, *J* = 9.6 Hz, 1H), 3.78 (s, 3H), 3.66 (dd, *J* = 9.4, 9.6 Hz, 1H), 3.58 (dd, *J* = 8.0, 8.8 Hz, 1H), 3.49 (dd, *J* = 8.8, 9.4 Hz, 1H), 2.34–2.22 (m, 4H), 2.22–2.13 (m, 2H), 1.78–1.65 (m, 3H), 1.09–0.98 (m, 2H); ^13^C{^1^H} NMR (175 MHz, CD_3_OD) *δ* 171.1, 147.1, 138.5, 132.9, 120.1, 116.1, 115.4, 112.7, 107.9, 105.3, 99.7, 77.4, 76.8, 75.0, 73.0, 72.8, 52.9, 30.7, 22.0, 21.8, 20.2; ESI-MS obsd 650.0000, calcd 649.9996 [(M + Na)^+^, M = C_25_H_27_Br_2_NO_8_].

*Sodium 5-{[(1R,8S,9s)-bicyclo [6.1.0]non-4-yn-9-yl]methoxy}-4,6-dibromo-1H-indole-3-yl β-D-glucopyranosiduronate* (**10**). Aqueous NaHCO_3_ (100 mM, 46 μL) was added to a solution of **9** (2.9 mg, 0.0046 mmol) in methanol (184 μL) at room temperature. The reaction mixture was heated to 40 °C for 13 h and then to 60 °C for 23 h. The reaction mixture was allowed to cool to room temperature and then concentrated under reduced pressure to afford a white solid (2.5 mg, 86%): ^1^H NMR (700 MHz, CD_3_OD) *δ* 7.49 (s, 1H), 7.35 (s, 1H), 4.73 (d, *J* = 8.1 Hz, 1H), 4.09 (d, *J* = 7.8 Hz, 2H), 3.68 (d, *J* = 9.5 Hz, 1H), 3.59 (dd, *J* = 8.1, 8.8 Hz, 1H), 3.56 (dd, *J* = 9.2, 9.5 Hz, 1H), 3.51 (dd, *J* = 8.8, 9.2 Hz, 1H), 2.33–2.24 (m, 4H), 2.21–2.15 (m, 2H), 1.77–1.66 (m, 3H), 1.07–0.98 (m, 2H); ^13^C{^1^H} NMR (175 MHz, CD_3_OD) *δ* 176.6, 146.8, 138.7, 132.8, 120.0, 116.3, 115.9, 112.3, 107.8, 105.2, 99.6, 77.9, 76.6, 75.1, 73.7, 72.8, 30.6, 22.0, 21.7, 20.1; ESI-MS obsd 635.9840, calcd 635.9839 [(M + H)^+^, M = C_24_H_24_Br_2_NNaO_8_].

*1-Acetyl-5-(17-azido-3,6,9,12,15-pentaoxaheptadecyloxy)-4,6-dibromo-1H-indol-3-yl 2,3,4-tri-O-acetyl-β-D-glucopyranosiduronic acid methyl ester* (**11**). A suspension of **4** (92 mg, 0.14 mmol) and K_2_CO_3_ (23 mg, 0.17 mmol) in DMF (3.1 mL) was treated with **P1** (0.10 g, 0.29 mmol) and stirred overnight at room temperature. The reaction mixture was diluted with ethyl acetate (20 mL), washed with H_2_O (20 mL × 3) and brine (20 mL × 2), dried over anhydrous Na_2_SO_4_, and concentrated. The crude mixture was chromatographed (silica, CH_2_Cl_2_/ethyl acetate = 4:1 to 2:1) to obtain a white non-crystalline solid (58 mg, 44%): ^1^H NMR (500 MHz, CDCl_3_) *δ* 8.71 (s, 1H), 7.36 (s, 1H), 5.46–5.30 (m, 3H), 5.13 (d, *J* = 7.0 Hz, 1H), 4.26 (d, *J* = 9.6 Hz, 1H), 4.21–4.15 (m, 2H), 3.96 (t, *J* = 5.0 Hz, 2H), 3.81–3.76 (m, 5H), 3.72–3.65 (m, 16H), 3.39 (t, *J* = 5.1 Hz, 2H), 2.58 (s, 3H), 2.10 (s, 3H), 2.06 (s, 6H); ^13^C{^1^H} NMR (125 MHz, CDCl_3_) *δ* 170.1, 169.3, 169.1, 167.9, 166.8, 149.7, 140.1, 130.9, 123.0, 120.3, 116.2, 112.4, 107.5, 100.2, 72.5, 72.4, 71.9, 70.8, 70.68, 70.66, 70.62, 70.59, 70.58, 70.56, 70.1, 70.0, 68.8, 53.1, 50.7, 23.7, 20.9, 20.6, 20.5 (two expected carbons are missing); ESI-MS obsd 975.1123, calcd 975.1117 [(M + Na)^+^, M = C_35_H_46_Br_2_N_4_O_17_].

*5-(17-Azido-3,6,9,12,15-pentaoxaheptadecyloxy)-4,6-dibromo-1H-indol-3-yl β-D-glucopyranosiduronic acid* (**12**). A solution of **11** (15 mg, 16 μmol) in THF/methanol (1:1, 0.50 mL) was treated with aqueous NaOH (1 M, 16 μL) and stirred overnight at room temperature. The reaction mixture was concentrated and chromatographed [silica, CH_2_Cl_2_/methanol (5:1 to 2:1)] to afford a pale-yellow non-crystalline solid (9.0 mg, 74%): ^1^H NMR (500 MHz, CD_3_OD) *δ* 7.51 (s, 1H), 7.30 (s, 1H), 4.76 (d, *J* = 7.8 Hz, 1H), 4.15 (t, *J* = 5.0 Hz, 2H), 3.95 (t, *J* = 5.0 Hz, 2H), 3.81–3.77 (m, 2H), 3.75 (d, *J* = 9.7 Hz, 1H), 3.72–3.57 (m, 18H), 3.51 (t, *J* = 9.0 Hz, 1H), 3.36 (t, *J* = 5.1 Hz, 2H); ^13^C{^1^H} NMR (125 MHz, CD_3_OD) *δ* 175.4, 146.7, 138.6, 132.8, 119.9, 116.0, 112.1, 107.6, 105.1, 77.7, 76.6, 75.0, 73.6, 73.5, 71.6, 71.50, 71.47, 71.45, 71.42, 71.40, 71.2, 71.0, 51.7 (three expected carbons are missing); ESI-MS obsd 769.0583, calcd 769.0573 [(M − H)^−^, M = C_26_H_36_Br_2_N_4_O_13_].

*1-Acetyl-4,6-dibromo-5-(1-hydroxy-3,6,9-trioxanon-9-yl)-1H-indol-3-yl 2,3,4-tri-O-acetyl-β-D-glucopyranosiduronic acid methyl ester* (**13**). A sample of DIPEA (64 μL, 0.37 mmol) was added to a solution containing **4** (122 mg, 0.18 mmol) and **P2** (100 mg, 0.30 mmol) in CH_2_Cl_2_ (1.0 mL) at room temperature. The reaction mixture was stirred at 35 °C for 36 h and then concentrated. Column chromatography (silica, CH_2_Cl_2_ with 0% to 70% ethyl acetate) afforded a white non-crystalline solid (116 mg, 79%): ^1^H NMR (600 MHz, CDCl_3_) *δ* 8.71 (s, 1H), 7.35 (s, 1H), 5.47–5.37 (m, 2H), 5.34 (t, *J* = 8.9 Hz, 1H), 5.13 (d, *J* = 7.0 Hz, 1H), 4.26 (d, *J* = 9.7 Hz, 1H), 4.22–4.16 (m, 2H), 4.00–3.94 (m, 2H), 3.83–3.79 (m, 2H), 3.78 (s, 3H), 3.76–3.70 (m, 4H), 3.67–3.60 (m, 2H), 2.57 (s, 3H), 2.10 (s, 3H), 2.06 (s, 6H); ^13^C{^1^H} NMR (150 MHz, CDCl_3_) *δ* 170.1, 169.3, 169.2, 167.9, 166.8, 149.7, 140.1, 130.9, 123.0, 120.3, 116.2, 112.4, 107.5, 100.2, 72.52, 72.50, 72.4, 71.9, 70.9, 70.6, 70.5, 70.2, 68.8, 61.8, 53.1, 23.7, 20.9, 20.6, 20.5; ESI-MS obsd 818.0262, calcd 818.0266 [(M + Na)^+^, M = C_29_H_35_Br_2_NO_15_].

*1-Acetyl-4,6-dibromo-5-(1-(4-nitrophenoxycarbonyloxy)-3,6,9-trioxanon-9-yl)-1H-indol-3-yl 2,3,4-tri-O-acetyl-β-D-glucopyranosiduronic acid methyl ester* (**14**). A suspension of **13** (60 mg, 75 μmol), 4-nitrophenyl chloroformate (24 mg, 0.12 mmol), and activated molecular sieves 4Å (150 mg) in anhydrous CH_2_Cl_2_ (3.0 mL) was treated with pyridine (12 μL, 0.15 mmol) and stirred at room temperature for 6 h. The crude mixture was filtered through a silica pad (2 cm × 2 cm, ethyl acetate). The filtrate was concentrated and chromatographed (silica, CH_2_Cl_2_ with 0% to 20% ethyl acetate) to afford a white non-crystalline solid (70 mg, 97%); ^1^H NMR (500 MHz, CDCl_3_) *δ* 8.69 (s, 1H), 8.26–8.20 (m, 2H), 7.39–7.36 (m, 2H), 7.35 (s, 1H), 5.46–5.31 (m, 3H), 5.13 (d, *J* = 6.9 Hz, 1H), 4.48–4.43 (m, 2H), 4.27 (d, *J* = 9.7 Hz, 1H), 4.22–4.16 (m, 2H), 3.97 (t, *J* = 4.9 Hz, 2H), 3.88–3.84 (m, 2H), 3.84–3.81 (m, 2H), 3.79–3.74 (m, 5H), 2.57 (s, 3H), 2.10 (s, 3H), 2.06 (s, 6H); ^13^C{^1^H} NMR (125 MHz, CDCl_3_) *δ* 170.1, 169.3, 169.1, 167.9, 166.8, 155.5, 152.5, 149.7, 145.3, 140.0, 130.9, 125.2, 122.9, 121.7, 120.3, 116.2, 112.3, 107.5, 100.1, 72.5, 72.4, 71.9, 70.9, 70.8, 70.6, 70.2, 68.76, 68.75, 68.4, 53.1, 23.7, 20.9, 20.6, 20.5; ESI-MS obsd 983.0317, calcd 983.0328 [(M + Na)^+^, M = C_36_H_38_Br_2_N_2_O_19_].

*1-Acetyl-5-(11-aza-25-azido-3,6,9,14,17,20,23-heptaoxa-10-oxo-pentacosyloxy)-4,6-dibromo-1H-indol-3-yl 2,3,4-tri-O-acetyl-β-D-glucopyranosiduronic acid methyl ester* (**15**). A solution of **14** (24 mg, 25 μmol) and **P3** (12 μL, 50 μmol) in anhydrous CH_2_Cl_2_ (0.50 mL) was treated with DIPEA (8.7 μL, 50 μmol) and stirred at room temperature for 24 h. The crude mixture was concentrated and chromatographed (silica, CH_2_Cl_2_ to ethyl acetate) to afford a white non-crystalline solid (21 mg) as a mixture of **15** and **16**. This sample was further purified by preparative TLC [silica, 5 mm thick, 20 cm × 20 cm, hexanes/actetone (1:1), R_f_ = 0.2 for **15** and 0.15 for **16**]. Data for **15** (8.6 mg, 32%): ^1^H NMR (500 MHz, CDCl_3_) *δ* 8.72 (s, 1H), 7.36 (s, 1H), 5.46–5.28 (m, 4H), 5.13 (d, *J* = 7.0 Hz, 1H), 4.26 (d, *J* = 9.6 Hz, 1H), 4.22 (t, *J* = 4.8 Hz, 2H), 4.18 (t, *J* = 4.9 Hz, 2H), 3.96 (t, *J* = 5.0 Hz, 2H), 3.81–3.78 (m, 2H), 3.77 (s, 3H), 3.74–3.69 (m, 4H), 3.69–3.64 (m, 10H), 3.64–3.59 (m, 4H), 3.55 (t, *J* = 5.1 Hz, 2H), 3.41–3.34 (m, 4H), 2.57 (s, 3H), 2.10 (s, 3H), 2.06 (s, 3H), 2.06 (s, 3H); ^13^C{^1^H} NMR (125 MHz, CDCl_3_) δ 170.1, 169.3, 169.1, 167.9, 166.8, 156.5, 149.7, 140.1, 130.9, 123.0, 120.3, 116.2, 112.4, 107.5, 100.2, 72.6, 72.4, 71.9, 70.8, 70.71, 70.67, 70.62, 70.57, 70.5, 70.3, 70.2, 70.1, 70.0, 69.8, 68.8, 64.0, 53.1, 50.7, 40.8, 23.7, 20.9, 20.6, 20.5 (two expected carbons are missing); ESI-MS obsd 1084.1874, calcd 1084.1880 [(M + H)^+^, M = C_40_H_55_Br_2_N_5_O_20_]. Data for **16** (6.7 mg, 26%): ^1^H NMR (500 MHz, CDCl_3_) *δ* 8.23 (s, 1H), 7.48 (s, 1H), 7.14 (d, *J* = 2.7 Hz, 1H), 5.42–5.24 (m, 4H), 5.04 (d, *J* = 7.3 Hz, 1H), 4.25–4.13 (m, 5H), 3.95 (t, *J* = 5.1 Hz, 2H), 3.81–3.78 (m, 2H), 3.76 (s, 3H), 3.72–3.68 (m, 4H), 3.68–3.63 (m, 10H), 3.62–3.57 (m, 4H), 3.54–3.49 (m, 2H), 3.40–3.31 (m, 4H), 2.10 (s, 3H), 2.05 (s, 3H), 2.04 (s, 3H); ^13^C{^1^H} NMR (125 MHz, CDCl_3_) *δ* 170.1, 169.4, 169.3, 167.1, 156.5, 146.5, 136.6, 131.2, 119.0, 115.0, 114.7, 112.6, 106.9, 101.0, 72.6, 72.4, 72.2, 70.79, 70.76, 70.7, 70.58, 70.55, 70.5, 70.3, 70.1, 70.0, 69.7, 69.3, 64.0, 53.0, 50.7, 40.8, 21.0, 20.7, 20.5 (three expected carbons are missing); ESI-MS obsd 1042.1788, calcd 1042.1774 [(M + H)^+^, M = C_38_H_53_Br_2_N_5_O_19_].

*5-(11-Aza-25-azido-3,6,9,14,17,20,23-heptaoxa-10-oxo-pentacosyloxy)-4,6-dibromo-1H-indol-3-yl β-D-glucopyranosiduronic acid* (**17**). A solution of **15** (8.6 mg, 7.9 μmol) in CH_2_Cl_2_/methanol (1:1, 0.50 mL) was treated with aqueous NaOH (1 M, 16 μL) and stirred overnight at room temperature. The reaction mixture was concentrated and purified by preparative TLC [silica, 5 mm thick, 20 cm × 20 cm, CH_2_Cl_2_/methanol (3:2), R_f_ = 0.45] to afford a white non-crystalline solid (5.7 mg, 80%): ^1^H NMR (500 MHz, CD_3_OD) *δ* 7.50 (s, 1H), 7.35 (s, 1H), 4.74 (d, *J* = 7.7 Hz, 1H), 4.23–4.11 (m, 4H), 3.94 (t, *J* = 4.8 Hz, 2H), 3.82–3.67 (m, 8H), 3.66–3.55 (m, 15H), 3.55–3.48 (m, 3H), 3.37 (t, *J* = 4.8 Hz, 2H), 3.28 (t, *J* = 5.5 Hz, 2H); ^13^C{^1^H} NMR (125 MHz, CD_3_OD) *δ* 159.0, 146.7, 138.8, 132.8, 120.0, 116.2, 116.0, 112.0, 107.6, 105.2, 77.9, 75.1, 73.7, 73.6, 71.73, 71.68, 71.52, 71.48, 71.46, 71.3, 71.2, 71.1, 71.0, 70.7, 65.2, 51.8, 41.7 (four expected carbons are missing); ESI-MS obsd 900.1177, calcd 900.1155 [(M − H)^−^, M = C_31_H_45_Br_2_N_5_O_16_].

*4,6-Dibromo-5-(1-hydroxy-3,6,9-trioxanon-9-yl)-1H-indol-3-yl 2,3,4-tri-O-acetyl-β-D-glucopyranosiduronic acid methyl ester* (**18**). A solution of **13** (2.8 mg, 3.5 μmol) in methanol (60 μL) was treated with NaHCO_3_ (0.031 mg, 0.35 μmol). The heterogeneous reaction mixture was stirred at room temperature for 90 min. The reaction mixture was concentrated. The resulting yellow oil was chromatographed (silica, hexanes/acetone = 1:1) to obtain a yellowish green oil (2.6 mg, 98%): ^1^H NMR (600 MHz, CDCl_3_) *δ* 7.80 (d, *J* = 2.7 Hz, 1H), 7.46 (s, 1H), 7.14 (d, *J* = 2.7 Hz, 1H), 5.41–5.32 (m, 3H), 5.04 (d, *J* = 7.4 Hz, 1H), 4.19–4.14 (m, 3H), 3.99–3.95 (m, 2H), 3.83–3.80 (m, 2H), 3.78–3.73 (m, 7H), 3.67–3.63 (m, 2H), 2.10 (s, 3H), 2.05 (s, 3H), 2.05 (s, 3H); ESI-MS obsd 776.0160, calcd 776.0160 [(M + Na)^+^, M = C_27_H_33_Br_2_NO_14_].

*4,6-Dibromo-5-(1-hydroxy-3,6,9-trioxanon-9-yl)-1H-indol-3-yl β-D-glucopyranosiduronic acid methyl ester* (**19**). A solution of **13** (15 mg, 19 μmol) in CH_2_Cl_2_/methanol (1:4, 0.95 mL) was treated with K_2_CO_3_ (2.7 mg, 19 μmol). The heterogeneous reaction mixture was stirred at room temperature for 40 min. The reaction was then quenched by the addition of acetic acid (3.0 μL, 52 μmol). The crude mixture was filtered through a silica pad (2 cm × 2 cm, methanol). The filtrate was concentrated and chromatographed [silica, CH_2_Cl_2_/methanol (9:1)] to afford a pale-yellow non-crystalline solid (10 mg, 84%): ^1^H NMR (500 MHz, CD_3_OD) *δ* 7.50 (s, 1H), 7.12 (s, 1H), 4.83 (d, *J* = 7.7 Hz, 1H), 4.15 (t, *J* = 4.9 Hz, 2H), 3.99–3.91 (m, 3H), 3.82–3.76 (m, 5H), 3.71–3.63 (m, 5H), 3.62–3.55 (m, 3H), 3.49 (dd, *J* = 9.1 Hz, 1H); ^13^C{^1^H} NMR (125 MHz, CD_3_OD) *δ* 171.1, 146.9, 138.5, 132.9, 120.0, 116.0, 115.4, 112.3, 107.7, 105.3, 77.3, 76.8, 74.9, 73.8, 73.6, 73.0, 71.8, 71.5, 71.3, 62.3, 52.9; ESI-MS obsd 625.9881, calcd 625.9878 [(M − H)^−^, M = C_21_H_27_Br_2_NO_11_].

*4,6-Dibromo-5-[1-hydroxy-3,6,9-trioxanon-9-yl]-1H-indol-3-yl β-D-glucopyranosiduronic acid* (**20**). A solution of **19** (10 mg, 16 μmol) in methanol (0.64 mL) was treated with aqueous NaHCO_3_ (100 mM, 0.16 mL) at room temperature and then stirred at 60 °C for 18 h. The reaction mixture was allowed to cool to room temperature and then concentrated. Chromatography [silica, CH_2_Cl_2_/methanol (4:1 to 1:4)] afforded a white non-crystalline solid (8.8 mg, 90%): ^1^H NMR (600 MHz, CD_3_OD) *δ* 7.49 (s, 1H), 7.35 (s, 1H), 4.73 (d, *J* = 7.7 Hz, 1H), 4.15 (t, *J* = 4.8 Hz, 2H), 3.95 (t, *J* = 4.8 Hz, 2H), 3.83–3.78 (m, 2H), 3.72–3.65 (m, 5H), 3.62–3.54 (m, 4H), 3.51 (t, *J* = 9.0 Hz, 1H); ^13^C{^1^H} NMR (150 MHz, CD_3_OD) *δ* 176.6, 146.7, 138.8, 132.8, 120.0, 116.2, 116.0, 112.0, 107.6, 105.1, 77.9, 76.6, 75.1, 73.71, 73.66, 73.6, 71.7, 71.5, 71.3, 62.2; ESI-MS obsd 611.9726, calcd 611.9722 [(M − H)^−^, M = C_20_H_25_Br_2_NO_11_].

*1-Acetyl-5-bromo-1H-indole-3-carbaldehyde* (**22-Br^5^**). A sample of DMAP (38 mg, 0.31 mmol) was added to a mixture of 5-bromo-3-formylindole (**21-Br^5^**, 7.0 g, 31 mmol), acetic anhydride (5.9 mL, 62 mmol), and triethylamine (8.5 mL, 61 mmol) in CH_2_Cl_2_ (78 mL) at room temperature. The reaction mixture was stirred for 18 h and then concentrated. The resulting crude product was washed with ice-cold CH_2_Cl_2_ (15 mL) to afford a bright yellow solid (7.4 g, 89%): mp 196–198 °C; ^1^H NMR (700 MHz, CDCl_3_) *δ* 10.10 (s, 1H), 8.45 (d, *J =* 2.0 Hz, 1H), 8.30 (d, *J =* 8.9 Hz, 1H), 8.06 (s, 1H), 7.55 (dd, *J =* 8.8, 2.1 Hz, 1H), 2.74 (s, 3H); ^13^C{^1^H} NMR (175 MHz, CDCl_3_) *δ* 185.1, 168.3, 135.5, 135.1, 129.9, 127.6, 124.7, 121.8, 119.1, 117.8, 23.8; ESI-MS obsd 265.9806, calcd 265.9811 [(M + H)^+^, M = C_11_H_8_BrNO_2_].

*1-Acetyl-6-bromo-1H-indole-3-carbaldehyde* (**22-Br^6^**). A sample of DMAP (38 mg, 0.31 mmol) was added to a mixture of 6-bromo-3-formylindole (**21-Br^6^**, 7.0 g, 31 mmol), acetic anhydride (5.9 mL, 62 mmol), and triethylamine (8.5 mL, 61 mmol) in CH_2_Cl_2_ (78 mL) at room temperature. The reaction mixture was stirred for 18 h and then concentrated. The resulting crude product was washed with ice-cold CH_2_Cl_2_ (30 mL) to afford a white solid (7.5 g, 90%): mp darkening at 214 °C, decomposed at 247 °C; ^1^H NMR (700 MHz, CDCl_3_) *δ* 10.11 (s, 1H), 8.65 (d, *J =* 1.7 Hz, 1H), 8.15 (d, *J =* 8.4 Hz, 1H), 8.04 (s, 1H), 7.54 (dd, *J =* 8.4, 1.8 Hz, 1H), 2.74 (s, 3H); ^13^C{^1^H} NMR (175 MHz, CDCl_3_) *δ* 185.2, 168.3, 136.9, 135.0, 128.8, 124.9, 123.0, 122.4, 120.7, 119.6, 23.8; ESI-MS obsd 265.9810, calcd 265.9811 [(M + H)^+^, M = C_11_H_8_BrNO_2_].

*1-Acetyl-7-bromo-1H-indole-3-carbaldehyde* (**22-Br^7^**). A sample of DMAP (16 mg, 0.13 mmol) was added to a mixture of 7-bromo-3-formylindole (**21-Br^7^**, 3.0 g, 13 mmol), acetic anhydride (1.3 mL, 14 mmol), and triethylamine (2.0 mL, 14 mmol) in CH_2_Cl_2_ (67 mL) at room temperature. The mixture was stirred for 4 h and then neutralized by the addition of saturated aqueous NaHCO_3_ (100 mL), followed by brine (100 mL). The organic layer was dried (Na_2_SO_4_) and filtered. The filtrate was concentrated and chromatographed [silica, ethyl acetate gradient 0–7% in CH_2_Cl_2_] to afford a white solid (2.1 g, 59%): mp 122–124 °C; ^1^H NMR (700 MHz, CDCl_3_) *δ* 10.10 (s, 1H), 8.30 (d, *J =* 7.8 Hz, 1H), 8.08 (s, 1H), 7.65 (d, *J =* 7.8 Hz, 1H), 7.29 (t, *J =* 7.8 Hz, 1H), 2.78 (s, 3H); ^13^C{^1^H} NMR (175 MHz, CDCl_3_) *δ* 185.0, 167.2, 137.0, 135.0, 131.8, 129.8, 126.6, 121.5, 121.3, 108.2, 25.3; ESI-MS obsd 265.9809, calcd 265.9811 [(M + H)^+^, M = C_11_H_8_BrNO_2_].

*1-Acetyl-5-bromoindolin-3-one* (**23-Br^5^**). Following a reported procedure [25] with modification, a sample of *m*CPBA (purified, 863 mg, 5.0 mmol) was added to a mixture of **22-Br^5^** (1.06 g, 4.0 mmol) in CH_2_Cl_2_ (19 mL) at 0 °C under argon. The reaction mixture was stirred at 0 °C for 30 min and then at room temperature for 20.5 h. Methanol (19 mL) and acetic acid (2 mL) were then added to the reaction mixture, followed by anhydrous sodium acetate (492 mg, 6.0 mmol). The mixture was stirred at room temperature for 2 h. The reaction mixture was then concentrated. The resulting residue was dissolved in CH_2_Cl_2_ (100 mL), neutralized by the addition of saturated aqueous NaHCO_3_ (300 mL), and washed with brine (150 mL). The organic layer was dried (Na_2_SO_4_) and filtered. The filtrate was concentrated and chromatographed [silica, ethyl acetate gradient 0–2% in CH_2_Cl_2_] to afford a white solid (695 mg, 68%): mp darkening at 111 °C, decomposed at 179 °C; ^1^H NMR (700 MHz, CDCl_3_) *δ* 8.48 (d, *J* = 8.9 Hz, 1H), 7.86 (d, *J* = 2.2 Hz, 1H), 7.74 (dd, *J* = 8.8, 2.2 Hz, 1H), 4.32 (s, 2H), 2.32 (s, 3H); ^13^C{^1^H} NMR (175 MHz, CDCl_3_) *δ* 193.1, 168.0, 152.5, 139.9, 126.5, 126.4, 120.2, 117.4, 56.3, 24.2; ESI-MS obsd 251.9672, calcd 251.9666 [(M − H)^−^, M = C_10_H_8_BrNO_2_].

*1-Acetyl-6-bromoindolin-3-one* (**23-Br^6^**). Following a reported procedure [25] with modification, a sample of *m*CPBA (purified, 630 mg, 3.7 mmol) was added to a mixture of **22-Br^6^** (750 mg, 2.8 mmol) in CH_2_Cl_2_ (60 mL) at 0 °C under argon. The reaction mixture was stirred at 0 °C for 30 min and then at room temperature for 20 h. The reaction mixture was then concentrated. The resulting residue was dissolved in acetic acid/CH_2_Cl_2_/methanol (80 mL, 1:9.5:9.5, *v*/*v*/*v*), followed by the addition of anhydrous sodium acetate (346 mg, 4.2 mmol). The mixture was stirred at room temperature for 3 h. The mixture was concentrated. The resulting residue was dissolved in CH_2_Cl_2_ (100 mL), neutralized by the addition of saturated aqueous NaHCO_3_ (300 mL), and washed with brine (150 mL). The organic layer was dried (Na_2_SO_4_) and filtered. The filtrate was concentrated and chromatographed [silica, ethyl acetate gradient 0–1% in CH_2_Cl_2_] to afford a white fluffy solid (245 mg, 34%): mp darkening at 160 °C, decomposed at 182 °C; ^1^H NMR (700 MHz, CDCl_3_) *δ* 8.82 (s, 1H), 7.60 (d, *J* = 8.2 Hz, 1H), 7.36 (dd, *J* = 8.2, 1.6 Hz, 1H), 4.30 (s, 2H), 2.32 (s, 3H); ^13^C{^1^H} NMR (175 MHz, CDCl_3_) *δ* 193.4, 168.1, 154.1, 132.8, 127.9, 124.6, 123.7, 121.9, 56.3, 24.2; ESI-MS obsd 251.9672, calcd 251.9666 [(M − H)^−^, M = C_10_H_8_BrNO_2_].

*1-Acetyl-7-bromoindolin-3-one* (**23-Br^7^**). Following a reported procedure [25] with modification, a sample of *m*CPBA (75% as obtained commercially, 1.10 g, 4.8 mmol) was added to a mixture of **22-Br^7^** (1.06 g, 4.0 mmol) in CH_2_Cl_2_ (10 mL) at 0 °C under argon. The reaction mixture was stirred at 0 °C for 30 min and then at room temperature for 18 h. CH_2_Cl_2_ (8 mL), methanol (18 mL), and acetic acid (2 mL) were then added, followed by anhydrous sodium acetate (492 mg, 6.0 mmol). The reaction mixture was stirred at room temperature for 1 h. The mixture was concentrated. The resulting residue was dissolved in CH_2_Cl_2_ (100 mL), neutralized by the addition of saturated aqueous NaHCO_3_ (300 mL), and washed with brine (150 mL). The organic layer was dried (Na_2_SO_4_) and filtered. The filtrate was concentrated and chromatographed [silica, ethyl acetate gradient 0–5% in CH_2_Cl_2_] to afford a white solid (459 mg, 45%): mp darkening at 96 °C, decomposed at 173 °C; ^1^H NMR (700 MHz, CDCl_3_) *δ* 8.45 (d, *J =* 8.9 Hz, 1H), 7.88 (d, *J =* 7.8 Hz, 1H), 7.73 (d, *J =* 7.5 Hz, 1H), 7.17 (t, *J =* 7.7 Hz, 1H), 4.37 (s, 2H), 2.36 (s, 3H); ^13^C{^1^H} NMR (175 MHz, CDCl_3_) *δ* 194.8, 167.9, 153.4, 141.7, 129.9, 126.4, 122.8, 113.8, 57.6, 24.3; ESI-MS obsd 251.9669, calcd 251.9666 [(M − H)^−^, M = C_10_H_8_BrNO_2_].

*1-Acetyl-5-bromo-1H-indol-3-yl 2,3,4-tri-O-acetyl-β-D-glucopyranosiduronic acid methyl ester* (**24-Br^5^**). Mercury(II) oxide (238 mg, 1.1 mmol) and mercury(II) bromide (72 mg, 0.20 mmol) were added simultaneously to a mixture of **23-Br^5^** (254 mg, 1.0 mmol), **6** (1.19 g, 3.0 mmol), and powdered molecular sieves 3Å (300 mg) in CH_2_Cl_2_ (5 mL) under argon at room temperature, followed by stirring in a water bath (35 °C) for 21 h. The reaction mixture was treated with pyridine (242 μL, 3.0 mmol) and filtered through Celite. The filtrate was concentrated and chromatographed [silica, ethyl acetate gradient 0–4% in CH_2_Cl_2_] to afford a white solid (215 mg, 38%): mp 175–178 °C; ^1^H NMR (500 MHz, CDCl_3_) *δ* 8.29 (s, 1H), 7.64 (d, *J* = 2.0 Hz, 1H), 7.46 (dd, *J* = 8.8, 2.0 Hz, 1H), 7.21 (s, 1H), 5.42–5.28 (m, 3H), 5.07 (d, *J* = 6.7 Hz, 1H), 4.19 (d, *J* = 9.2 Hz, 1H), 3.77 (s, 3H), 2.58 (s, 3H), 2.14 (s, 3H), 2.07 (s, 3H), 2.06 (s, 3H); ^13^C{^1^H} NMR (175 MHz, CDCl_3_) *δ* 170.1, 169.3, 169.2, 168.2, 166.7, 139.9, 132.2, 129.2, 125.6, 120.6, 118.2, 117.1, 111.7, 100.8, 72.7, 71.7, 70.8, 68.9, 53.1, 23.8, 20.7, 20.6, 20.5; ESI-MS obsd 592.0417, calcd 592.0425 [(M + Na)^+^, M = C_23_H_24_BrNO_11_].

*1-Acetyl-6-bromo-1H-indol-3-yl 2,3,4-tri-O-acetyl-β-D-glucopyranosiduronic acid methyl ester* (**24-Br^6^**). Mercury(II) oxide (206 mg, 0.95 mmol) and mercury(II) bromide (62 mg, 0.17 mmol) were added simultaneously to a mixture of **23-Br^6^** (220 mg, 866 μmol), **6** (1.03 g, 2.6 mmol), and powdered molecular sieves 3Å (250 mg) in CH_2_Cl_2_ (4.3 mL) under argon at room temperature, followed by stirring in a water bath (35 °C) for 21 h. The reaction mixture was treated with pyridine (209 μL, 2.6 mmol) and filtered through Celite. The filtrate was concentrated and chromatographed [silica, ethyl acetate gradient 0–1% in CH_2_Cl_2_] to afford a white solid (218 mg, 44%): mp 196–199 °C; ^1^H NMR (700 MHz, CDCl_3_) *δ* 8.63 (s, 1H), 7.41 (dd, *J* = 8.2, 1.7 Hz, 1H), 7.36 (d, *J* = 8.3 Hz, 1H), 7.16 (s, 1H), 5.40–5.31 (m, 3H), 5.09 (d, *J* = 6.8 Hz, 1H), 4.21 (d, *J* = 9.0 Hz, 1H), 3.75 (s, 3H), 2.58 (s, 3H), 2.11 (s, 3H), 2.07 (s, 3H), 2.06 (s, 3H); ^13^C{^1^H} NMR (175 MHz, CDCl_3_) *δ* 170.0, 169.3, 169.1, 168.2, 166.7, 140.8, 134.0, 127.1, 122.7, 120.2, 119.8, 118.8, 110.1, 100.7, 72.7, 71.6, 70.9, 68.9, 53.1, 23.8, 20.7, 20.6, 20.5; ESI-MS obsd 592.0415, calcd 592.0425 [(M + Na)^+^, M = C_23_H_24_BrNO_11_].

*1-Acetyl-7-bromo-1H-indol-3-yl 2,3,4-tri-O-acetyl-β-D-glucopyranosiduronic acid methyl ester* (**24-Br^7^**). Mercury(II) bromide (49 mg, 0.14 mmol) was added to a mixture of **23-Br^7^** (171 mg, 0.67 mmol), **6** (802 mg, 2.0 mmol), mercury(II) oxide (160 mg, 0.74 mmol), and powdered molecular sieves 3Å (150 mg) in CH_2_Cl_2_ (3 mL) with stirring under argon at room temperature, followed by stirring in a water bath (35 °C) for 18 h. The reaction mixture was treated with pyridine (162 μL, 2.0 mmol) and filtered through Celite. The filtrate was concentrated and chromatographed [silica, ethyl acetate gradient 0–6% in CH_2_Cl_2_] to afford a white solid (128 mg, 33%): mp 103–110 °C; ^1^H NMR (700 MHz, CDCl_3_) *δ* 7.59 (d, *J =* 7.7 Hz, 1H), 7.47 (d, *J =* 7.8 Hz, 1H), 7.22 (s, 1H), 7.16 (t, *J =* 7.7 Hz, 1H), 5.41–5.31 (m, 3H), 5.07 (d, *J =* 6.7 Hz, 1H), 4.20 (d, *J =* 9.2 Hz, 1H), 3.76 (s, 3H), 2.62 (s, 3H), 2.11 (s, 3H), 2.07 (s, 3H), 2.06 (s, 3H); ^13^C{^1^H} NMR (175 MHz, CDCl_3_) *δ* 170.1, 169.3, 169.1, 166.8, 166.7, 139.8, 132.8, 131.5, 128.0, 124.9, 117.0, 112.4, 109.5, 100.8, 72.7, 71.7, 70.9, 68.9, 53.1, 24.8, 20.7, 20.6, 20.5; ESI-MS obsd 592.0418, calcd 592.0425 [(M + Na)^+^, M = C_23_H_24_BrNO_11_].

*5-(2,5,8,11-Tetraoxatetradec-13-yn-14-yl)-1H-indol-3-yl β-D-glucopyranosiduronic acid* (**25**). A mixture of **24-Br^5^** (120 mg, 0.21 mmol), propargyl-PEG_3_-OMe (**P4**, 128 mg, 0.63 mmol), and triethylamine/THF (1:5, 1 mL) was degassed via two cycles of freeze-pump-thaw under argon. The degassed reaction mixture was treated simultaneously with Pd(PPh_3_)_4_ (24 mg, 21 μmol) and CuI (8.0 mg, 42 μmol) at room temperature under argon and then stirred at 60 °C for 21 h. The reaction mixture was concentrated. The resulting residue was dissolved in CH_2_Cl_2_ (2 mL) and filtered through Celite. The filtrate was concentrated and chromatographed [silica, CH_2_Cl_2_/ethyl acetate (9:1 to 1:1)] to afford a crude mixture (21 mg). The crude mixture was added to a mixture of K_2_CO_3_ (6.3 mg, 46 μmol) and methanol (1.5 mL) and stirred at room temperature for 4 h, followed by filtration through Celite. The filtrate was concentrated and chromatographed on C_18_-reversed phase silica (4 g, column volume = 8 mL, flow rate = 15 mL/min) with the following eluants: H_2_O with a gradient of 0–8% acetonitrile over a period of 21 min, hold at 8% acetonitrile until 37 min, then increase abruptly to 12% acetonitrile, and hold until ≥41 min. The title compound eluted at 27.1 min, whereas the byproduct **25-elim** eluted at 41.0 min. Data for the title compound: a pale yellow oil (8.2 mg, 8%); ^1^H NMR (700 MHz, CD_3_OD) *δ* 7.85 (s, 1H), 7.23 (d, *J* = 8.4 Hz, 1H), 7.22 (s, 1H), 7.14 (d, *J* = 8.4 Hz, 1H), 4.69 (d, *J* = 7.7 Hz, 1H), 4.43 (s, 2H), 3.79–3.76 (m, 2H), 3.71–3.68 (m, 2H), 3.66–3.60 (m, 7H), 3.56–3.47 (m, 5H), 3.34 (s, 3H); ^13^C{^1^H} NMR (175 MHz, CD_3_OD) *δ* 176.6, 138.9, 134.8, 126.2, 123.0, 121.3, 114.2, 113.5, 112.5, 105.8, 89.4, 82.8, 77.9, 76.6, 74.9, 73.7, 72.8, 71.33, 71.30, 71.27, 71.2, 69.9, 60.0, 59.1; ESI-MS obsd 508.1832, calcd 508.1824 [(M − H)^−^, M = C_24_H_31_NO_11_]. Data for **25-elim**: ESI-MS obsd 490.1717, calcd 490.1719 [(M − H)^−^, M = C_24_H_29_NO_10_].

*6-(2,5,8,11-Tetraoxatetradec-13-yn-14-yl)-1H-indol-3-yl β-D-glucopyranosiduronic acid* (**26**). A mixture of **24-Br^6^** (120 mg, 0.21 mmol), propargyl-PEG_3_-OMe (**P4**, 128 mg, 0.63 mmol), and triethylamine/THF (1:5, 1 mL) was degassed via three cycles of freeze-pump-thaw under argon. The degassed reaction mixture was treated simultaneously with Pd(PPh_3_)_4_ (24 mg, 21 μmol) and CuI (8.0 mg, 42 μmol) at room temperature under argon and then stirred at 60 °C for 21 h. The reaction mixture was concentrated. The resulting residue was dissolved in CH_2_Cl_2_ (2 mL) and filtered through Celite. The filtrate was concentrated and chromatographed [silica, CH_2_Cl_2_/ethyl acetate (9:1 to 1:1)] to afford a crude mixture (41 mg). The crude mixture was added to a mixture of K_2_CO_3_ (12.3 mg, 89 μmol) and methanol (3 mL) and stirred at room temperature for 4 h, followed by filtration through Celite. The filtrate was then concentrated and chromatographed on C_18_-reversed phase silica (4 g, column volume = 8 mL, flow rate = 15 mL/min) with the following eluants: H_2_O with a gradient of 0–7% in acetonitrile over a period of 8 min, then hold at 7% acetonitrile until ≥ 12 min. The title compound eluted at 10.0 min, whereas the byproduct **26-elim** eluted at 11.5 min. Data for the title compound: a pale yellow oil (25 mg, 23%); ^1^H NMR (700 MHz, CD_3_OD) *δ* 7.66 (d, *J =* 8.2 Hz, 1H), 7.38 (s, 1H), 7.24 (s, 1H), 7.03 (d, *J =* 8.2 Hz, 1H), 4.70 (d, *J =* 7.6 Hz, 1H), 4.43 (s, 2H), 3.78–3.74 (m, 2H), 3.72–3.66 (m, 3H), 3.66–3.60 (m, 6H), 3.59–3.47 (m, 5H), 3.33 (s, 3H); ^13^C{^1^H} NMR (175 MHz, CD_3_OD) *δ* 176.6, 139.1, 134.5, 123.1, 121.6, 118.8, 116.5, 116.1, 115.0, 105.8, 89.2, 83.8, 77.9, 76.6, 74.9, 73.7, 72.8, 71.37, 71.35, 71.3, 71.2, 70.0, 59.9, 59.1; ESI-MS obsd 508.1832, calcd 508.1824 [(M − H)^−^, M = C_24_H_31_NO_11_].

*7-(2,5,8,11-Tetraoxatetradec-13-yn-14-yl)-1H-indol-3-yl β-D-glucopyranosiduronic acid* (**27**). A mixture of **24-Br^7^** (120 mg, 0.21 mmol), propargyl-PEG_3_-OMe (**P4**, 128 mg, 0.63 mmol), and triethylamine/THF (1:5, 1 mL) was degassed via three cycles of freeze-pump-thaw under argon. The degassed reaction mixture was treated simultaneously with Pd(PPh_3_)_4_ (24 mg, 21 μmol) and CuI (8.0 mg, 42 μmol) at room temperature under argon and then stirred at 60 °C for 21 h. The reaction mixture was concentrated. The resulting residue was dissolved in CH_2_Cl_2_ (2 mL) and filtered through Celite. The filtrate was concentrated and chromatographed [silica, CH_2_Cl_2_/ethyl acetate (9:1 to 1:1)] to afford a crude mixture (65 mg). The crude mixture was added to a mixture of K_2_CO_3_ (20 mg, 0.14 mmol) and methanol (4.7 mL) and stirred at room temperature for 4 h, followed by filtration through Celite. The filtrate was concentrated and chromatographed on C_18_-reversed phase silica (4 g, column volume = 8 mL, flow rate = 15 mL/min) with the following eluants: H_2_O with a gradient of 0–10% acetonitrile over a period of 18 min, hold at 10% acetonitrile until 24.2 min, then increase to 13% acetonitrile over a period of 4 min, then hold at 13% acetonitrile until ≥31 min. The title compound eluted at 20.1 min, whereas the byproduct **27-elim** eluted at 31 min. Data for the title compound: a pale yellow oil (32 mg, 30%), ^1^H NMR (700 MHz, CD_3_OD) *δ* 7.75 (d, *J* = 8.0 Hz, 1H), 7.23 (s, 1H), 7.19 (d, *J* = 7.2 Hz, 1H), 6.96 (t, *J* = 7.6 Hz, 1H), 4.70 (d, *J* = 7.7 Hz, 1H), 4.53 (s, 2H), 3.83–3.79 (m, 2H), 3.73–3.69 (m, 2H), 3.67–3.64 (m, 3H), 3.64–3.61 (m, 2H), 3.61–3.58 (m, 2H), 3.57–3.52 (m, 2H), 3.51–3.46 (m, 3H), 3.32 (s, 3H); ^13^C{^1^H} NMR (175 MHz, CD_3_OD) *δ* 176.6, 139.4, 135.4, 126.6, 121.6, 120.1, 119.4, 113.5, 106.7, 105.8, 89.8, 83.7, 77.9, 76.7, 74.9, 73.7, 72.8, 71.4, 71.3, 71.2, 70.2, 60.0, 59.1 (one expected carbon is missing); ESI-MS obsd 508.1829, calcd 508.1824 [(M − H)^−^, M = C_24_H_31_NO_11_].

### 3.3. Protocols for Enzymatic Tests (Table 1)

#### 3.3.1. Reactions with β-Glucuronidase from Bovine Liver

A solution of an indoxyl-glucuronide in DMSO (1 μL, 10 mM) and a solution of β-glucuronidase from bovine liver in H_2_O (5 μL, 800 U/mL) were mixed with 50 mM sodium acetate buffer (94 μL, pH 5.0). The resulting concentrations were indoxyl-glucuronide (100 μM) and β-glucuronidase (40 U/mL) in a medium containing 1% DMSO. The reaction mixture was incubated at 37 °C for 24 h and then centrifuged for 15 min. Any precipitate was separated from the supernatant and dissolved in DMF/H_2_O (*v*/*v* = 2:1, 100 μL) or DMF (100 μL). The resulting solution was analyzed by absorption spectroscopy.

#### 3.3.2. Reactions with β-Glucuronidase from *E. coli*

A solution of an indoxyl-glucuronide in DMSO (1 μL, 10 mM) and a solution of β-glucuronidase from *E. coli* in H_2_O (5 μL, 800 U/mL) were mixed with 50 mM sodium phosphate buffer (94 μL, pH 7.0). The resulting concentrations were indoxyl-glucuronide (100 μM) and β-glucuronidase (40 U/mL) in a medium containing 1% DMSO. The reaction mixture was incubated at 37 °C for 24 h and then centrifuged for 15 min. Any precipitate was separated from the supernatant and dissolved in DMF/H_2_O (*v*/*v* = 2:1, 100 μL) or DMF (100 μL). The resulting solution was analyzed by absorption spectroscopy.

#### 3.3.3. Reactions with Rat Liver Tritosomes

A solution of an indoxyl-glucuronide in DMSO (1 μL, 10 mM) and a solution of rat liver tritosomes in H_2_O (50 μL, 0.25 mg protein/mL) were mixed with 10X catabolic buffer (10 μL, pH 4.9) and H_2_O (39 μL). The resulting concentrations were indoxyl-glucuronide (100 μM) in a medium containing 1% DMSO. The reaction mixture was incubated at 37 °C for 24 h and then centrifuged for 15 min. Any precipitate was separated from the supernatant and dissolved in DMF/H_2_O (*v*/*v* = 2:1, 100 μL) or DMF (100 μL). The resulting solution was analyzed by absorption spectroscopy.

### 3.4. Preparation and Characterization of Indigoids

#### 3.4.1. Preparation of **Ind(25)_2_**

A solution of **25** in DMSO (450 μL, 10 mM) and a solution of β-glucuronidase from *E. coli* in H_2_O (257 μL, 7.0 kU/mL) were mixed with 50 mM sodium phosphate buffer (pH 7.0). The total reaction volume was 45 mL. The substrate and enzyme concentrations in the mixture were 100 μM and 40 U/mL. The reaction mixture was incubated at 37 °C for 24 h and then centrifuged for 15 min. Any precipitate was separated from the supernatant and then washed with deionized water (15 mL × 3). The resulting product was freeze-dried overnight to afford an indigo-blue solid (1.38 mg, 93%): ^1^H NMR (700 MHz, CDCl_3_) *δ* 8.99 (s, 2H), 7.84–7.81 (m, 2H), 7.56 (dd, *J* = 8.4, 1.7 Hz, 2H), 7.00 (d, *J* = 8.3 Hz, 2H), 4.43 (s, 4H), 3.78–3.76 (m, 4H), 3.73–3.71 (m, 4H), 3.71–3.67 (m, 8H), 3.67–3.64 (m, 4H), 3.57–3.54 (m, 4H), 3.38 (s, 6H); ^13^C{^1^H} NMR (175 MHz, CDCl_3_) *δ* 187.8, 151.1, 139.4, 128.2, 121.5, 119.9, 115.5, 112.3, 85.4, 84.7, 72.0, 70.7, 70.62, 70.55, 70.5, 69.2, 59.2, 59.1; ESI-MS obsd 661.2777, calcd 661.2767 [(M − H)^−^, M = C_36_H_42_N_2_O_10_].

#### 3.4.2. Preparation of **Ind(26)_2_**

A solution of **26** in DMSO (350 μL, 10 mM) and a solution of β-glucuronidase from *E. coli* in H_2_O (200 μL, 7.0 kU/mL) were mixed with 50 mM sodium phosphate buffer (pH 7.0). The total reaction volume was 35 mL. The substrate and enzyme concentrations in the mixture were 100 μM and 40 U/mL. The reaction mixture was incubated at 37 °C for 24 h and then centrifuged for 15 min. Any precipitate was separated from the supernatant and then washed with deionized water (15 mL × 3). The resulting product was freeze-dried overnight to afford an indigo-blue solid (1.09 mg, 94%): ^1^H NMR (700 MHz, CDCl_3_) *δ* 8.97 (s, 1H), 7.68 (d, *J* = 7.9 Hz, 1H), 7.12 (s, 1H), 7.04 (dd, *J* = 7.9, 1.2 Hz, 1H), 4.46 (s, 2H), 3.80–3.77 (m, 2H), 3.74–3.72 (m, 2H), 3.71–3.68 (m, 4H), 3.67–3.65 (m, 2H), 3.58–3.55 (m, 2H), 3.39 (s, 3H); ^13^C{^1^H} NMR (175 MHz, CDCl_3_) *δ* 187.7, 151.3, 130.3, 124.44, 124.37, 121.9, 119.6, 115.3, 89.5, 86.0, 72.0, 70.7, 70.64, 70.56, 70.5, 69.5, 59.2, 59.1; ESI-MS obsd 661.2785, calcd 661.2767 [(M − H)^−^, M = C_36_H_42_N_2_O_10_].

#### 3.4.3. Preparation of **Ind(27)_2_**

A solution of **27** in DMSO (450 μL, 10 mM) and a solution of β-glucuronidase from *E. coli* in H_2_O (430 μL, 4.2 kU/mL) were mixed with 50 mM sodium phosphate buffer (pH 7.0). The total reaction volume was 45 mL. The substrate and enzyme concentrations in the mixture were 100 μM and 40 U/mL. The reaction mixture was incubated at 37 °C for 24 h and then centrifuged for 15 min. Any precipitate was separated from the supernatant and then washed with deionized water (15 mL × 3). The resulting product was freeze-dried overnight to afford an indigo-blue solid (1.34 mg, 90%): ^1^H NMR (700 MHz, CDCl_3_) *δ* 9.12 (s, 2H), 7.70 (dd, *J* = 7.6, 1.2 Hz, 2H), 7.57 (dd, *J* = 7.5, 1.1 Hz, 2H), 6.97 (t, *J* = 7.6 Hz, 2H), 4.56 (s, 4H), 3.88–3.84 (m, 4H), 3.80–3.77 (m, 4H), 3.74–3.71 (m, 4H), 3.71–3.68 (m, 4H), 3.67–3.64 (m, 4H), 3.57–3.54 (m, 4H), 3.37 (s, 6H); ^13^C{^1^H} NMR (175 MHz, CDCl_3_) *δ* 188.2, 152.6, 138.6, 124.7, 121.2, 120.8, 119.9, 107.1, 92.0, 79.9, 72.0, 70.7, 70.63, 70.55, 70.5, 69.5, 59.2, 59.0; ESI-MS obsd 663.2910, calcd 663.2912 [(M + H)^+^, M = C_36_H_42_N_2_O_10_].

#### 3.4.4. Determination of Molar Absorption Coefficient (ε)

Each indigoid derivative, **Ind(25)_2_** (1.38 mg), **Ind(26)_2_** (1.09 mg), or **Ind(27)_2_** (1.34 mg), was dissolved in DMF to prepare a 2 mM stock solution. The stock solution was further diluted with DMF to prepare a 5, 10, 20, 30, 40, or 50 μM solution. The absorbance of each solution was recorded. A plot showing absorbance as a function of indigoid concentration was generated. The molar absorption coefficient was then determined by the slope.

## 4. Outlook

The synthesis of an indoxyl-glucuronide from an indoxyl-glucoside has been demonstrated in the case where the indole nitrogen and three secondary hydroxy groups of the glucoside are protected with acetyl groups. Conditions have been deployed for selective cleavage of the *N*-acetyl group in the presence of *O*-acetyl groups and the methyl ester, for selective cleavage of the *O*-acetyl groups in the presence of the methyl ester, and for saponification of the glucuronide methyl ester in the presence of a variety of functional groups, including hydroxy (PEG termini and glucosyl), azide, alkyne, carbamate, and the unprotected indole. The placement of the PEG-ethynyl group at the 6-position provides yields of indigoid dye comparable to those of the 4,6-dibromo-5-alkoxy substitution pattern upon treatment with β-glucuronidase at neutral conditions. The indigoid yield from the 6-PEGylated indoxyl-glucuronide (**26**) was 3-fold higher than that of the 5- and 7-positional isomers (**25** and **27**) under acidic conditions. Conversion of indoxyl-glucuronides to the corresponding indigoid dye has an absolute requirement for full display of unprotected hydroxy and carboxylic acid groups on the glucuronide moiety. The indoxyl-glucuronides bearing functional tethers described herein (**10**, **12**, **17**, and **20**), with further development, may support applications in the life sciences that require cross-linking under physiological conditions.

## Data Availability

All data are contained within the paper and Supplementary Materials.

## Supplementary Materials

The following can be downloaded at: https://www.mdpi.com/article/10.3390/molecules28104143/s1, time course delineation; cocktail enzymatic test results; NMR spectral data for all new compounds.

## Sample Availability

Samples have generally been consumed during the course of research.
