# Supplementary material for: Tethered Indoxyl-Glucuronides for Enzymatically Triggered Cross-Linking
Source: Molecules. 2023 May 17;28(10):4143. doi: 10.3390/molecules28104143 (PMC10223988; doi:10.3390/molecules28104143)

Supporting Information for:

Tethered Indoxyl-Glucuronides for Enzymatically Triggered Cross-linking

Juno Son,[a] Zhiyuan Wu,[a] Jinghuai Dou, Hikaru Fujita, Phuong-Lien Doan Cao, Qihui Liu, and Jonathan S. Lindsey*

[a]Equal contributions by both authors

Department of Chemistry, North Carolina State University, Raleigh, North Carolina 27695-8204

Table of contents

Topic	Pages
(1) Time course enzymatic indigogenic reaction	S2
(2) Cocktail enzyme experiment	S4
(3) Identification of **25-elim**	S6
(4) NMR spectral data	S9

(1) Time course of enzymatic indigogenic reaction.

The indigogenic reactions of **20** were performed with two different β-glucuronidase (different optimal pH) utilizing various enzyme concentrations. A mixture of **20** (100 μM) and β-glucuronidase (10 U/mL, 40 U/mL or 160 U/mL) was incubated at 37 °C for a certain period. After incubation, the blue precipitate was separated from the supernatant through centrifugation and then subjected to dissolution. The resulting solution was analyzed by absorption spectroscopy. The yield of indigoid was estimated and is shown in Figure S1. Under both acidic (pH 5.0) and neutral (pH 7.0) conditions, 40 U/mL enzyme concentration is enough for the indigogenic reactions. The half time under acidic condition was about 5 h. The half time under neutral condition was less than 30 min. Hence, all the indigogenic reactions were incubated for 24 h thereafter to achieve fully conversion.

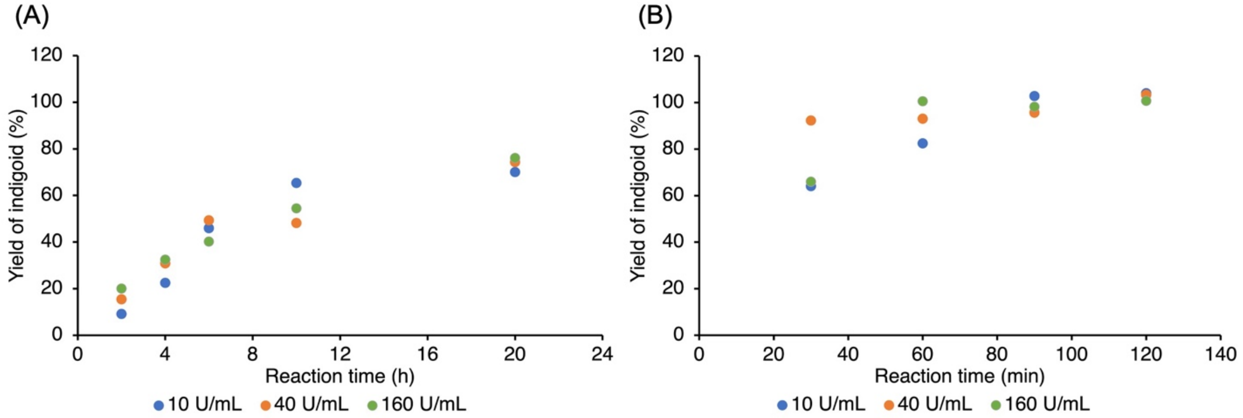

Figure S1. Time course of indigoid formation from **20** with β-glucuronidase from bovine liver (panel A, pH 5.0) or β-glucuronidase from *E. coli* (panel B, pH 7.0) with various enzyme concentrations.

The time course of indigoid formation from **12** was examined with the three conditions listed in Table 1. Under neutral conditions, a weak blue color was observed after 10 min at 37 °C whereas under both acidic conditions (treated with β-glucuronidase from bovine liver and rat liver tritosomes), a weak blue color was detected after about 2 h incubation. After 24 h incubation at 37 °C, a blue precipitate was formed under all three conditions. Centrifugation afforded the supernatant (colorless or slightly yellow) and a blue precipitate. The precipitates were dissolved in DMF/H_2O (2:1, 100 μL) to afford solutions that were used to collect absorption spectra. The full set of visible color changes are shown in Figure S2. Absorption spectroscopy analysis of the dissolved precipitates after 24 h of reaction are shown in Figure S3. Each spectrum shows the characteristic peak near 636 nm.

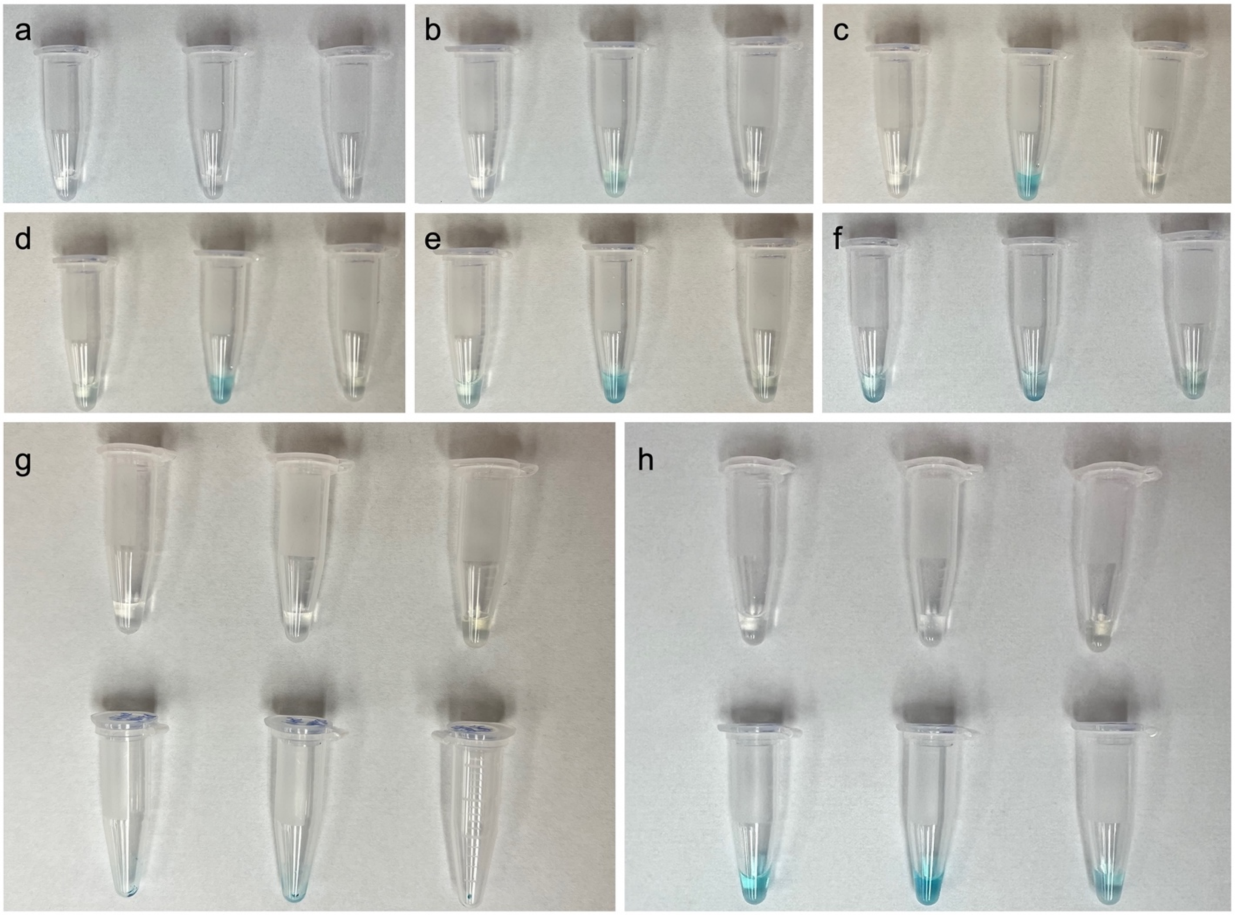

Figure S2. Time course of *in vitro* polymerization with **12** under three test conditions listed in Table 1 (from left to right: β-glucuronidase from bovine liver (pH 5.0); β-glucuronidase from *E. coli* (pH 7.0); rat liver tritosomes (pH 4.9)). a. 0 min; b. 10 min; c. 30 min; d. 2 h; e. 3 h; f. 24 h; g. 24 h followed with centrifugation (top: supernatant; bottom: precipitate); h. 24 h (top: supernatant; bottom: precipitate dissolved in DMF/H_2O (2:1, 100 μL)).

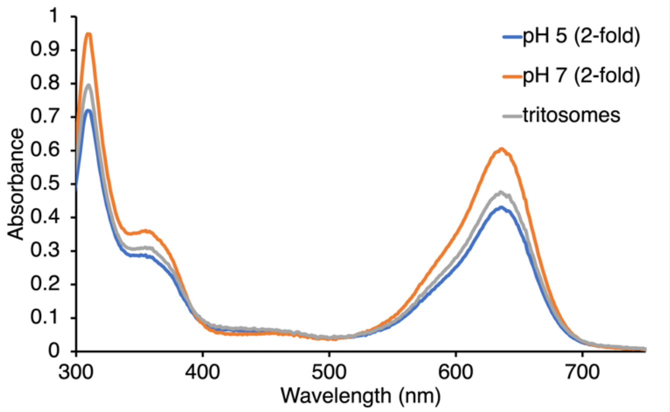

Figure S3. Absorption spectra (DMF/H_2O (2:1), room temperature) of indigoids formed with **12** under three test conditions. The spectra at pH 5 and pH 7 are diluted 2-fold.

(2) Cocktail enzyme experiment

Table S1. Enzymatic test with the presence of an esterase.

Compound	rat liver tritosomes (pH 4.9)	esterase from porcine liver (pH 7.0)	β-glucuronidase from bovine liver (pH 5.0)	β-glucuronidase from bovine liver + esterase (pH 5.0)	β-glucuronidase from *E. coli* (pH 7.0)	β-glucuronidase from *E. coli* + esterase (pH 7.0)
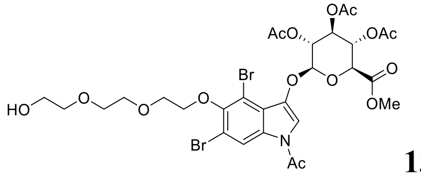 **13**	<1%[a]	<1%	–[b]	–	<1%	<1%
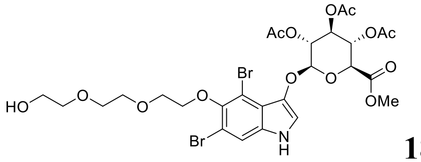 **18**	<1%	<1%	–	–	<1%	<1%
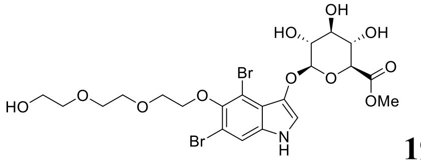 **19**	<1%	<1%	<1%	<1%	18±2%	29±1%
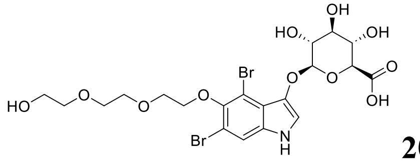 **20**	87%	<1%	53±1%	90±10%	109±3%	94±4%

[a] A yield of <1% indicates no blue color was observed by visual inspection and no absorption peak was observed spectroscopically.

[b] Not performed.

[c] All the reactions were incubated at 37 °C for 24 h.

Reactions with esterase from porcine liver. A solution of sample in DMSO (1 μL, 10 mM) and a solution of esterase from porcine liver in H_2O (6 μL, 180 U/mL) were mixed with 50 mM sodium phosphate buffer (93 μL, pH 7.0). The reaction mixture was incubated at 37 °C for 24 h and then centrifuged for 15 min. Any precipitate was separated from the supernatant and dissolved in DMF/H_2O (v/v=2:1, 100 μL). The resulting solution was analyzed by absorption spectroscopy. If there was no precipitate observed, the reaction mixture was analyzed by absorption spectroscopy directly.

Reactions with mixture of β-glucuronidase from bovine liver and esterase from porcine liver. A solution of sample in DMSO (1 μL, 10 mM), a solution of β-glucuronidase from bovine liver in H_2O (5 μL, 800 U/mL) and a solution of esterase from porcine liver in H_2O (6 μL, 180 U/mL) were mixed with 50 mM sodium acetate buffer (88 μL, pH 5.0). The reaction mixture was incubated at 37 °C for 24 h and then centrifuged for 15 min. Any precipitate was separated from the supernatant and dissolved in DMF/H_2O (v/v=2:1, 100 μL). The resulting solution was analyzed by absorption spectroscopy. If there was no precipitate observed, the reaction mixture was analyzed by absorption spectroscopy directly.

Reactions with mixture of β-glucuronidase from *E. coli* and esterase from porcine liver. A solution of sample in DMSO (1 μL, 10 mM), a solution of β-glucuronidase from *E. coli* in H_2O (5 μL, 800 U/mL) and a solution of esterase from porcine liver in H_2O (6 μL, 180 U/mL) were mixed with 50 mM sodium phosphate buffer (88 μL, pH 7.0). The reaction mixture was incubated at 37 °C for 24 h and then centrifuged for 15 min. Any precipitate was separated from the supernatant and dissolved in DMF/H_2O (v/v=2:1, 100 μL). The resulting solution was analyzed by absorption spectroscopy. If there was no precipitate observed, the reaction mixture was analyzed by absorption spectroscopy directly.

(3) Identification of 25-elim

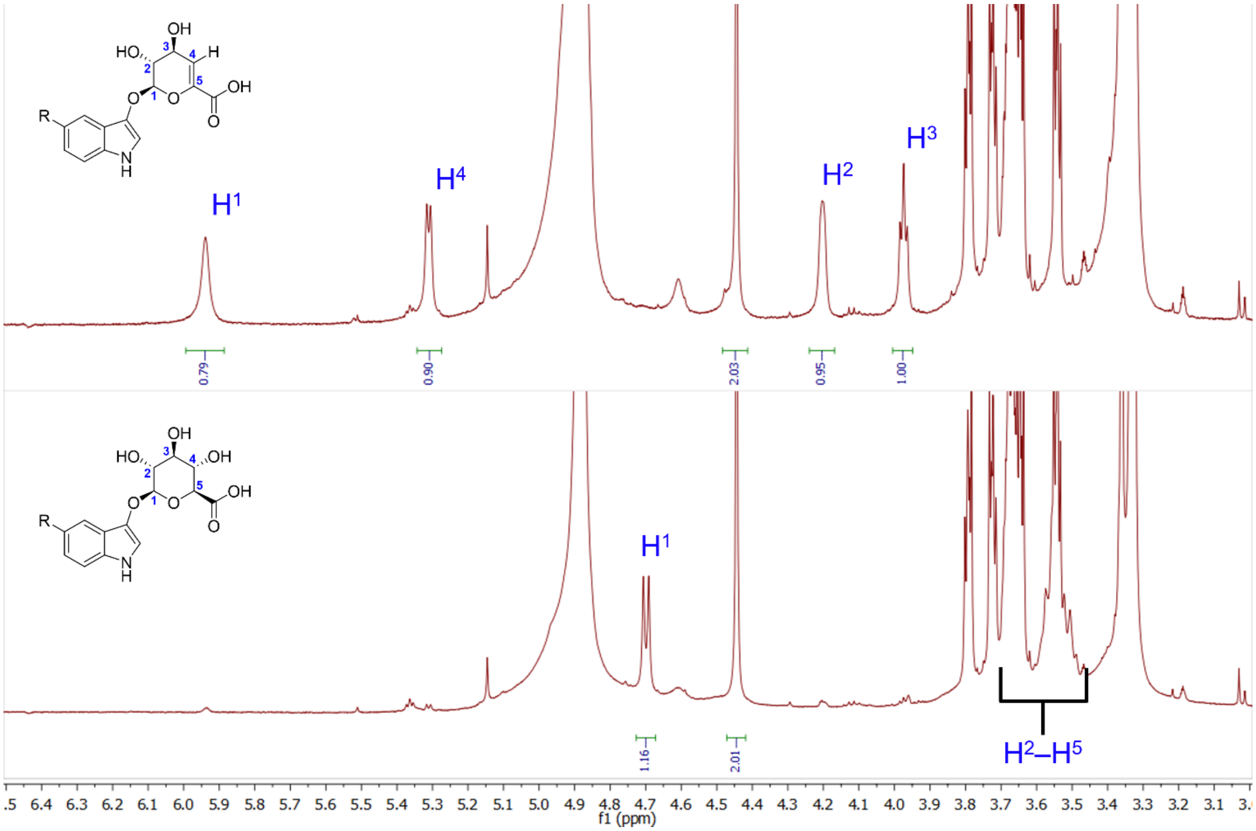

Figure S4. ^{1}H-NMR spectra of **25** (bottom) and **25-elim** (top).

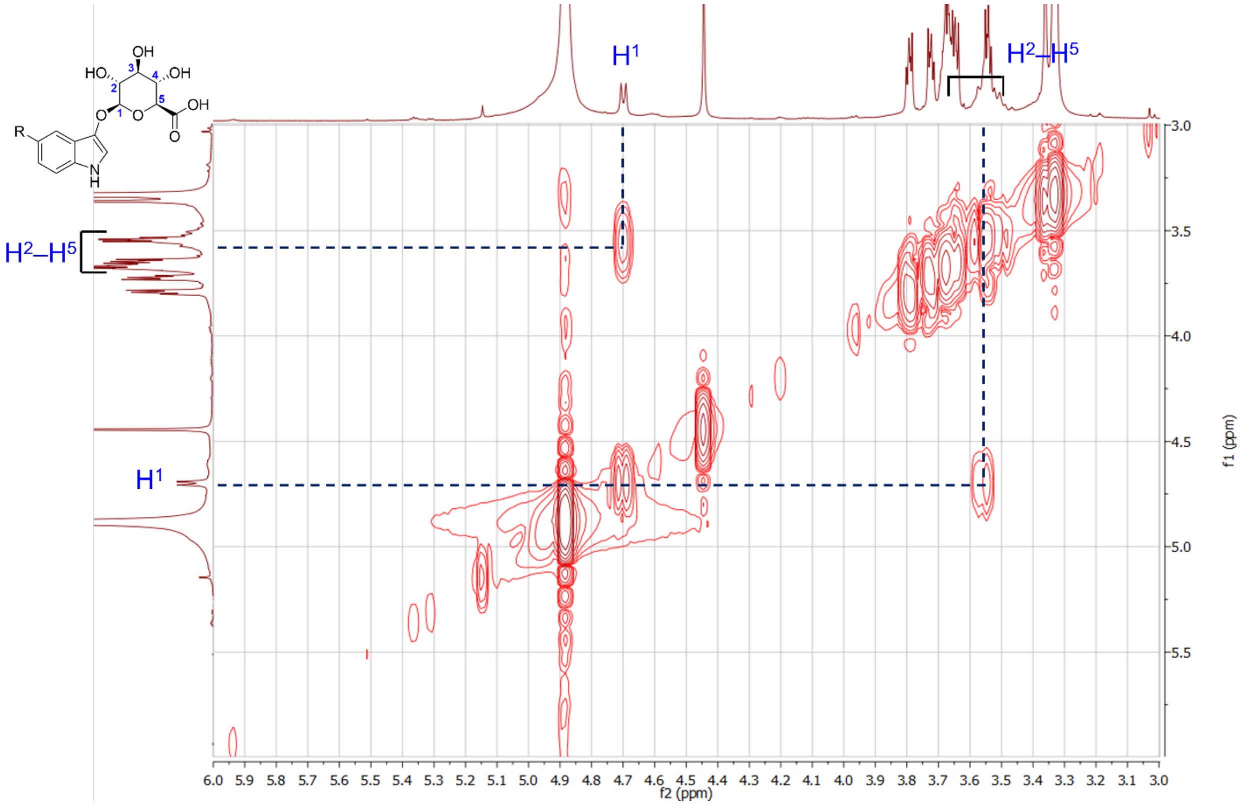

Figure S5. COSY-NMR spectra of **25**.

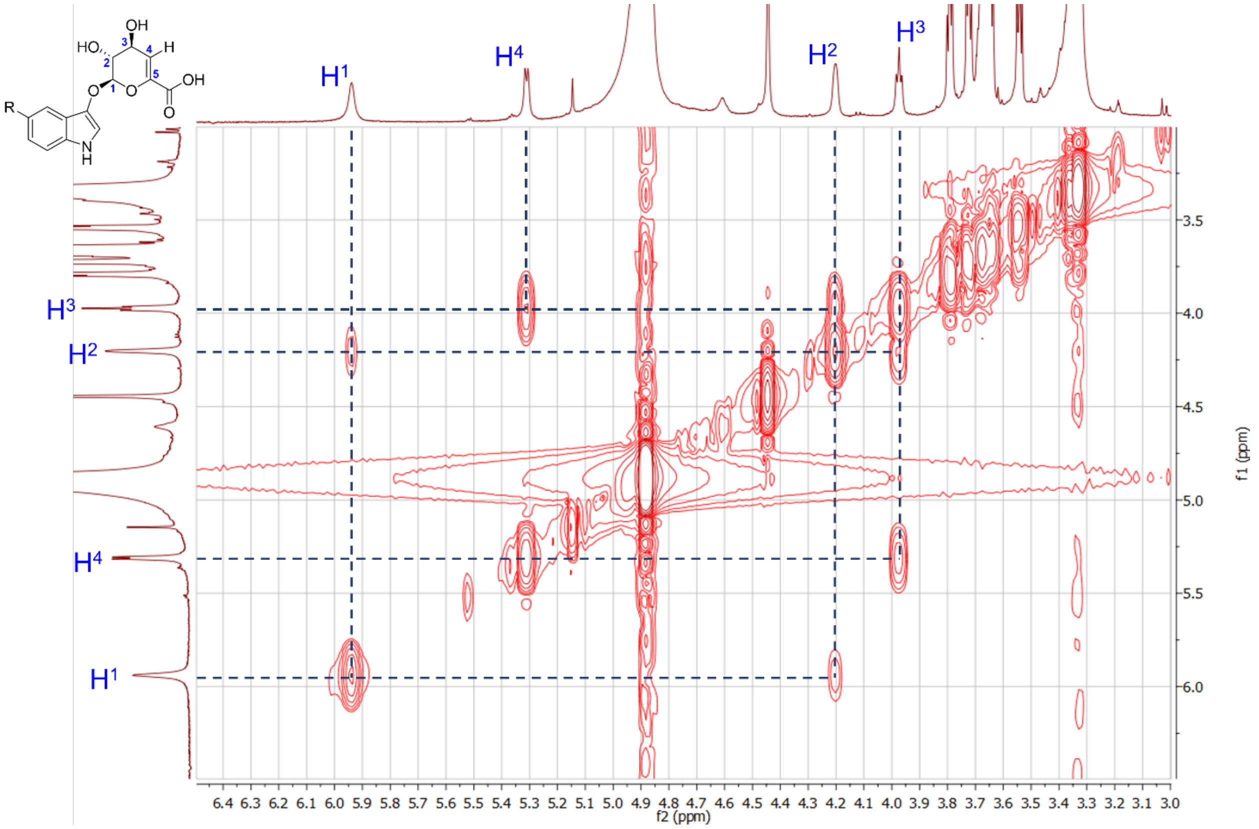

Figure S6. COSY-NMR spectra of **25-elim**.

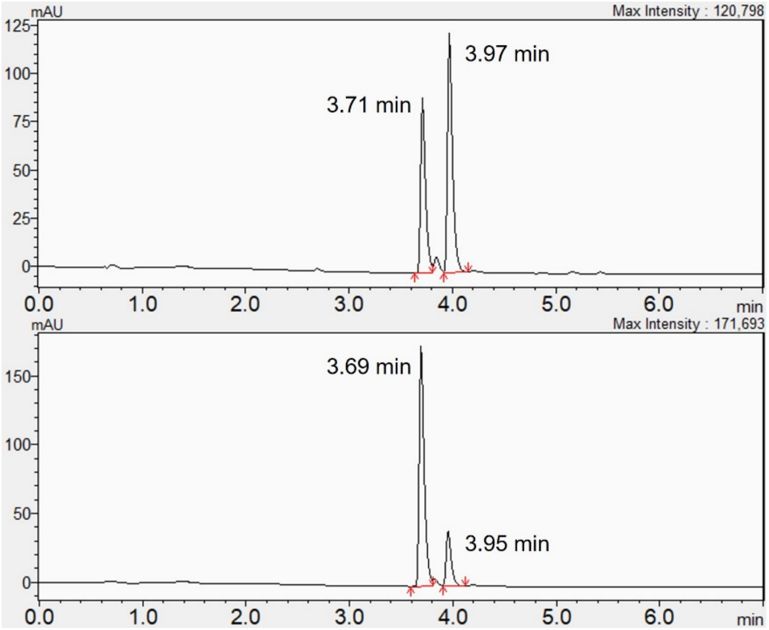

Compound	Retention time (min)	Area	Area %
**27**	3.71	292516	42
**27-elim**	3.97	409831	58

Compound	Retention time (min)	Area	Area %
**27**	3.69	581470	81
**27-elim**	3.95	136823	19

Figure S7. Liquid chromatogram (λ = 310 nm) for comparison of the crude mixture of **27** and **27-elim** after Sonogashira coupling of **24-Br**[7] at two different reaction temperatures (top panel 80 °C, bottom panel 60 °C).

(4) NMR spectral data

2, ¹H NMR (700 MHz, CD_3OD)

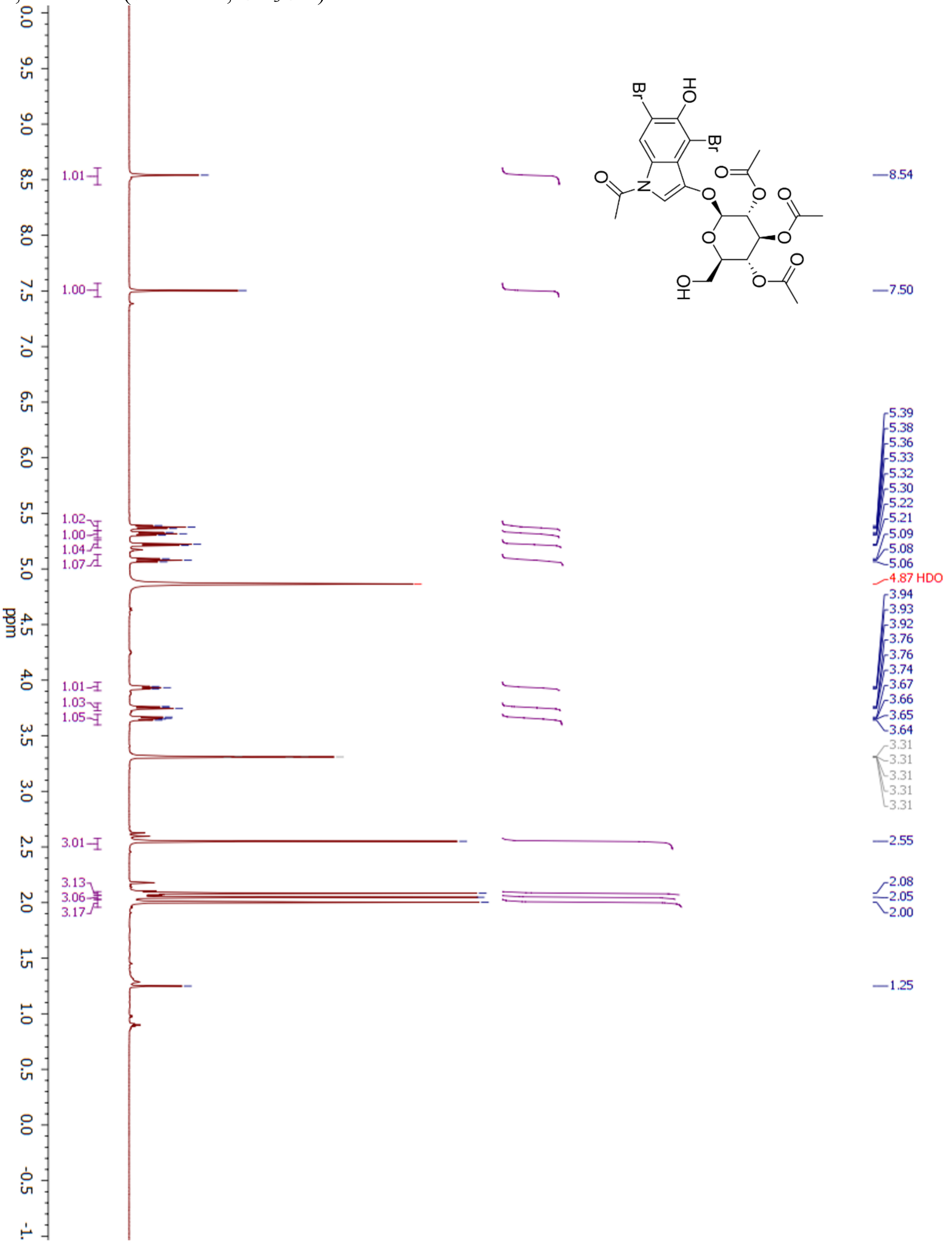

2, ^{13}C NMR (175 MHz, CD_3OD)

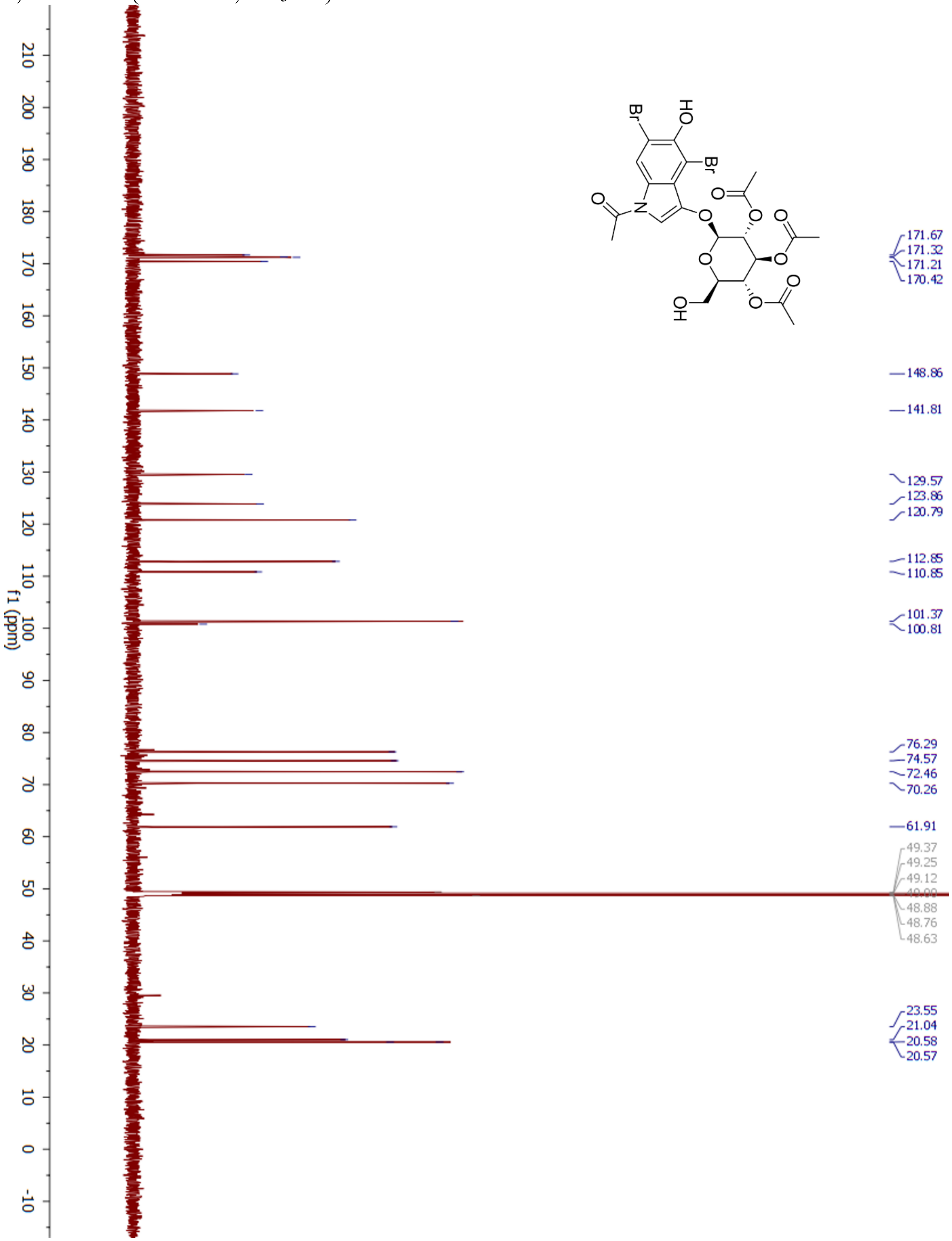

3, ^{1}H NMR (700 MHz, $CDCl_3$)

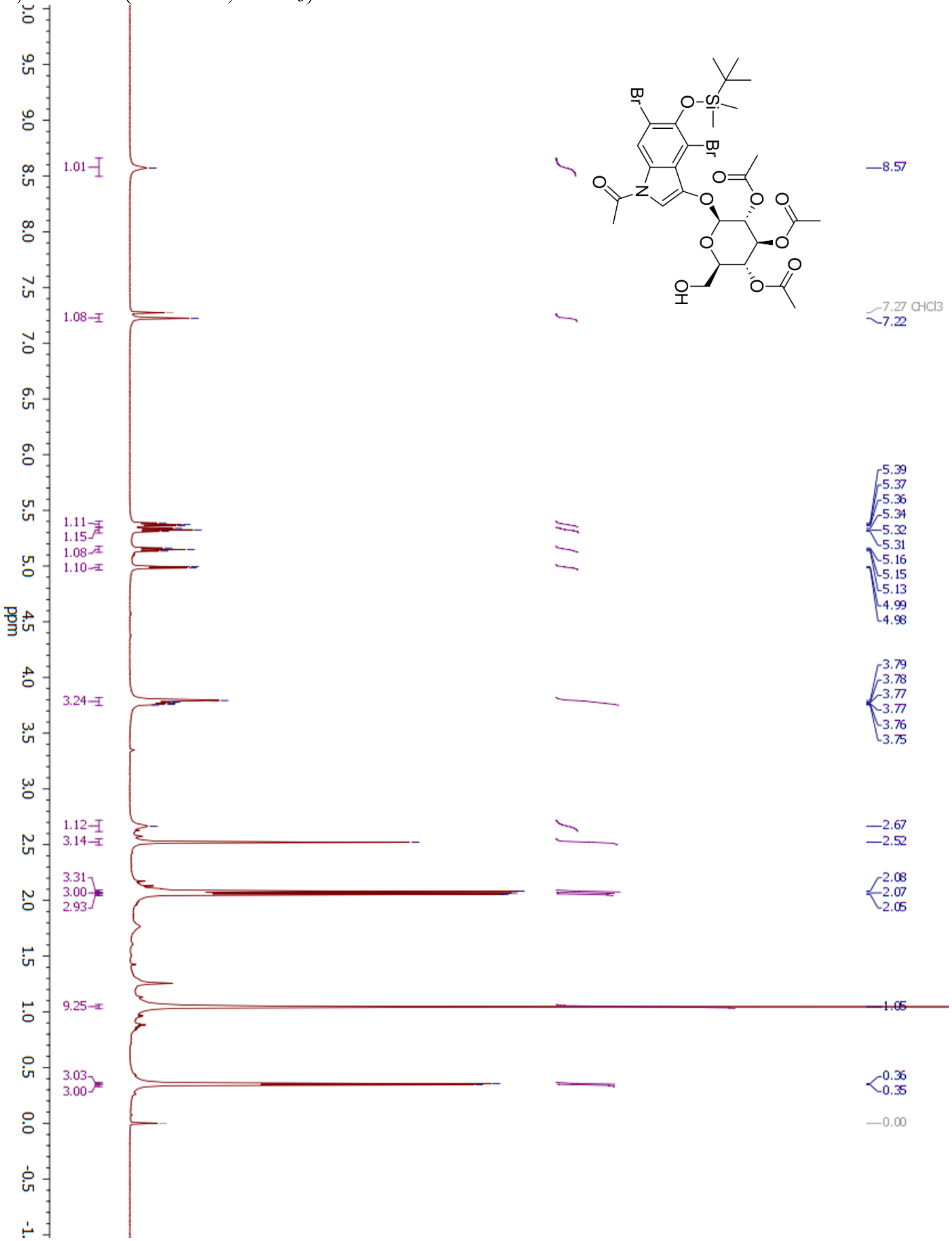

3, ^{13}C NMR (175 MHz, $CDCl_3$)

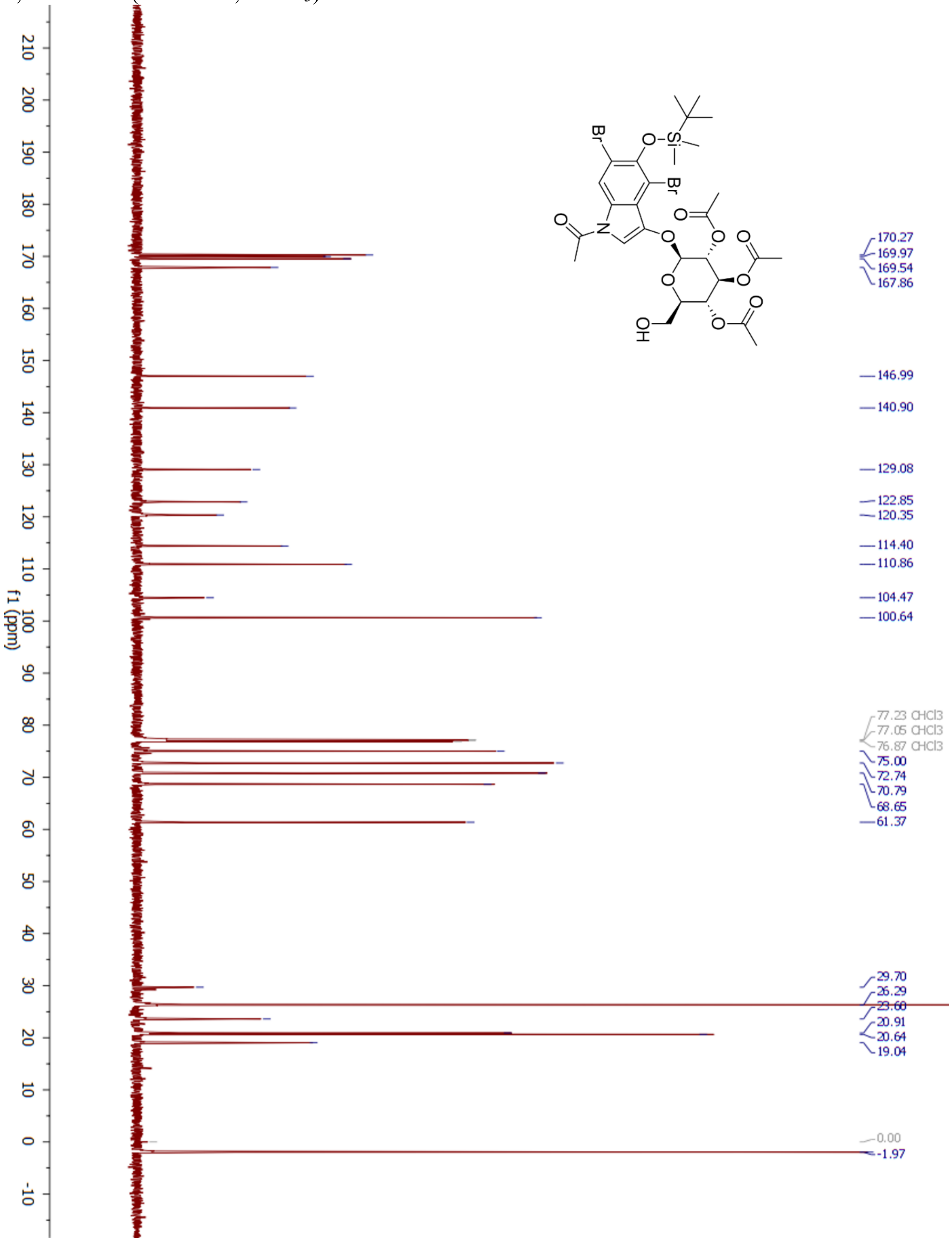

4, ^{1}H NMR (700 MHz, $CDCl_3$)

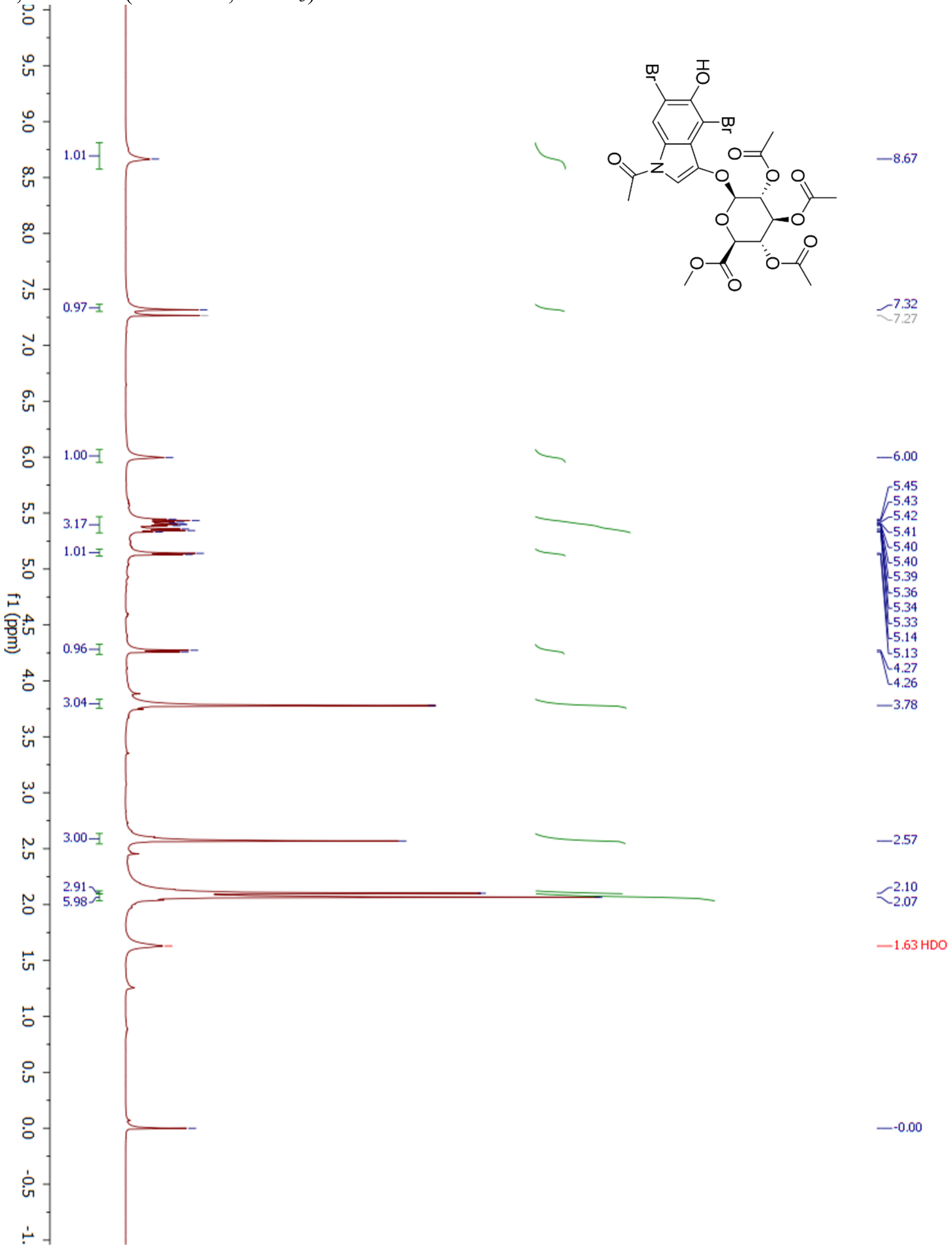

4, ^{13}C NMR (175 MHz, $CDCl_3$)

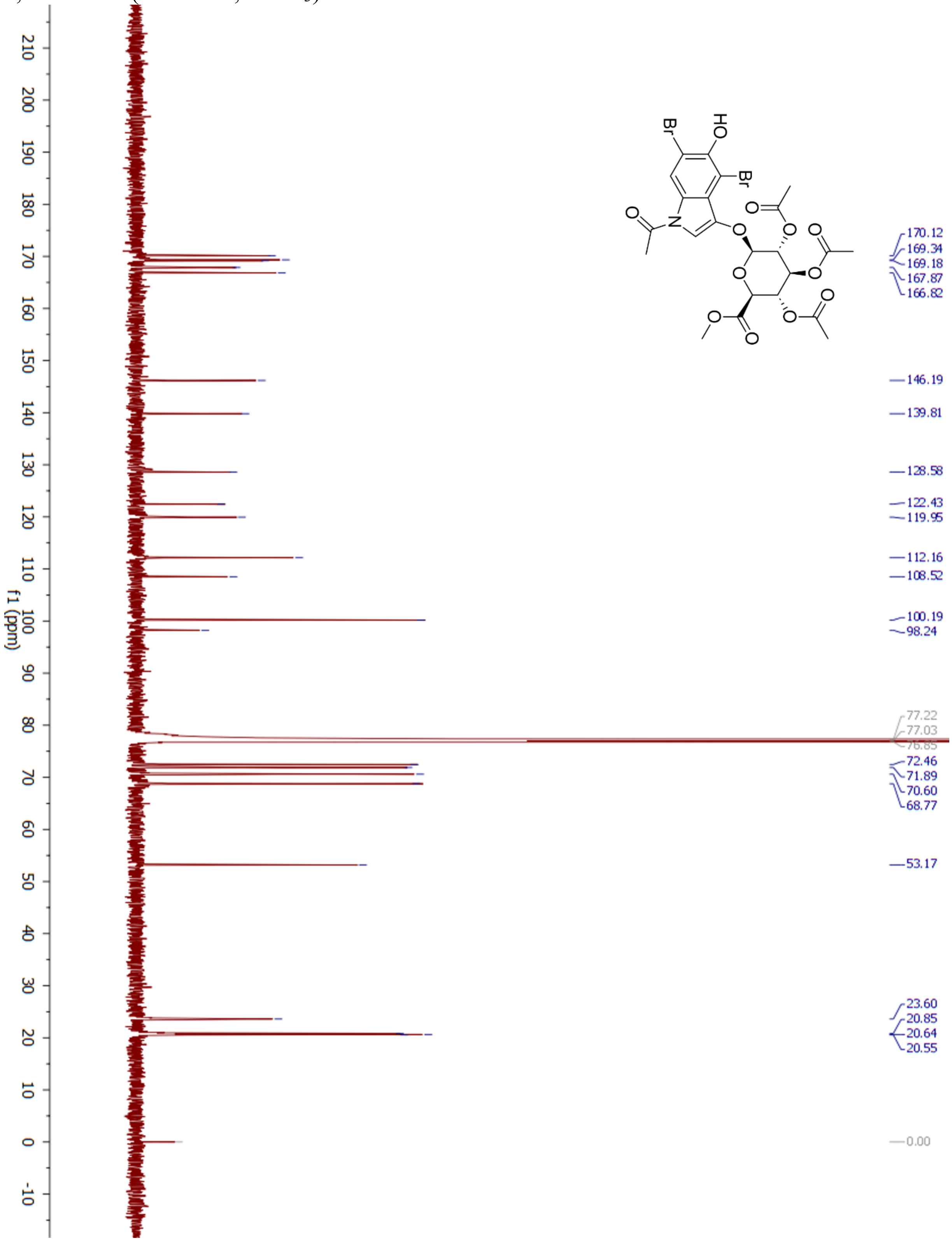

7, ^{1}H NMR (600 MHz, $CDCl_3$)

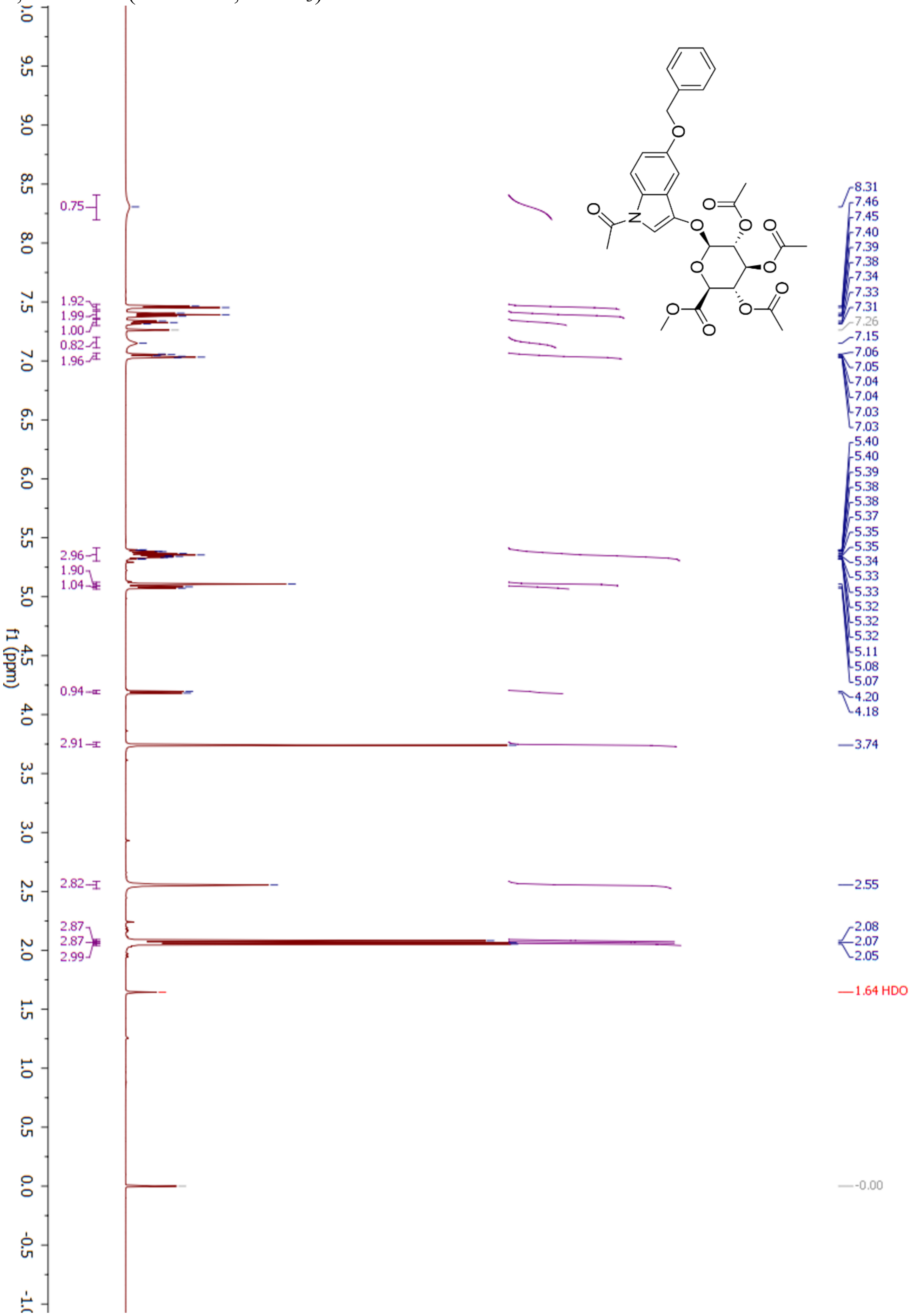

7, ^{13}C NMR (150 MHz, $CDCl_3$)

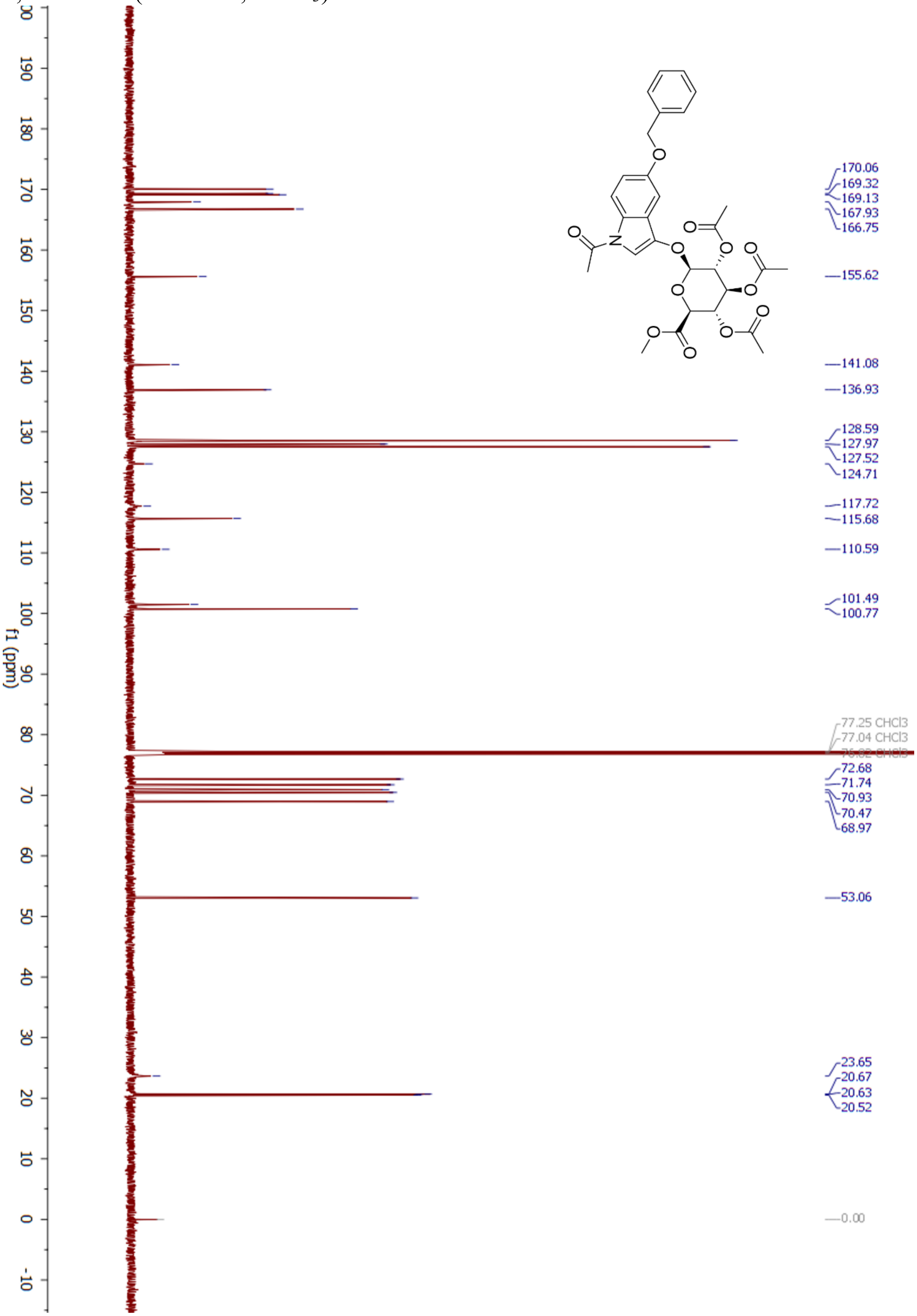

8, ^{1}H NMR (600 MHz, $CDCl_3$)

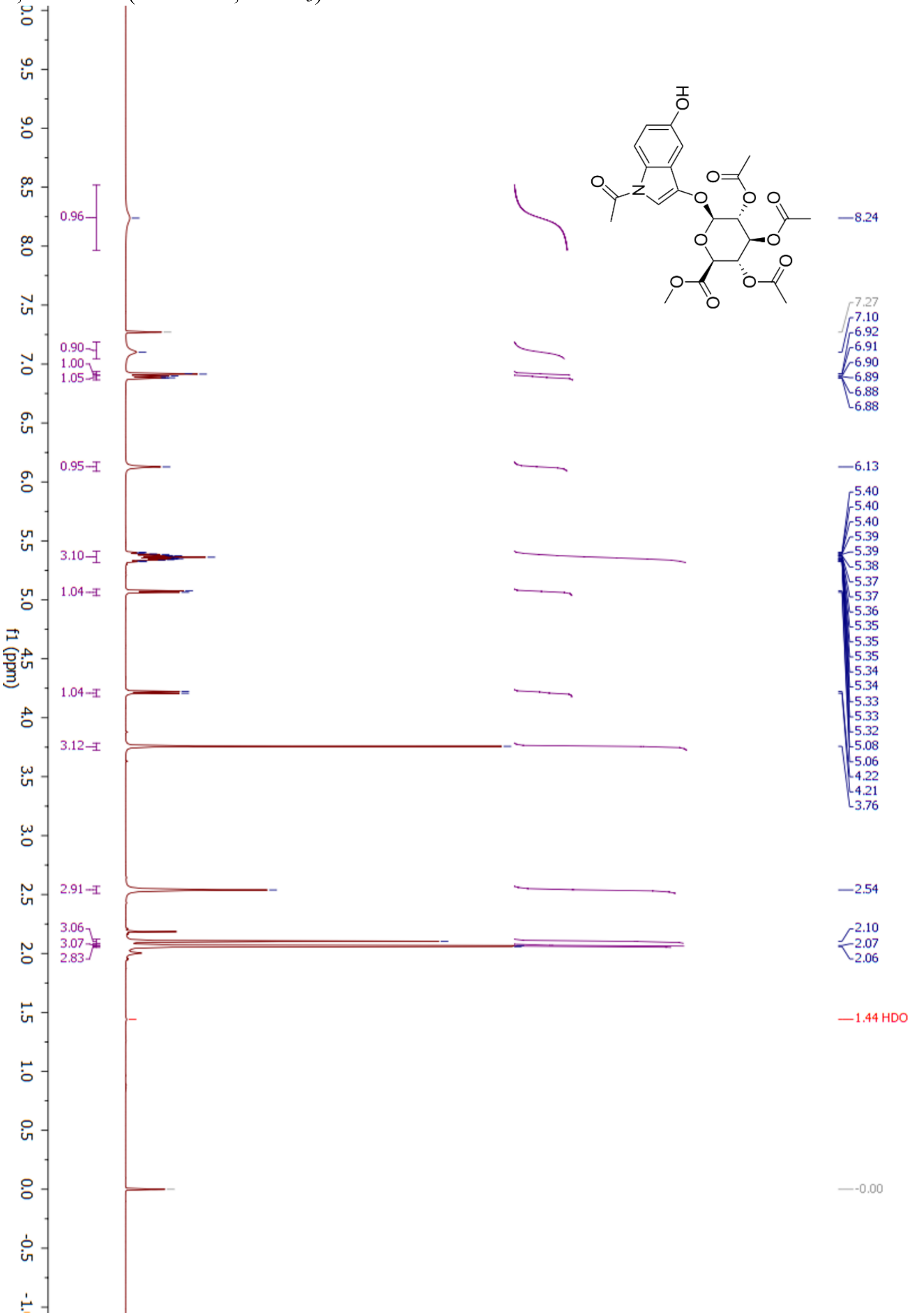

8, ^{13}C NMR (150 MHz, $CDCl_3$)

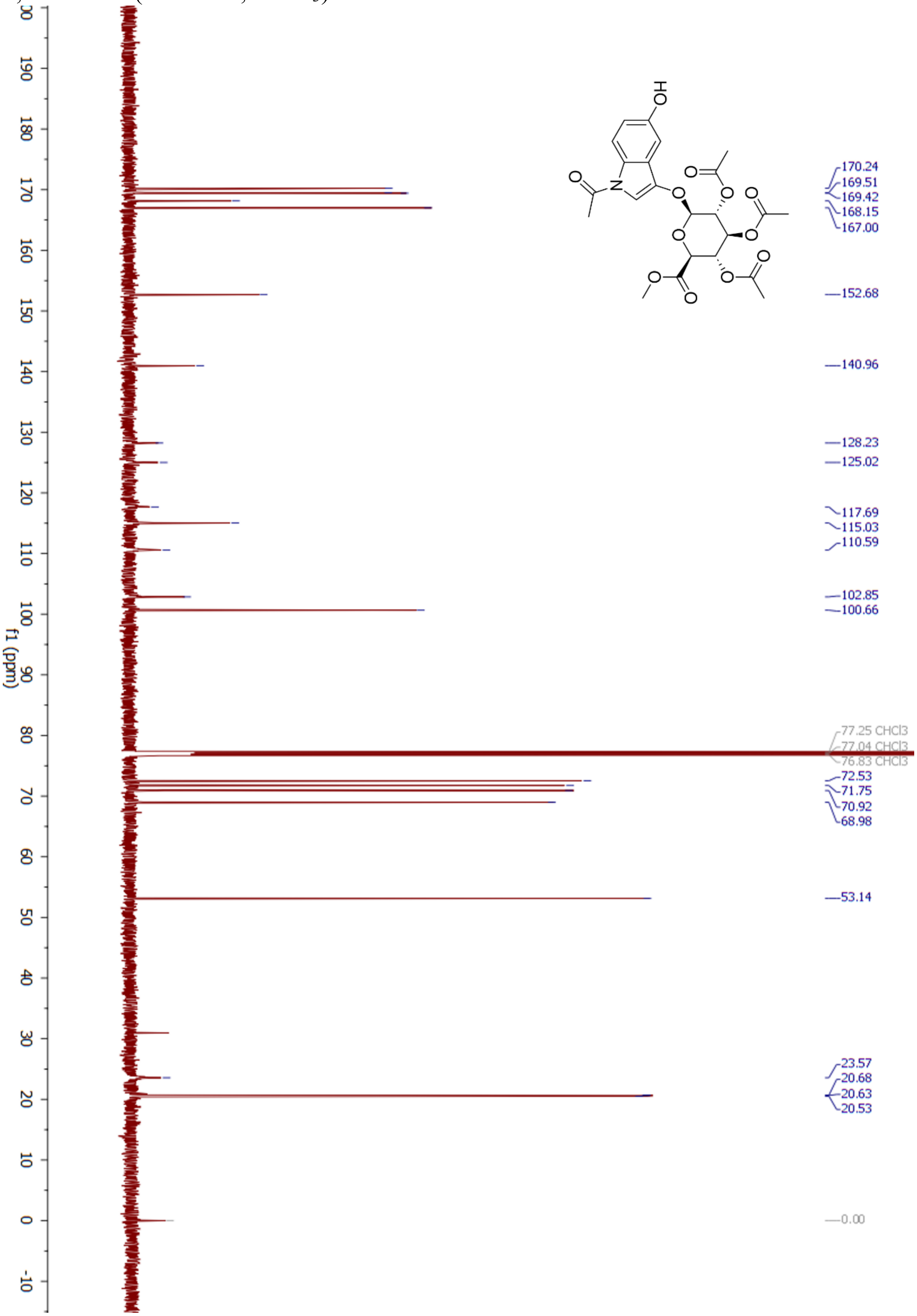

1-acetyl-5-{[(1*R*,8*S*,9*s*)-bicyclo[6.1.0]non-4-yn-9-yl]methoxy}-4,6-dibromo-5-hydroxy-1*H*-indol-3-yl 2,3,4-tri-*O*-acetyl-β-D-glucopyranosiduronic acid methyl ester, ^{1}H NMR (500 MHz, $CDCl_3$)

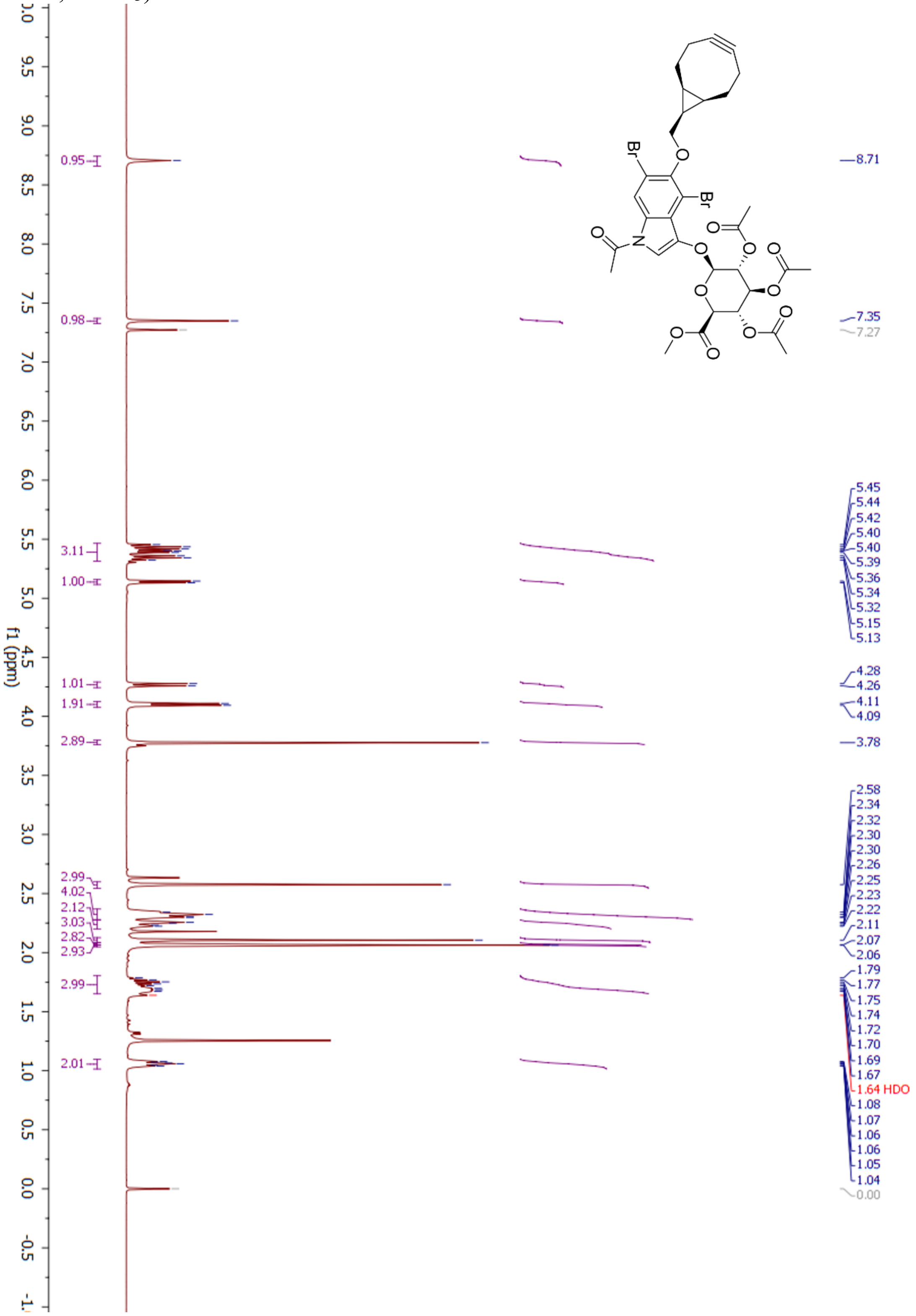

9, ^{1}H NMR (700 MHz, $CDCl_3$)

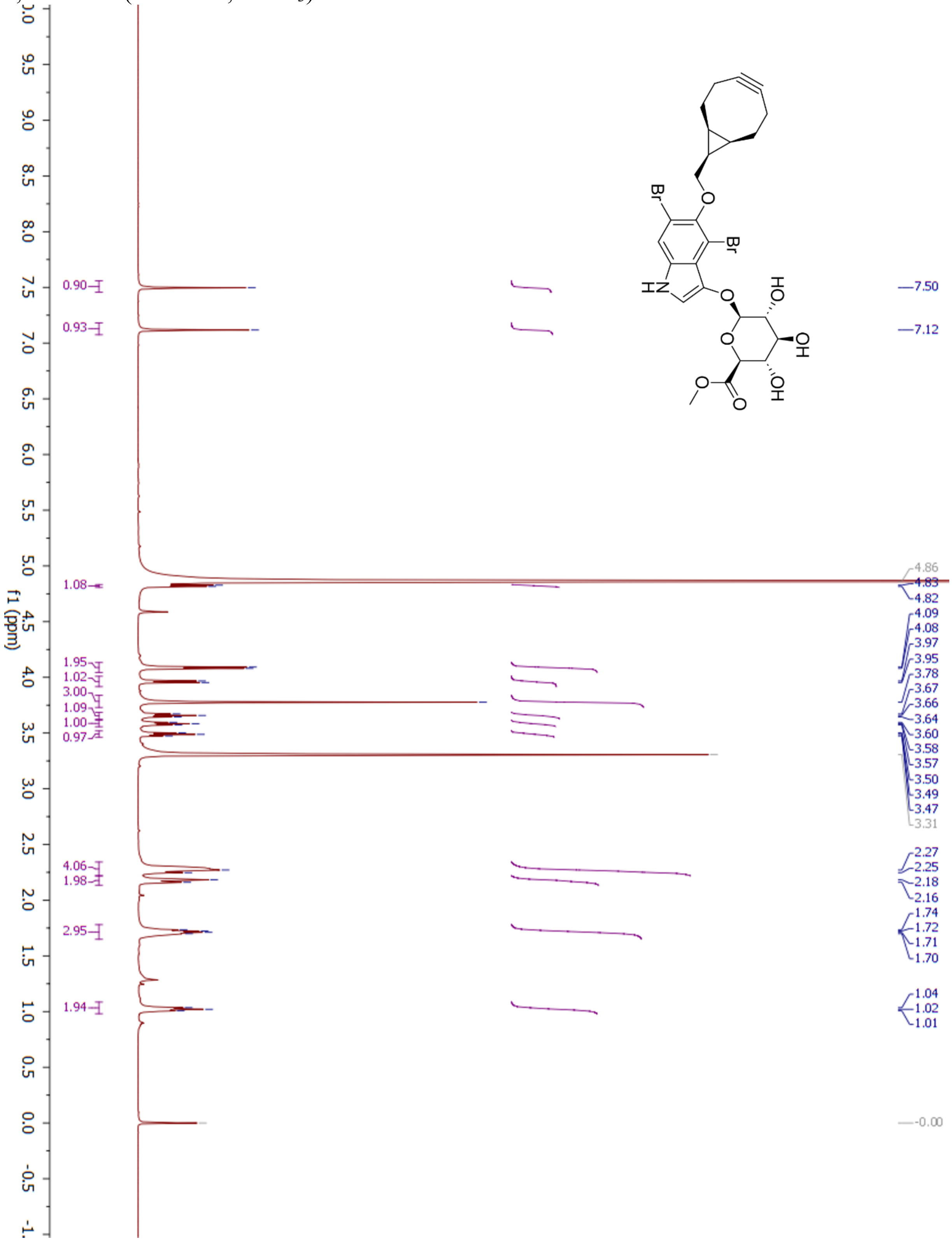

9, ^{13}C NMR (175 MHz, $CDCl_3$)

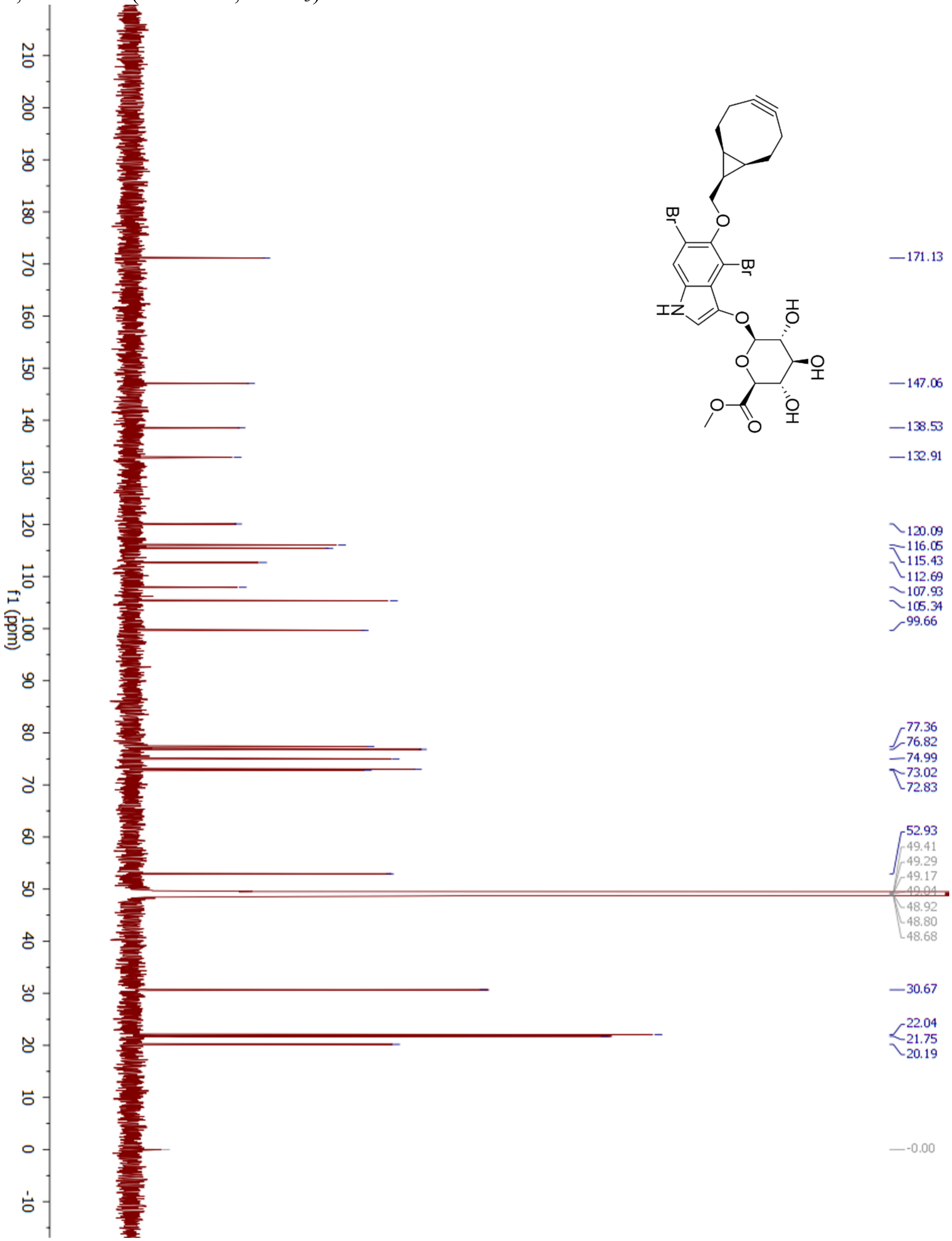

10, ^{1}H NMR (700 MHz, CD_3OD)

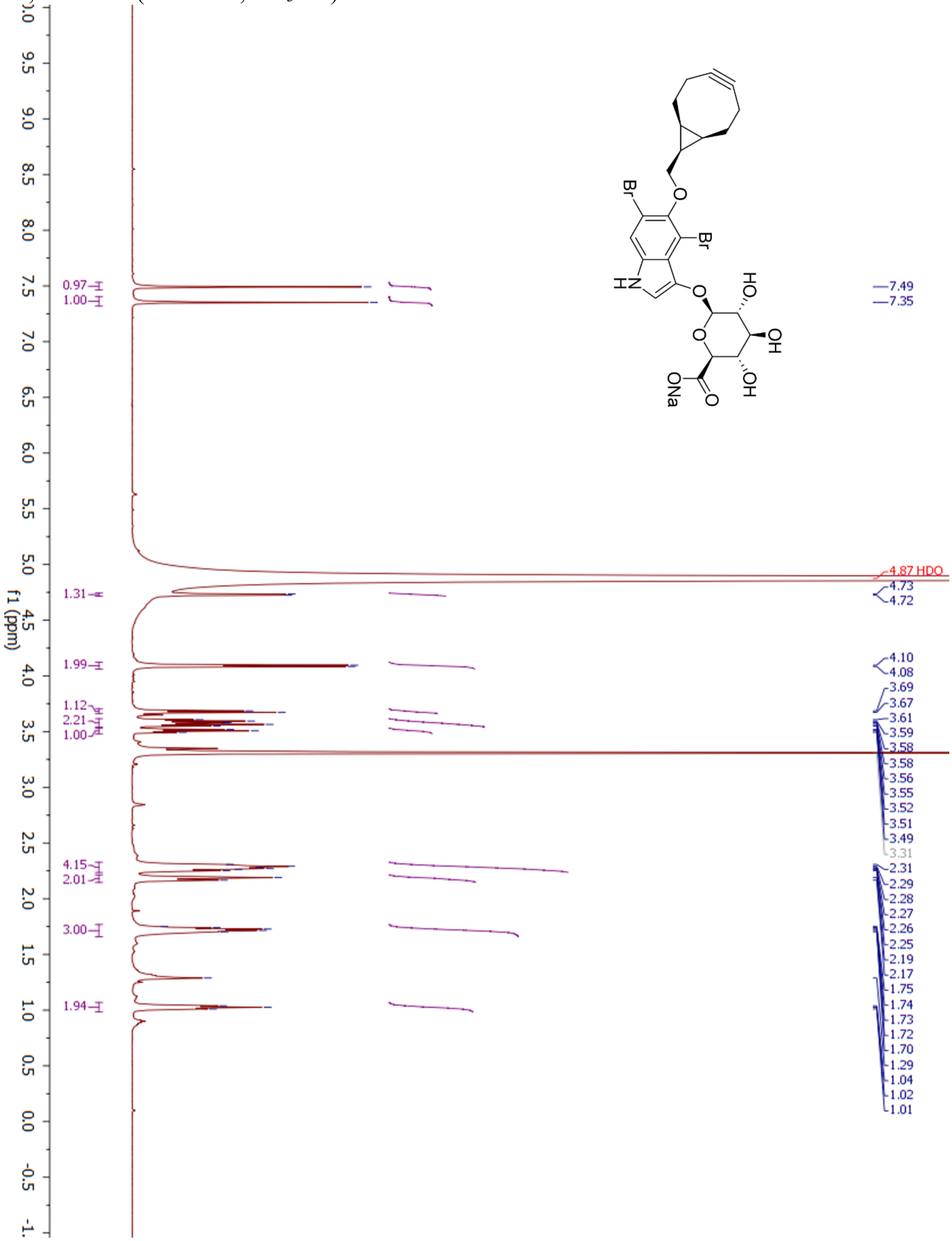

10, ^{13}C NMR (175 MHz, CD_3OD)

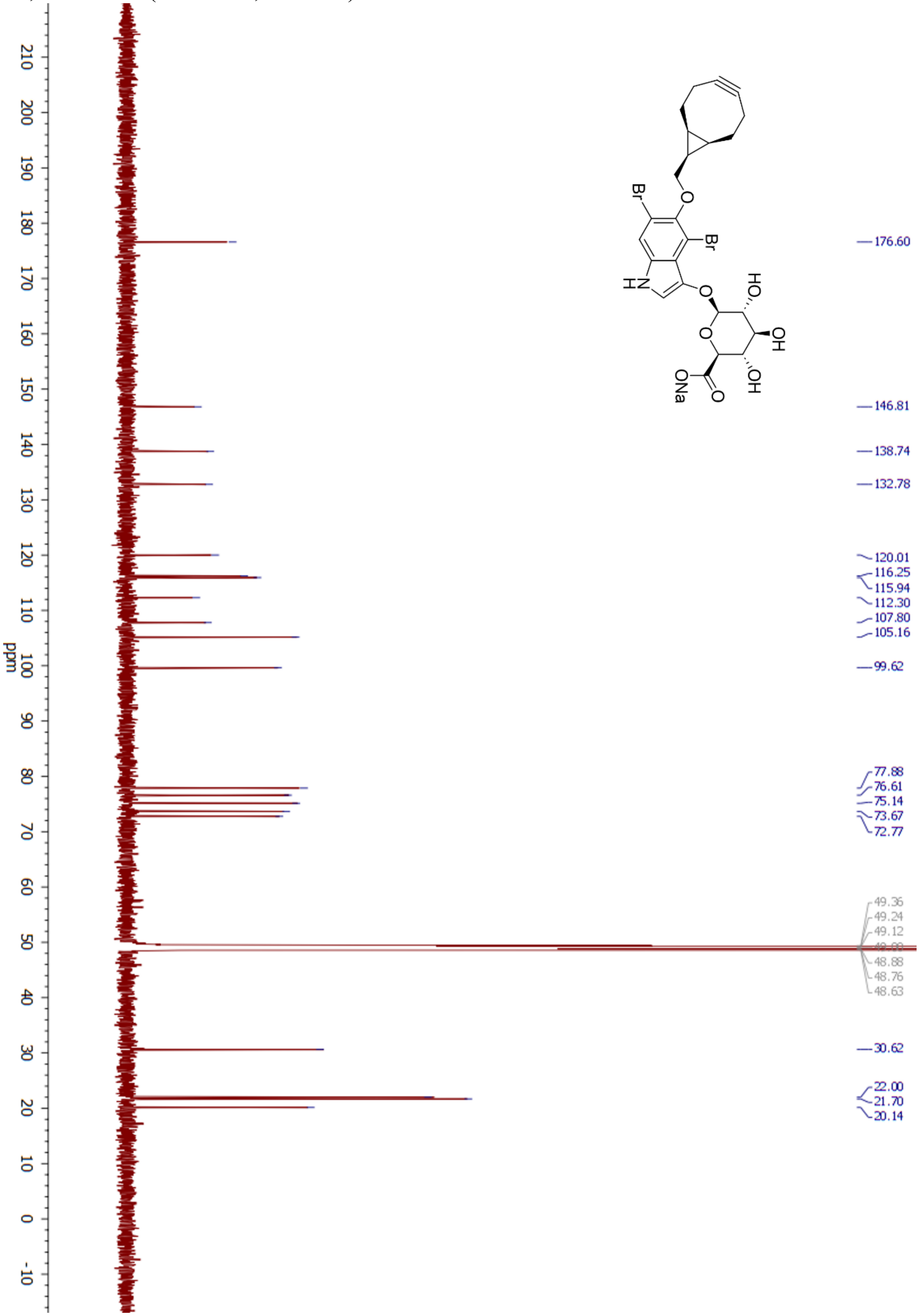

11, ^{1}H NMR (500 MHz, $CDCl_3$)

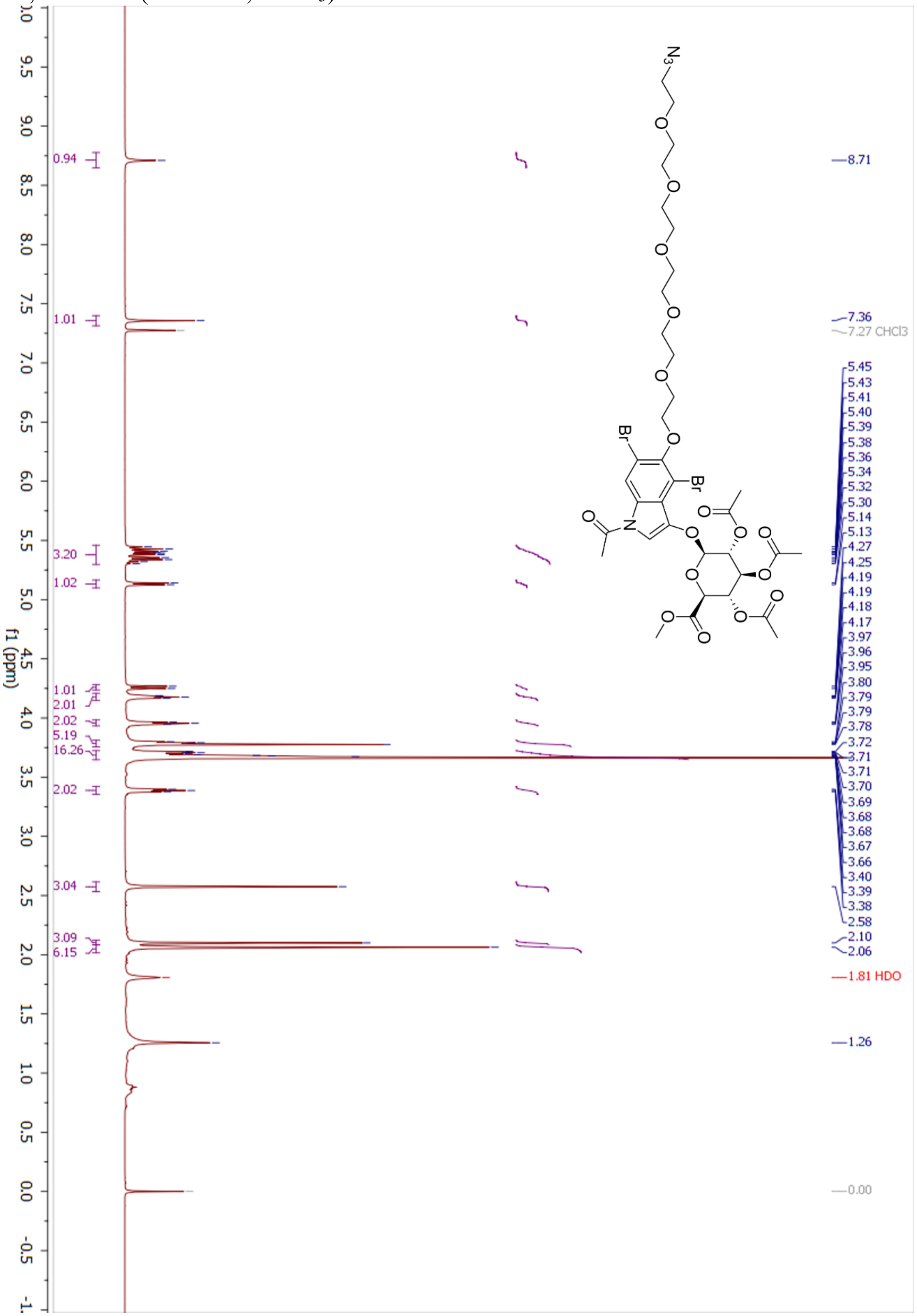

11, ^{13}C NMR (125 MHz, $CDCl_3$)

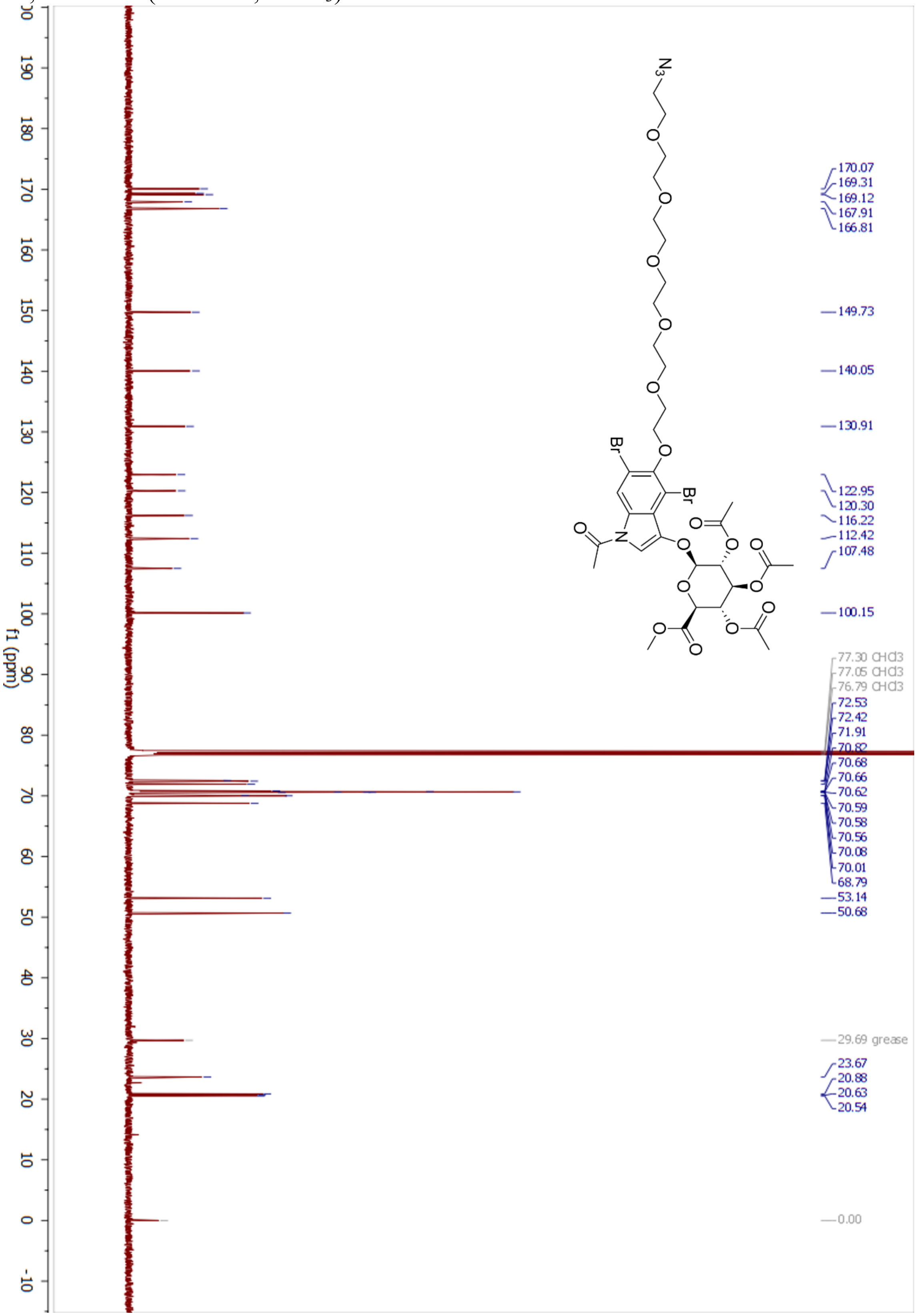

12, ^{1}H NMR (500 MHz, CD_3OD)

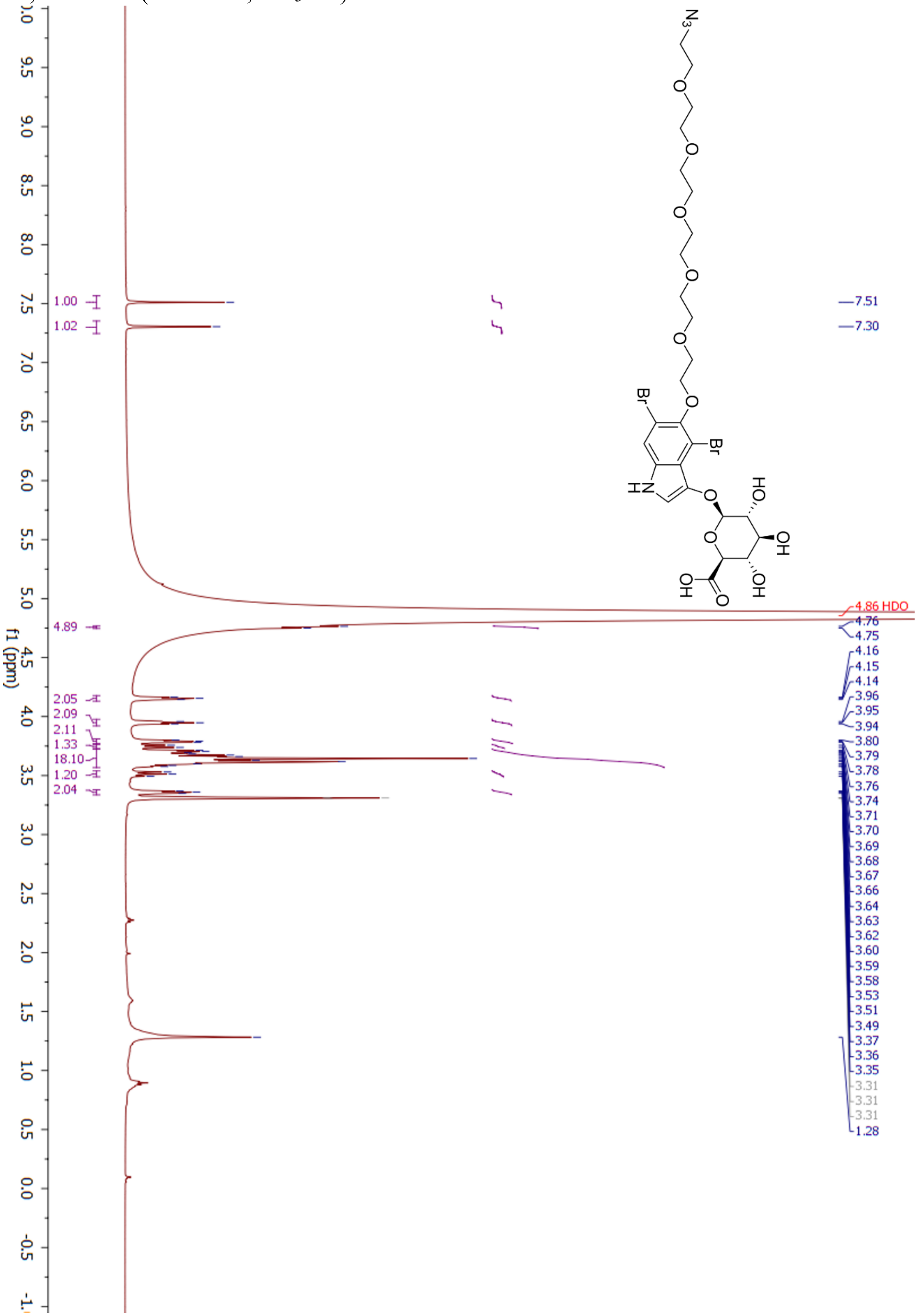

12, ^{13}C NMR (125 MHz, CD_3OD)

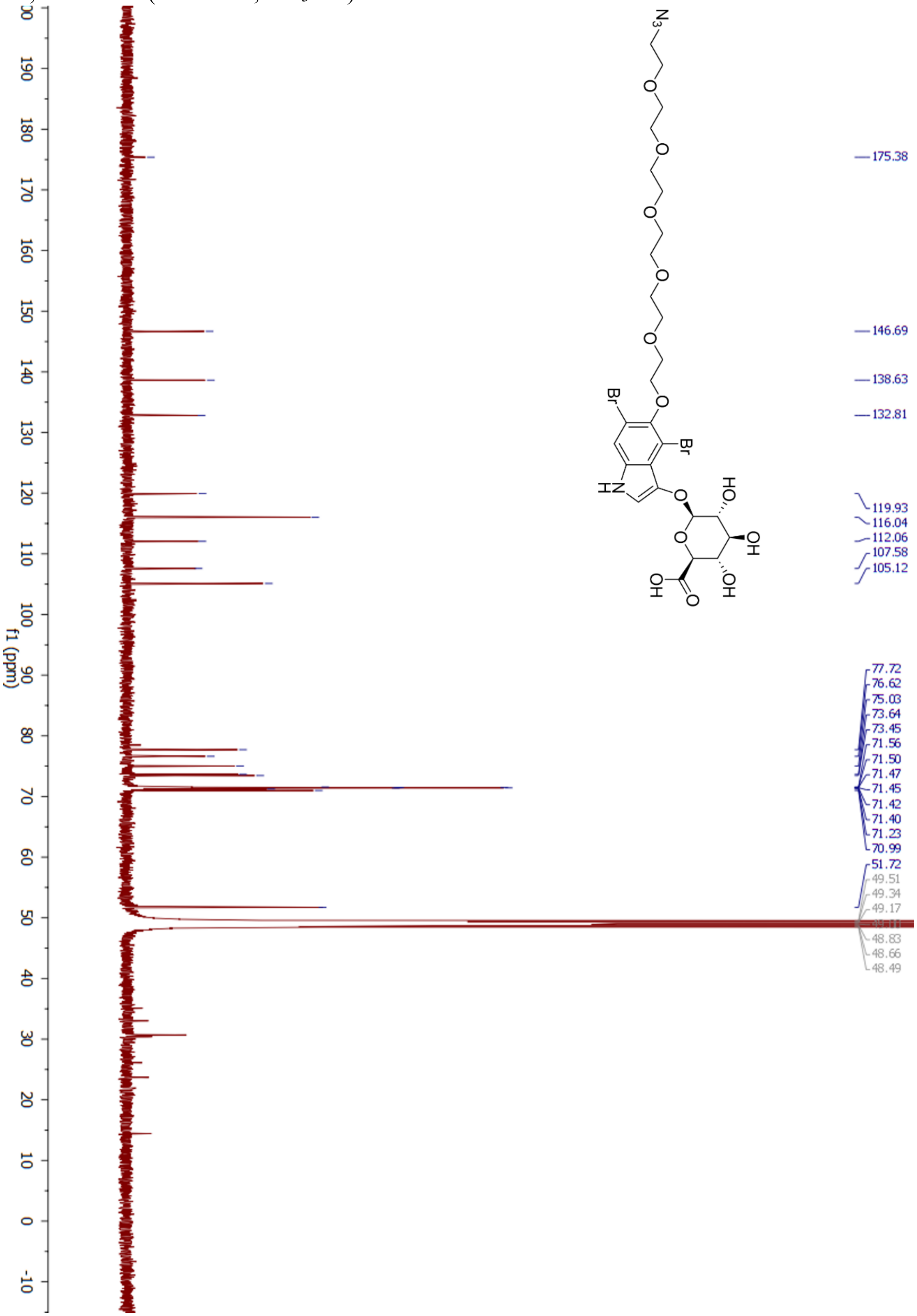

13, ^{1}H NMR (600 MHz, $CDCl_3$)

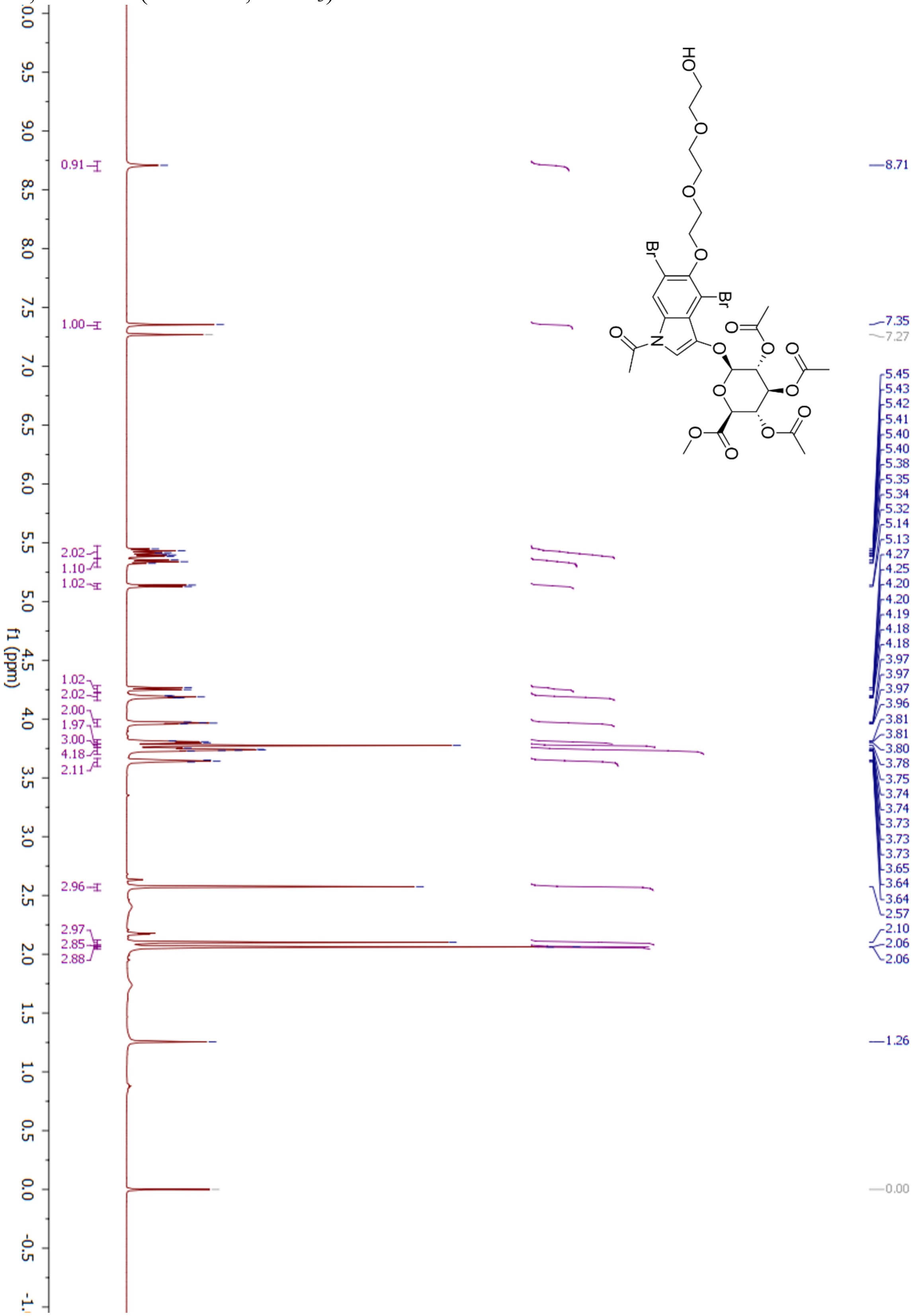

13, ^{13}C NMR (150 MHz, $CDCl_3$)

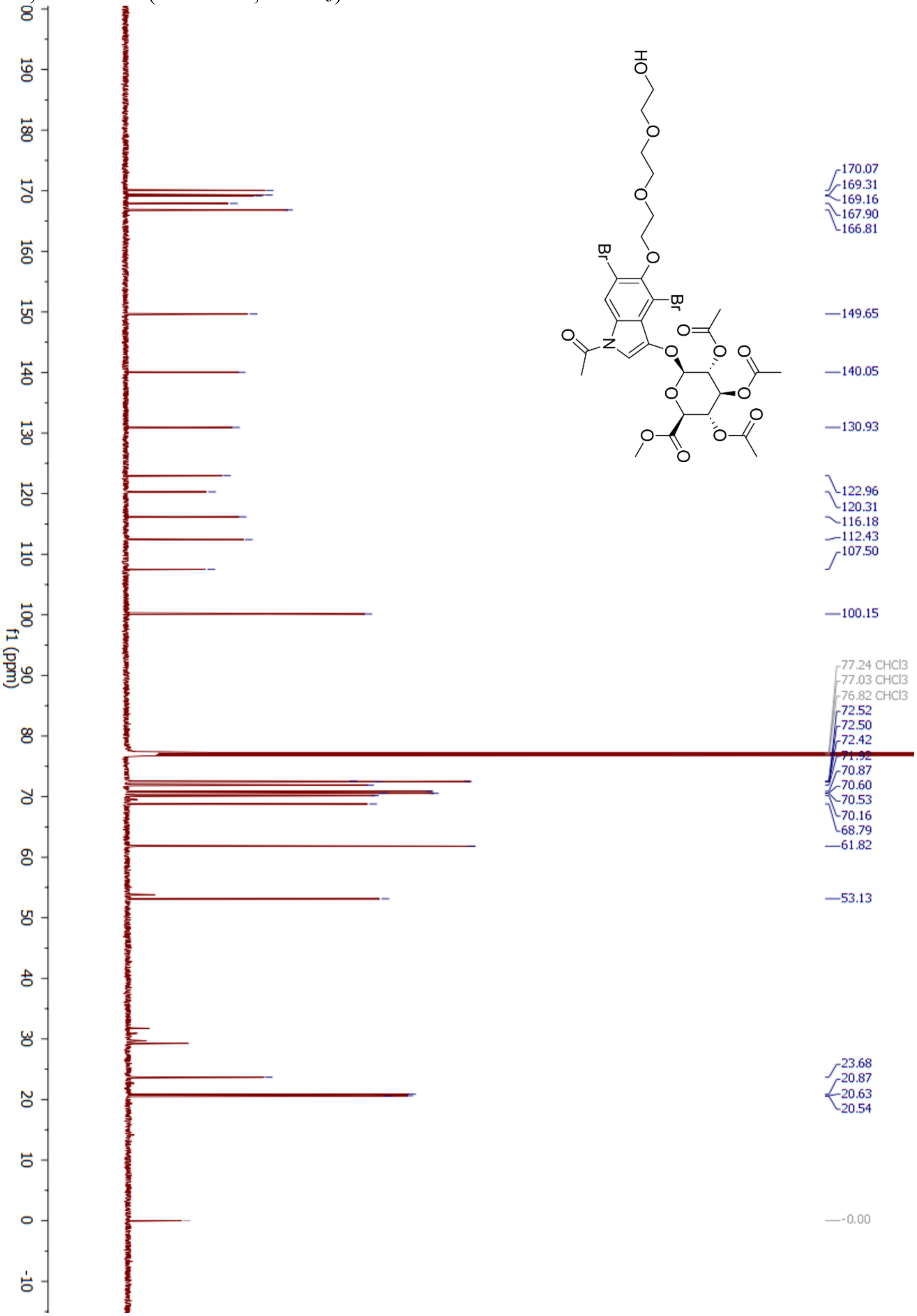

14, ^{1}H NMR (500 MHz, $CDCl_3$)

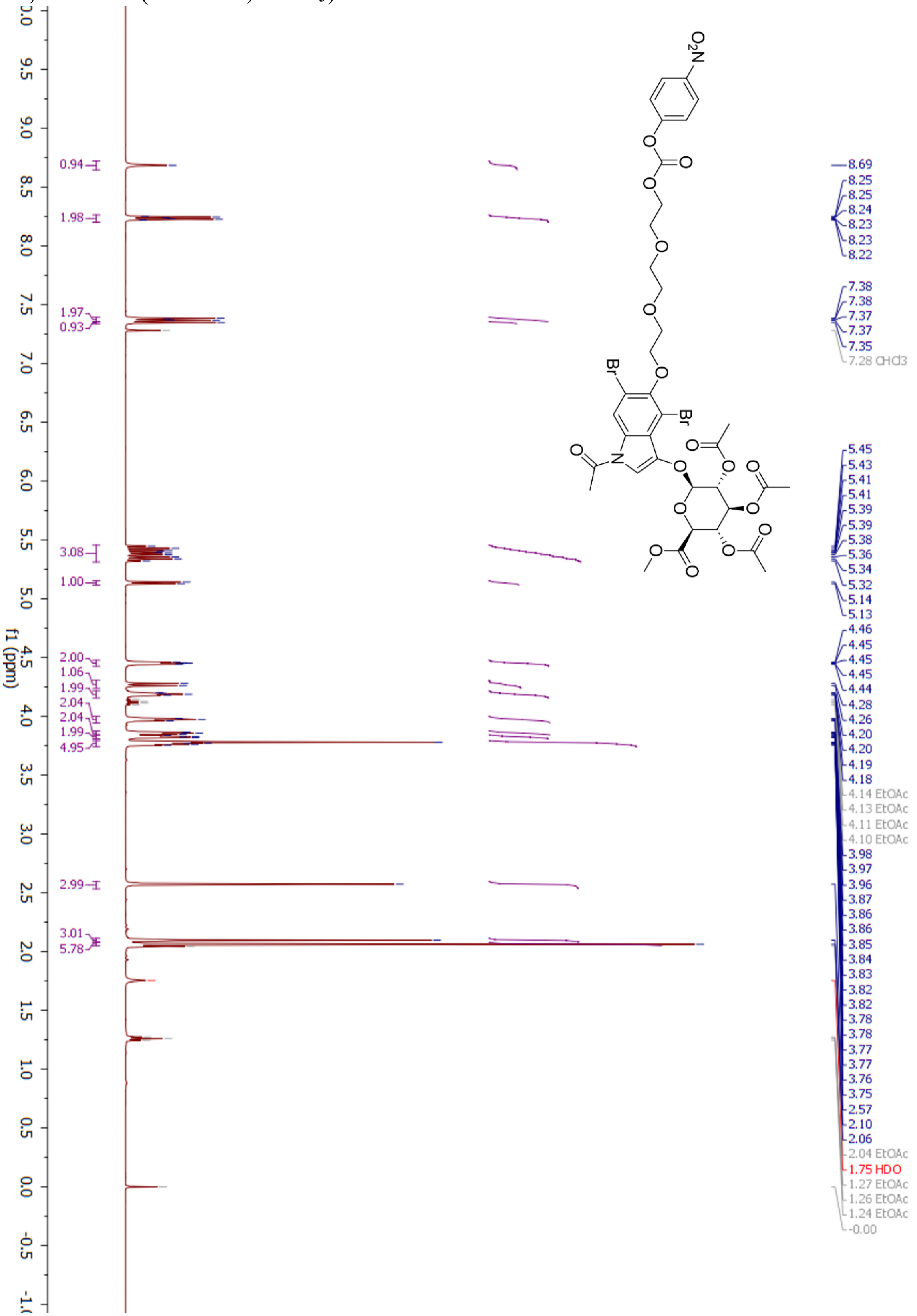

14, ^{13}C NMR (125 MHz, $CDCl_3$)

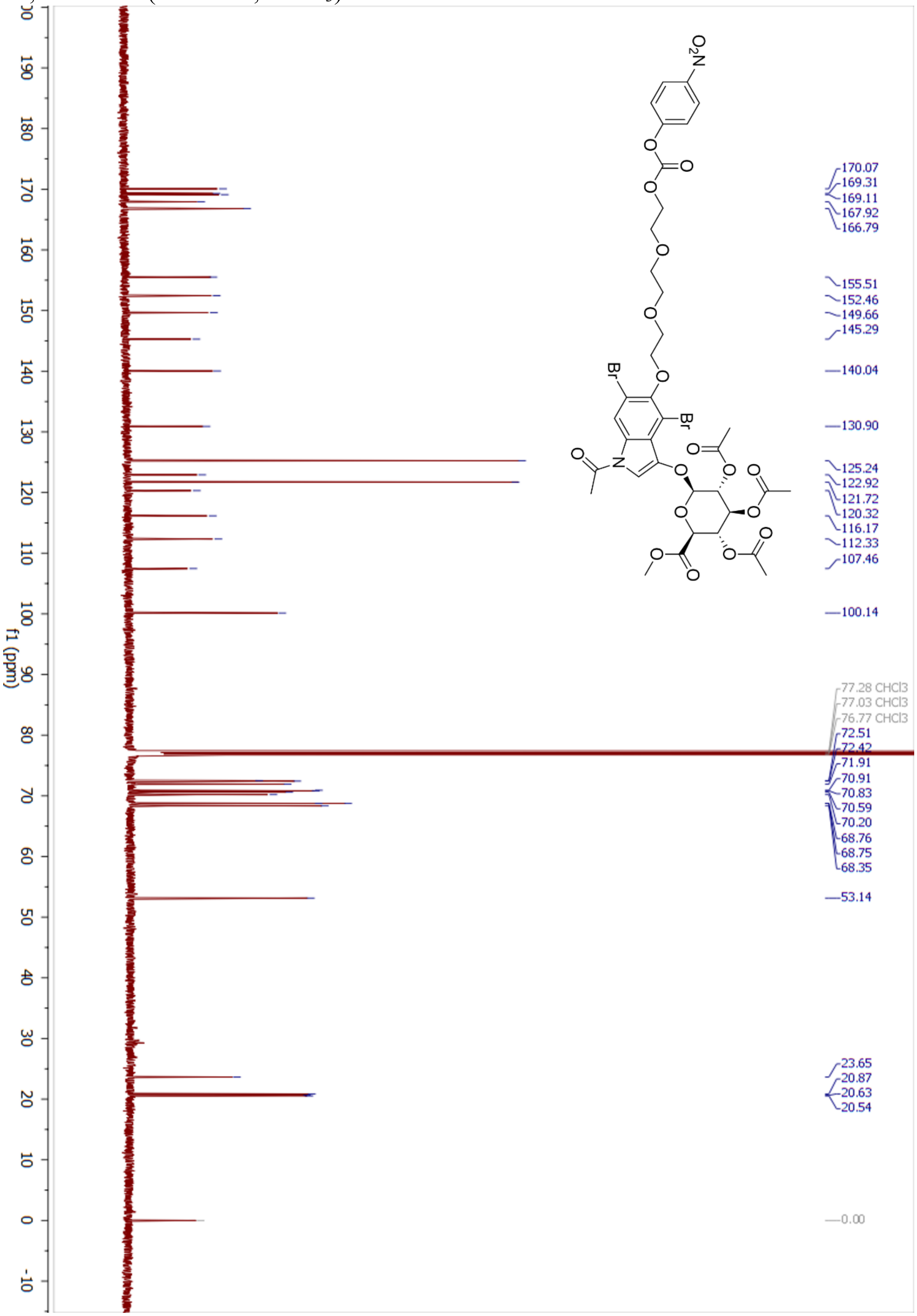

15, ^1H NMR (500 MHz, $CDCl_3$)

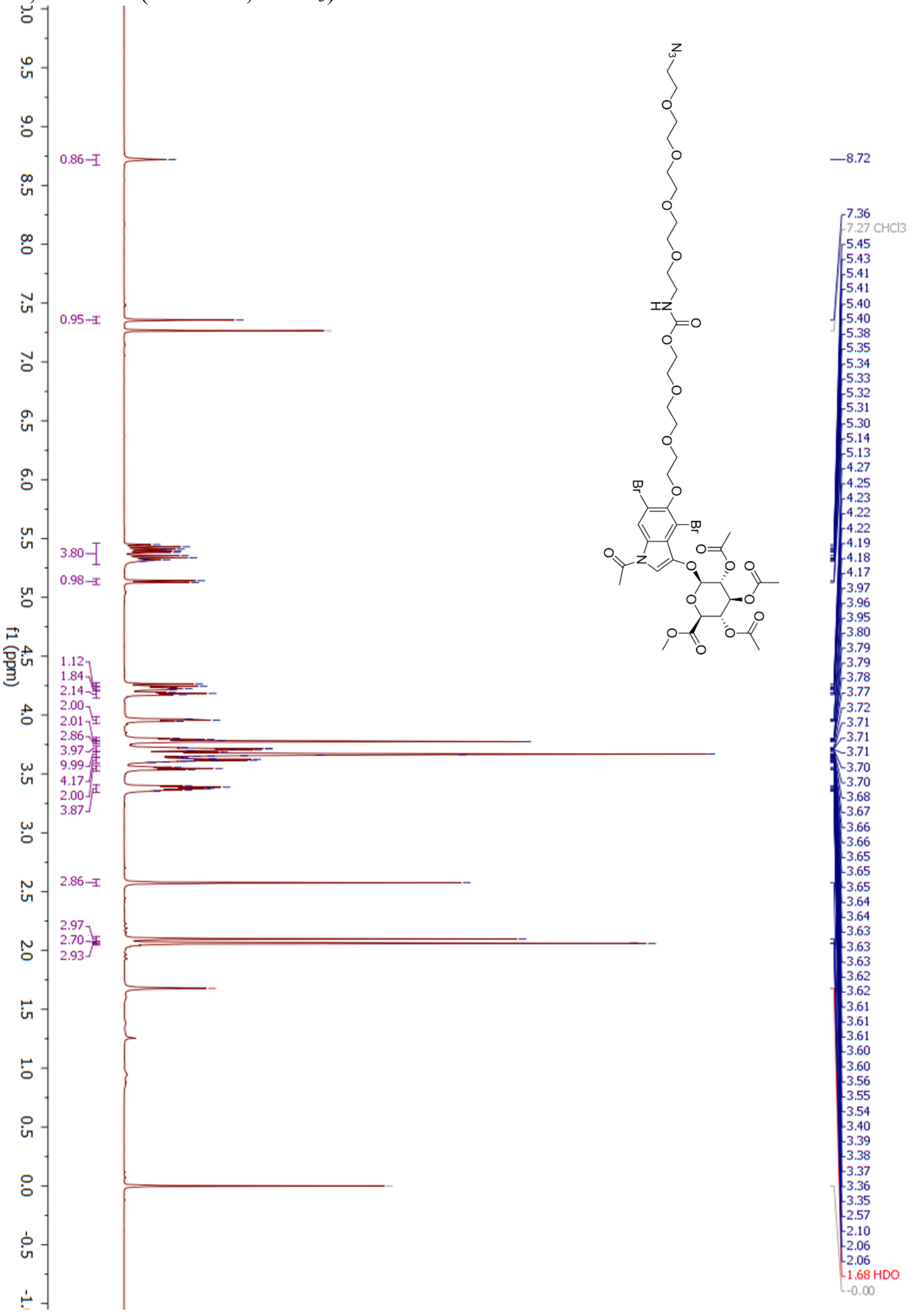

15, ^{13}C NMR (125 MHz, $CDCl_3$)

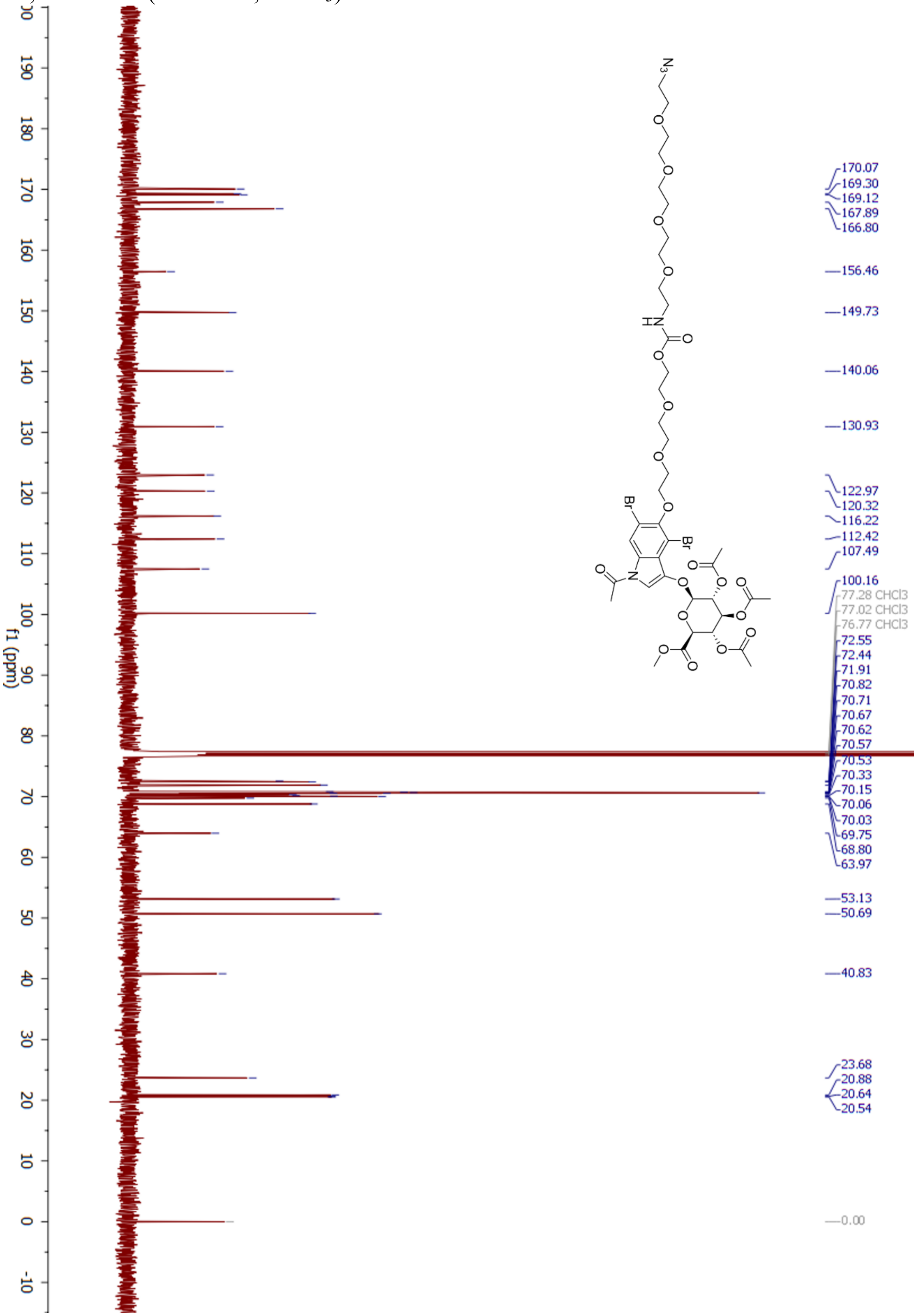

16, ^{1}H NMR (500 MHz, $CDCl_3$)

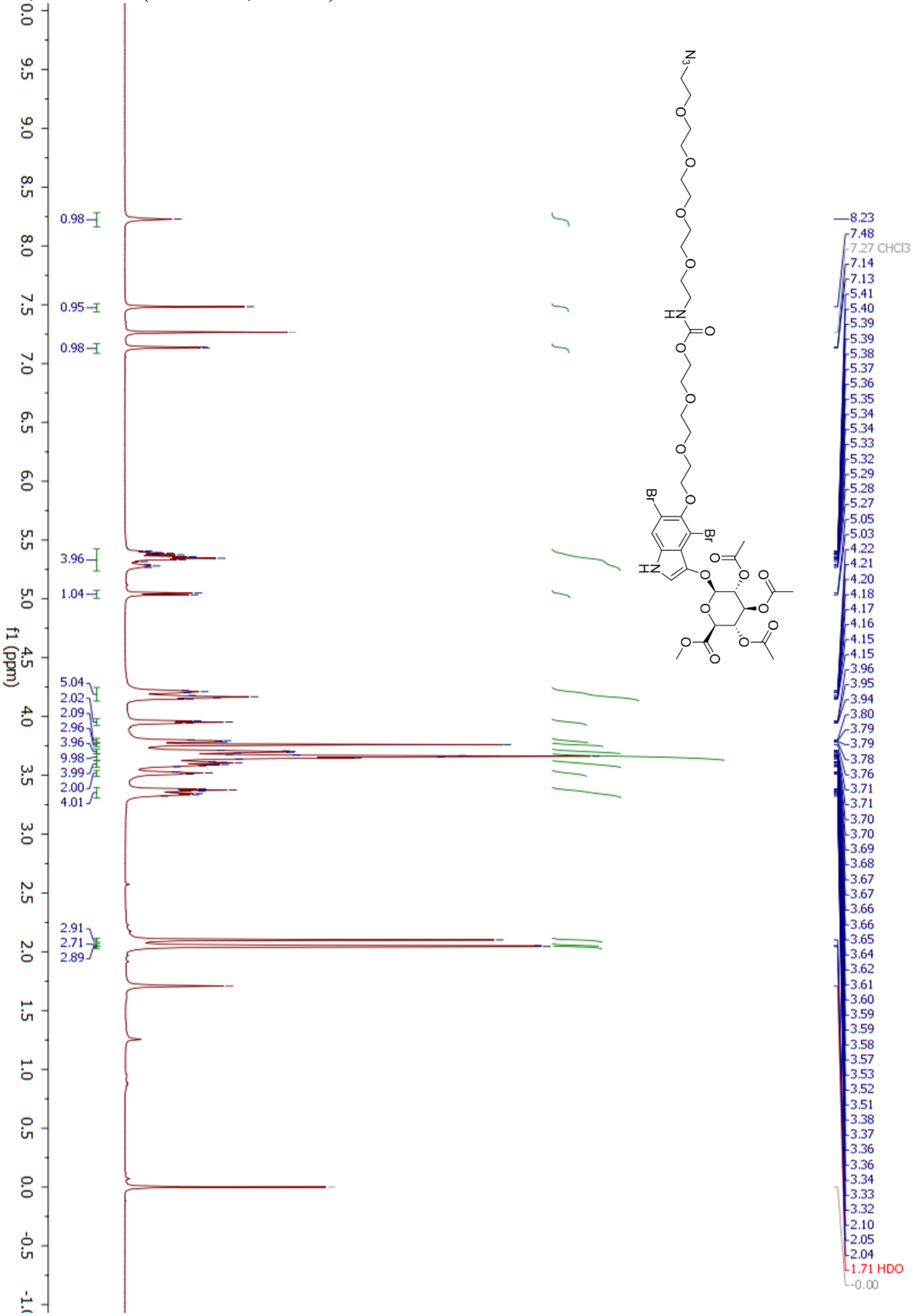

16, ^{13}C NMR (125 MHz, $CDCl_3$)

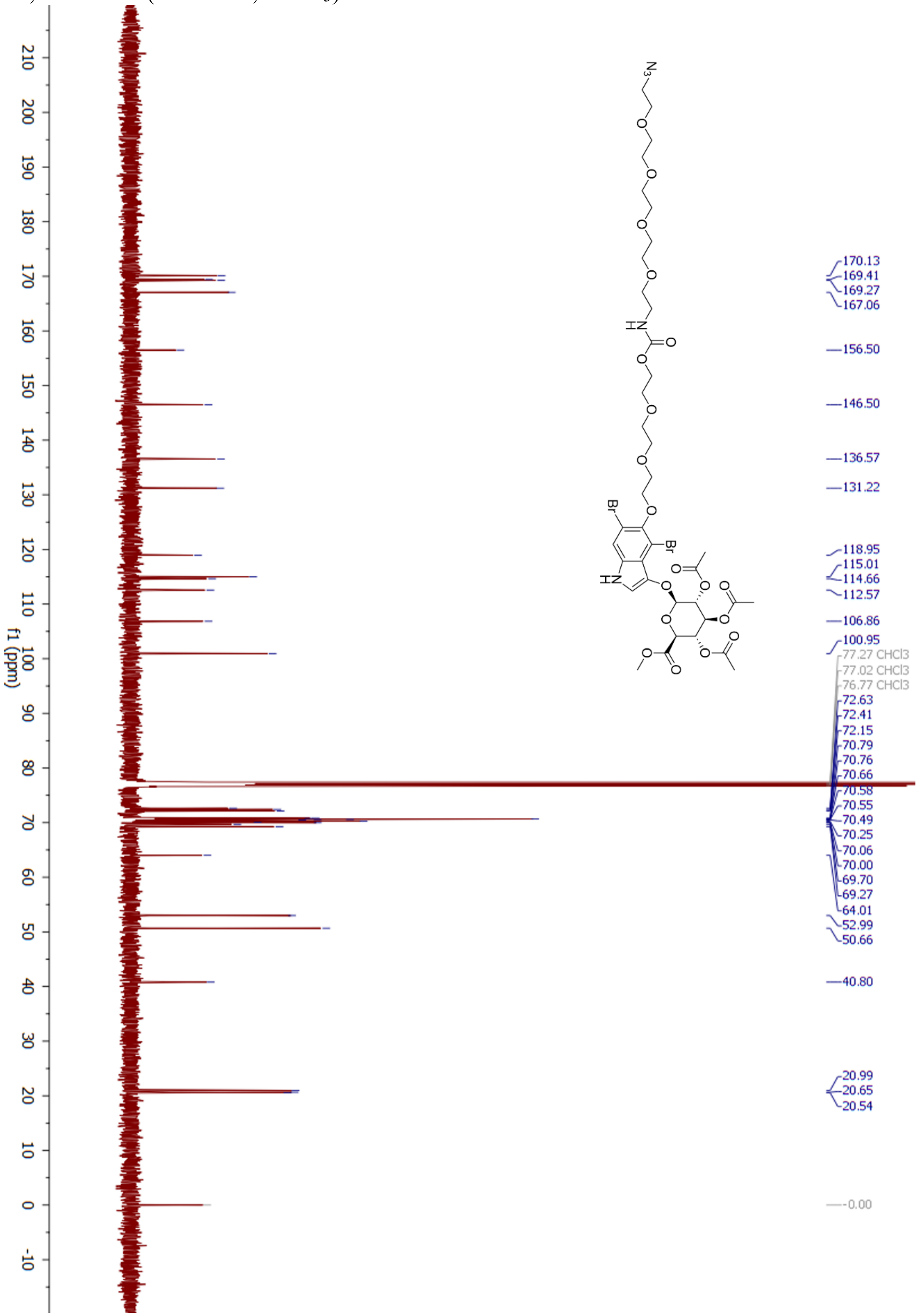

17, ^{1}H NMR (500 MHz, CD_3OD)

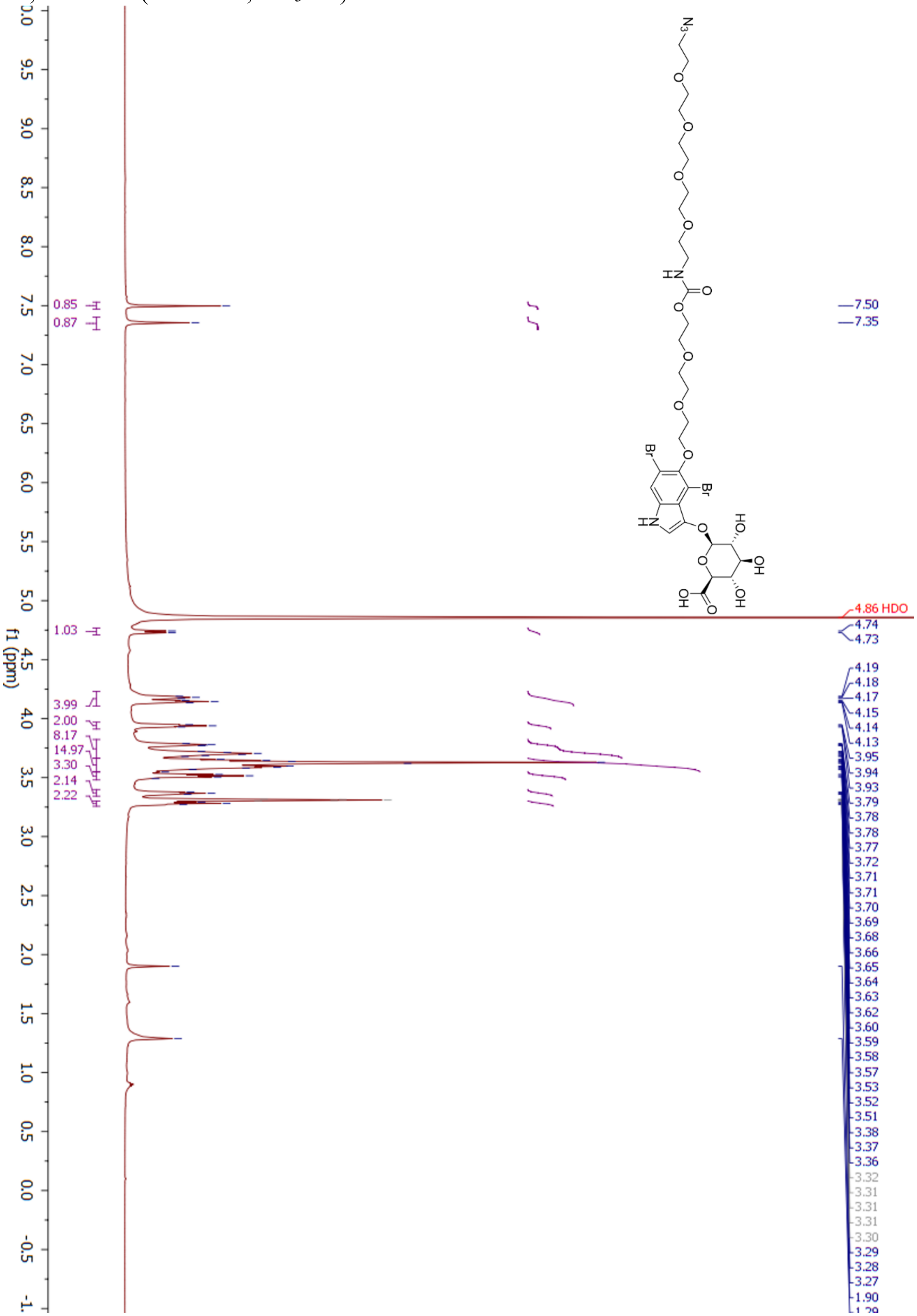

17, ^{13}C NMR (125 MHz, CD_3OD)

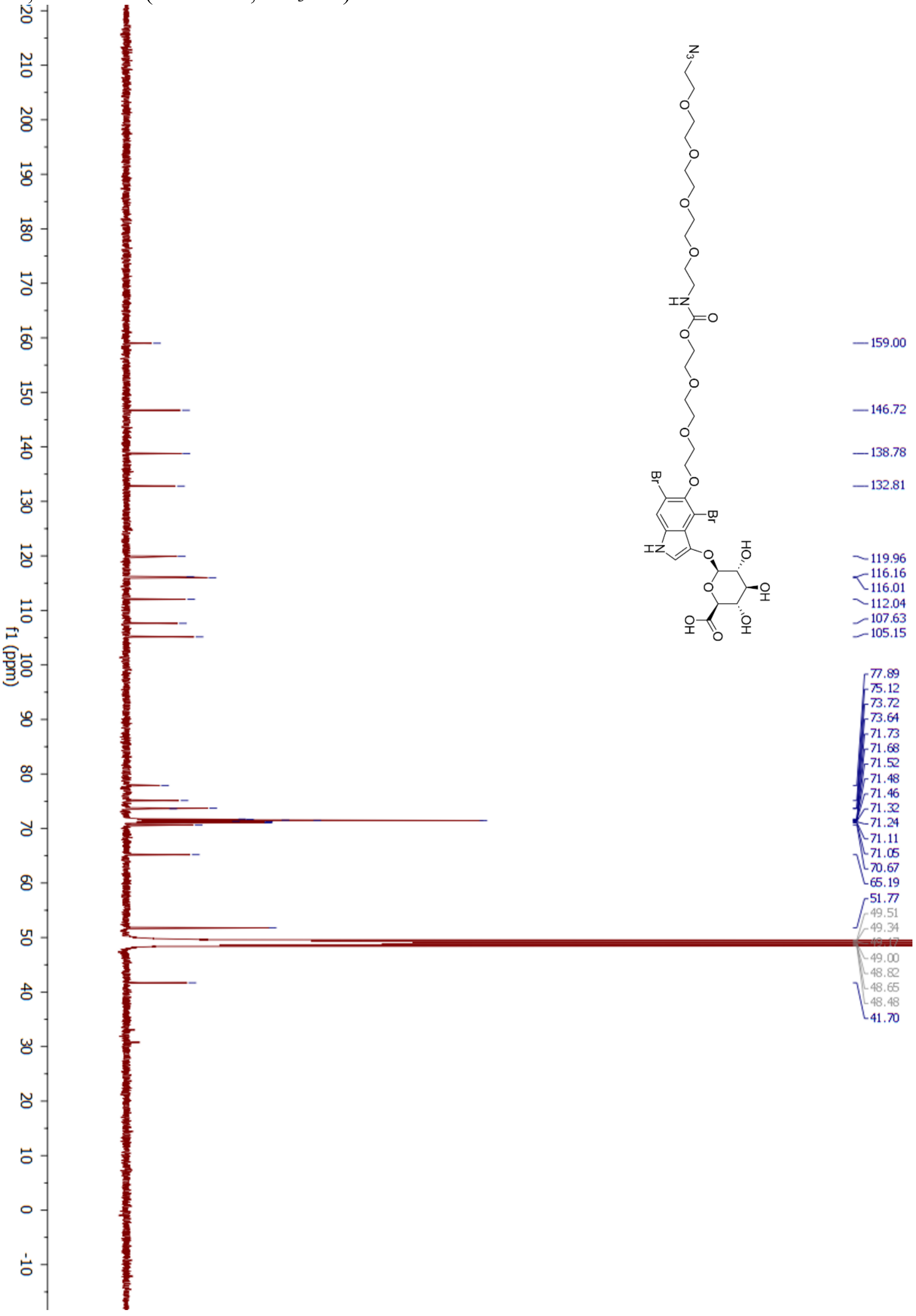

18, ^{1}H NMR (600 MHz, $CDCl_3$)

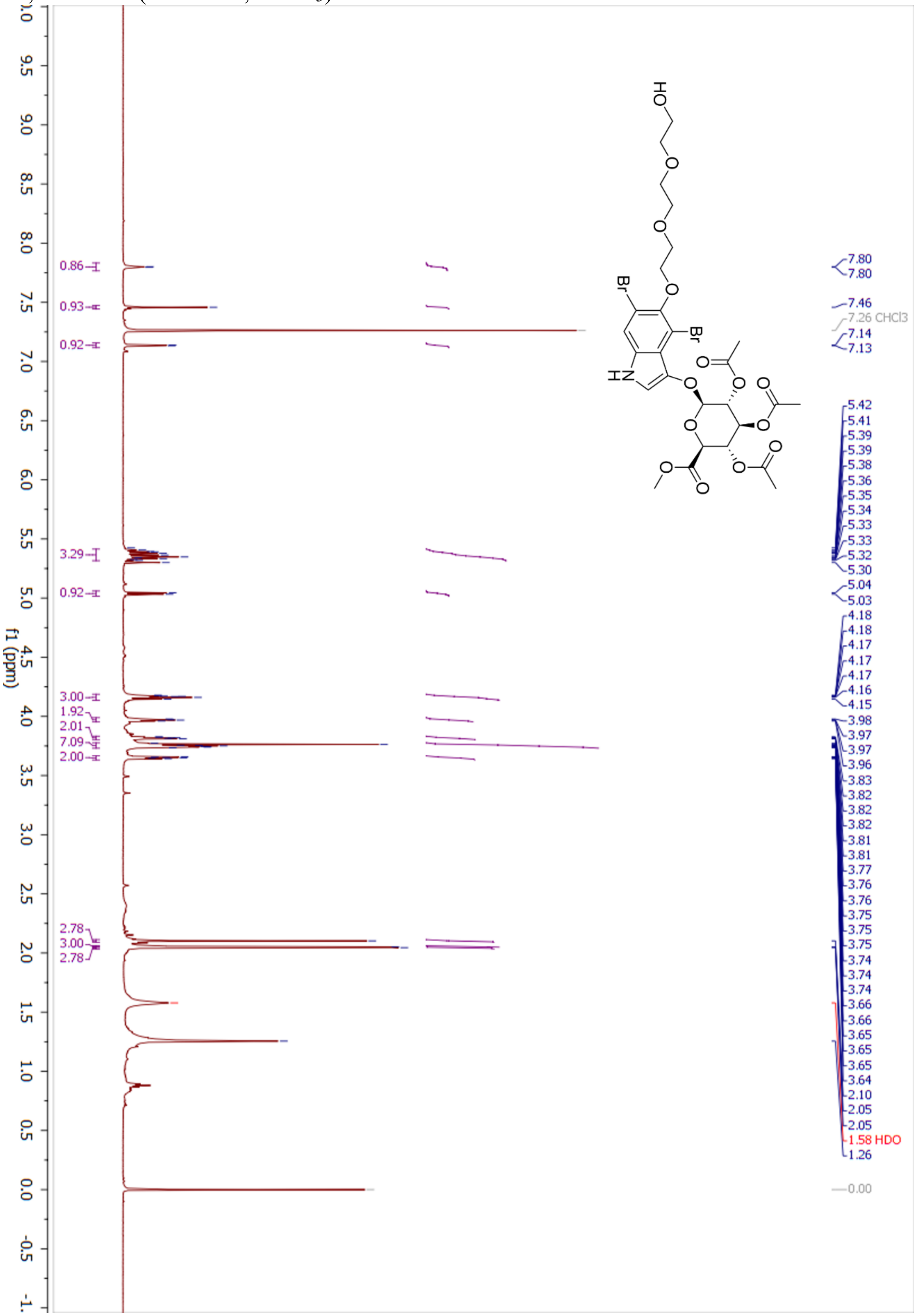

19, ^{1}H NMR (500 MHz, CD_3OD)

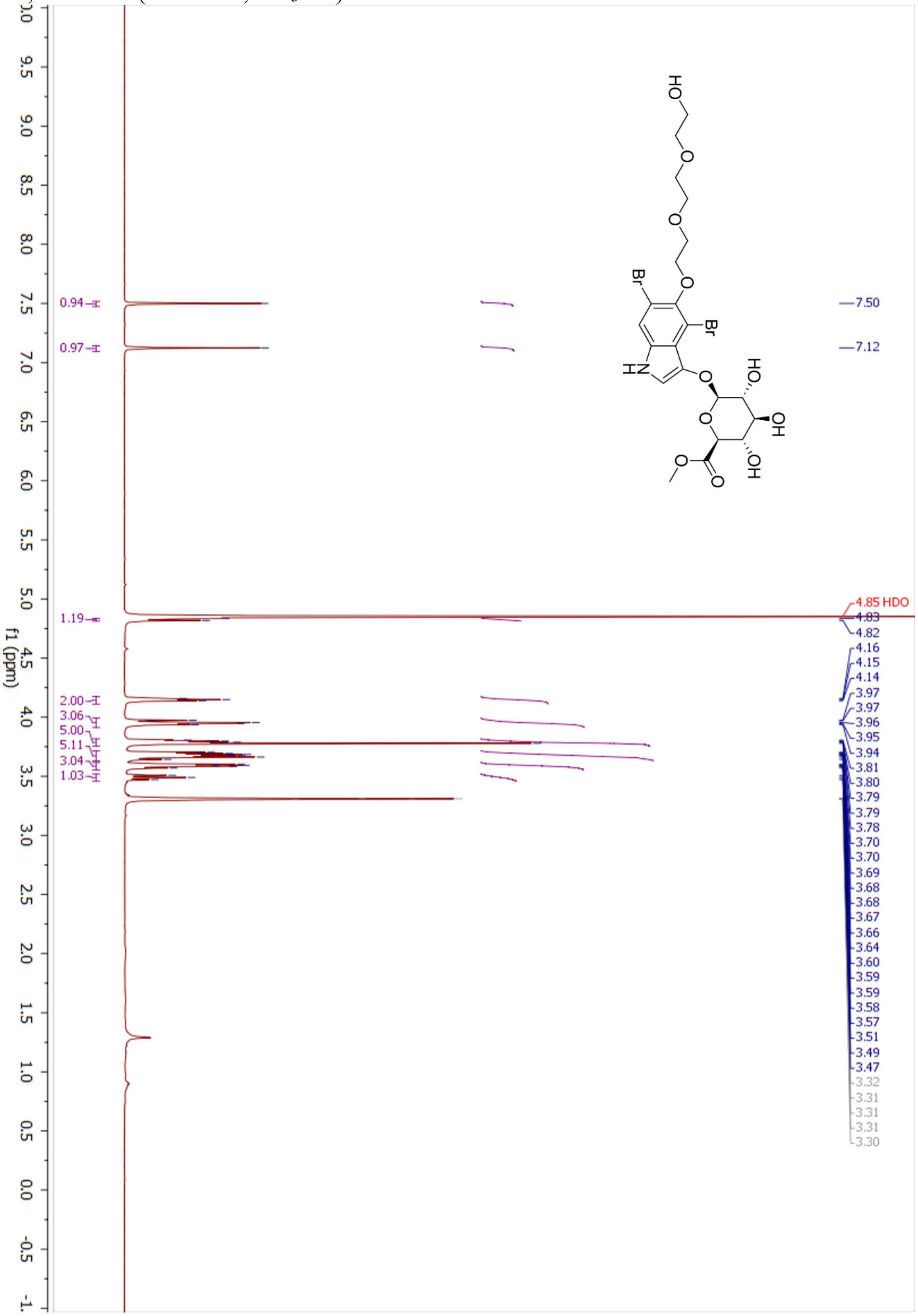

19, ^{13}C NMR (125 MHz, CD_3OD)

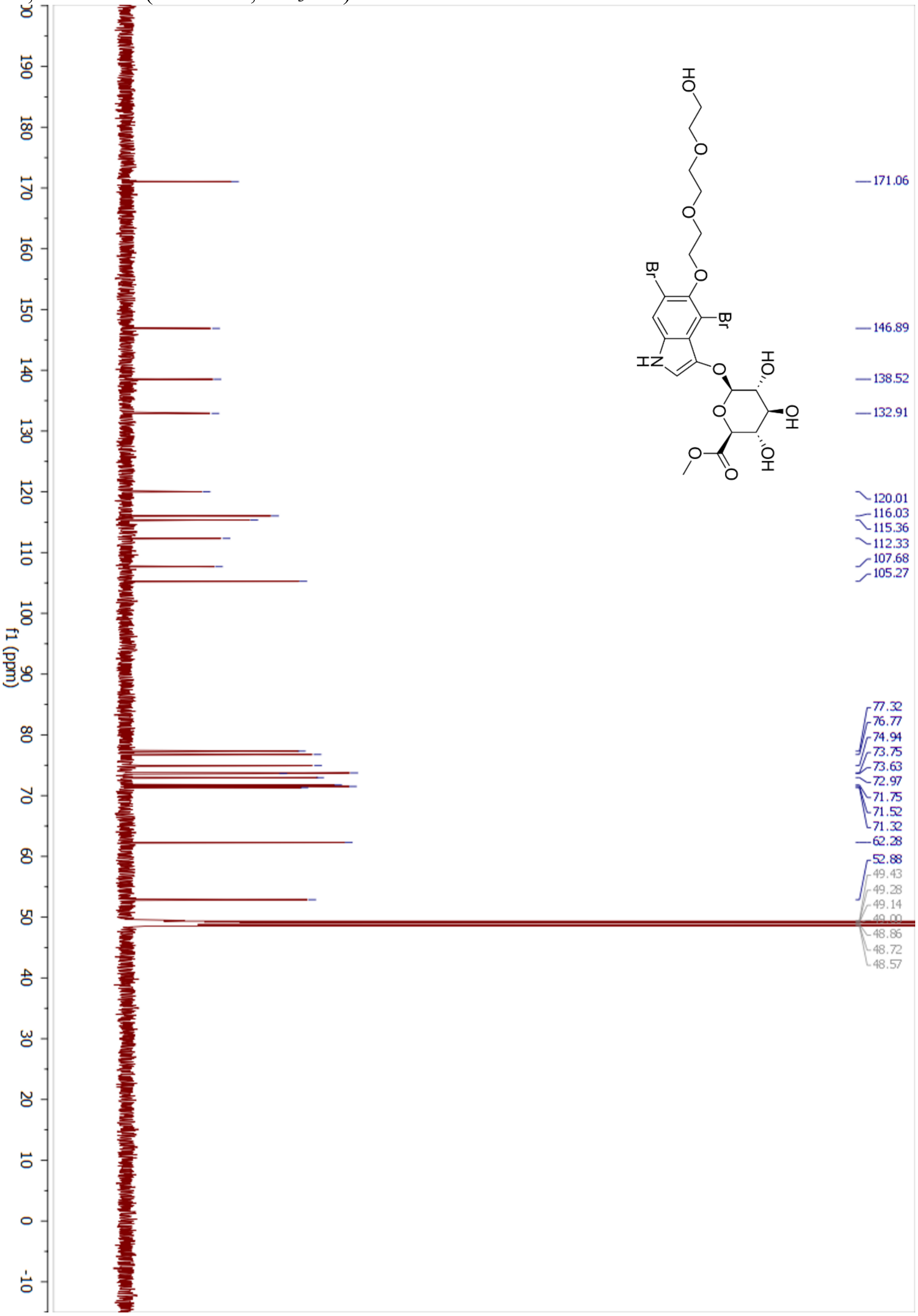

20, ^{1}H NMR (600 MHz, CD_3OD)

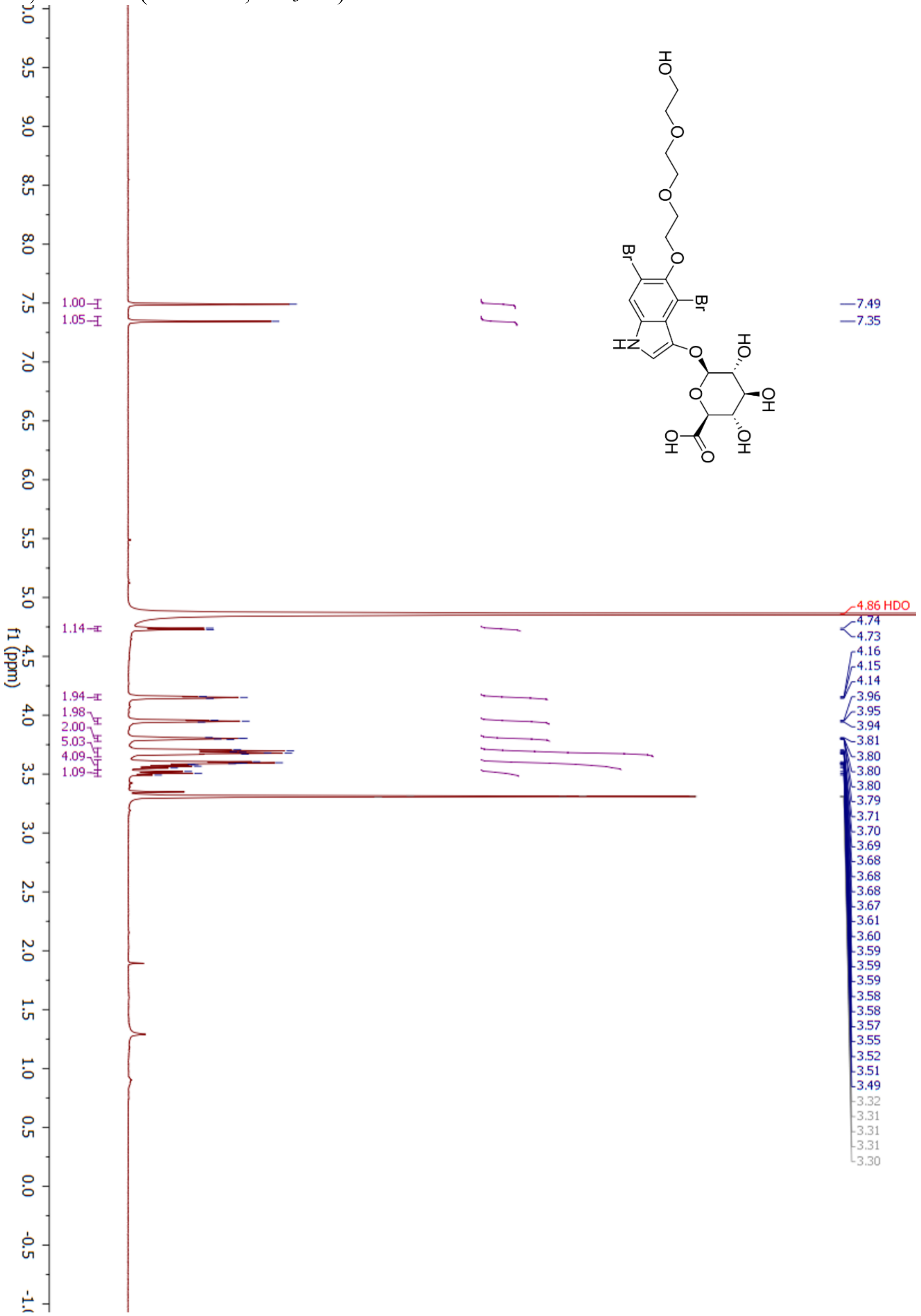

20, ^{13}C NMR (150 MHz, CD_3OD)

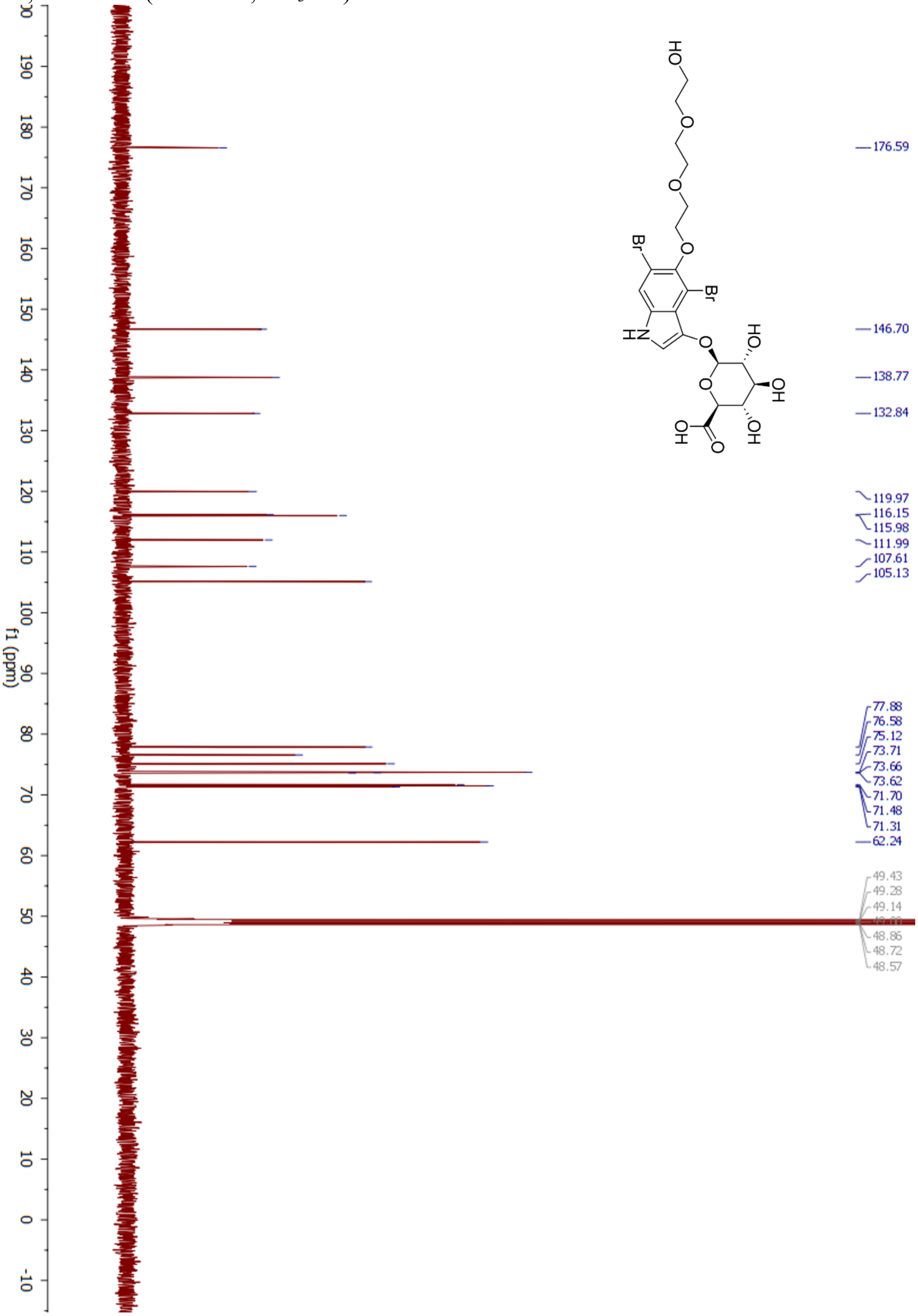

22-Br[5], ^{1}H NMR (700 MHz, $CDCl_3$)

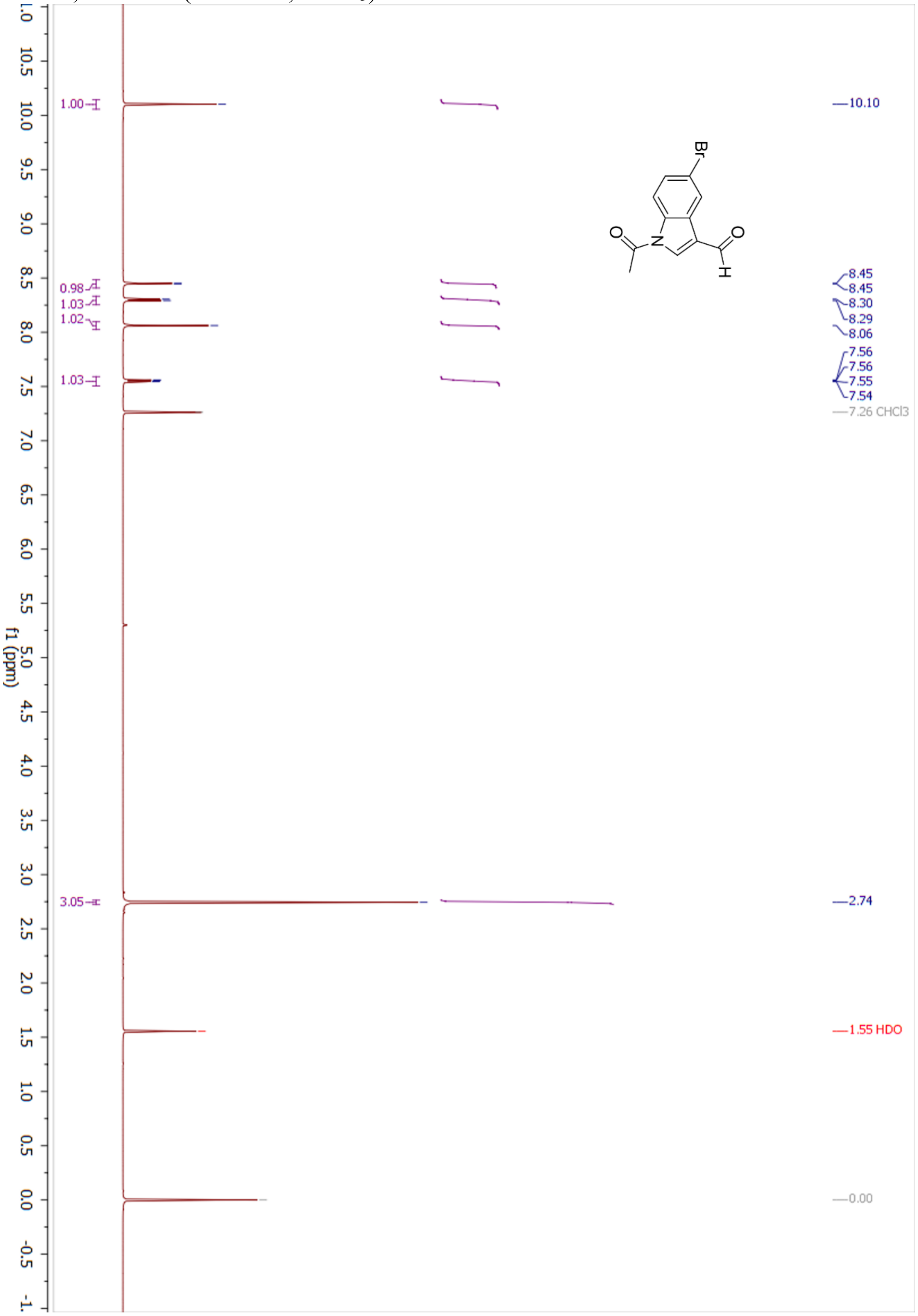

22-Br[5], ^{13}C NMR (175 MHz, $CDCl_3$)

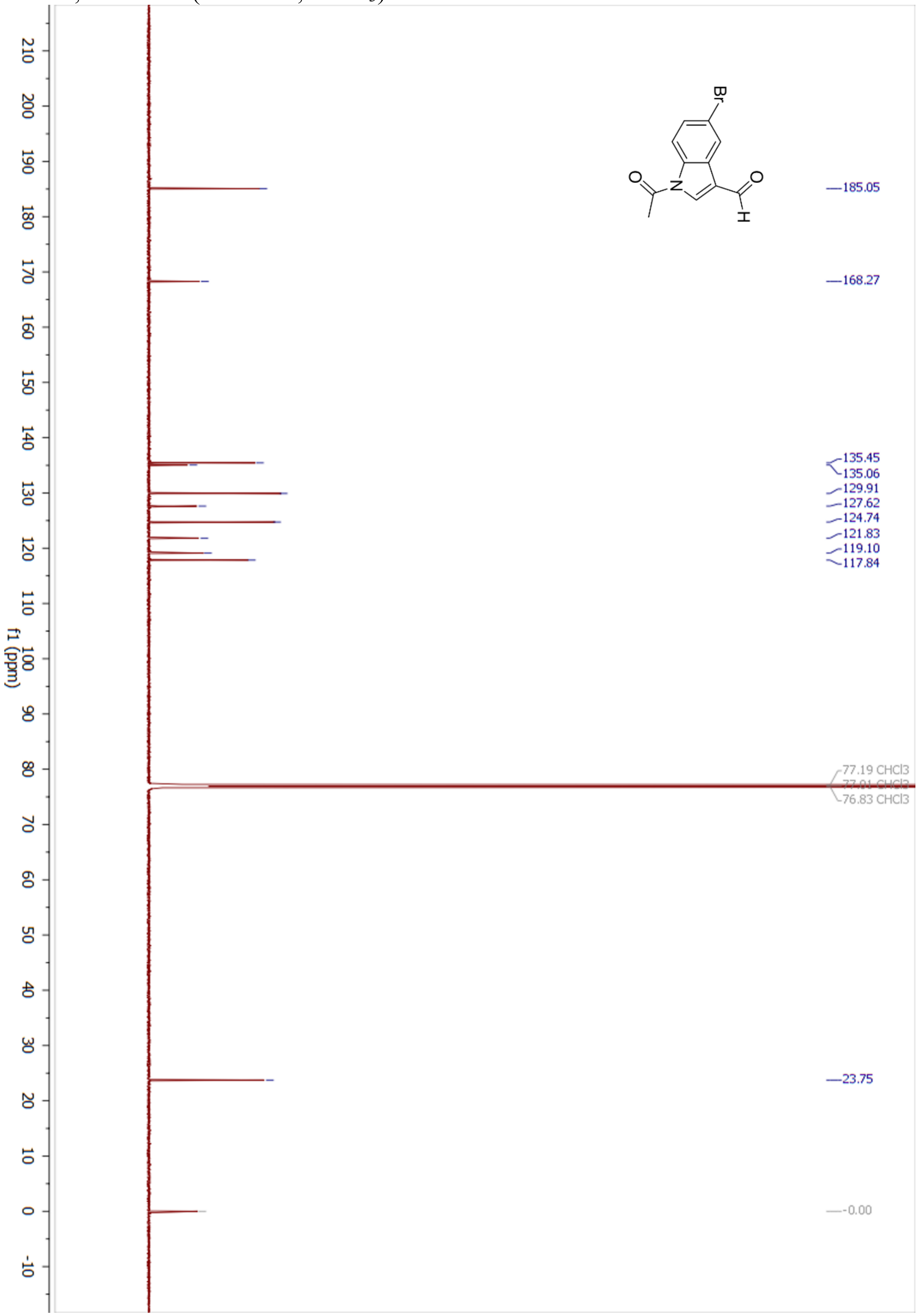

22-Br[6], ^{1}H NMR (700 MHz, $CDCl_3$)

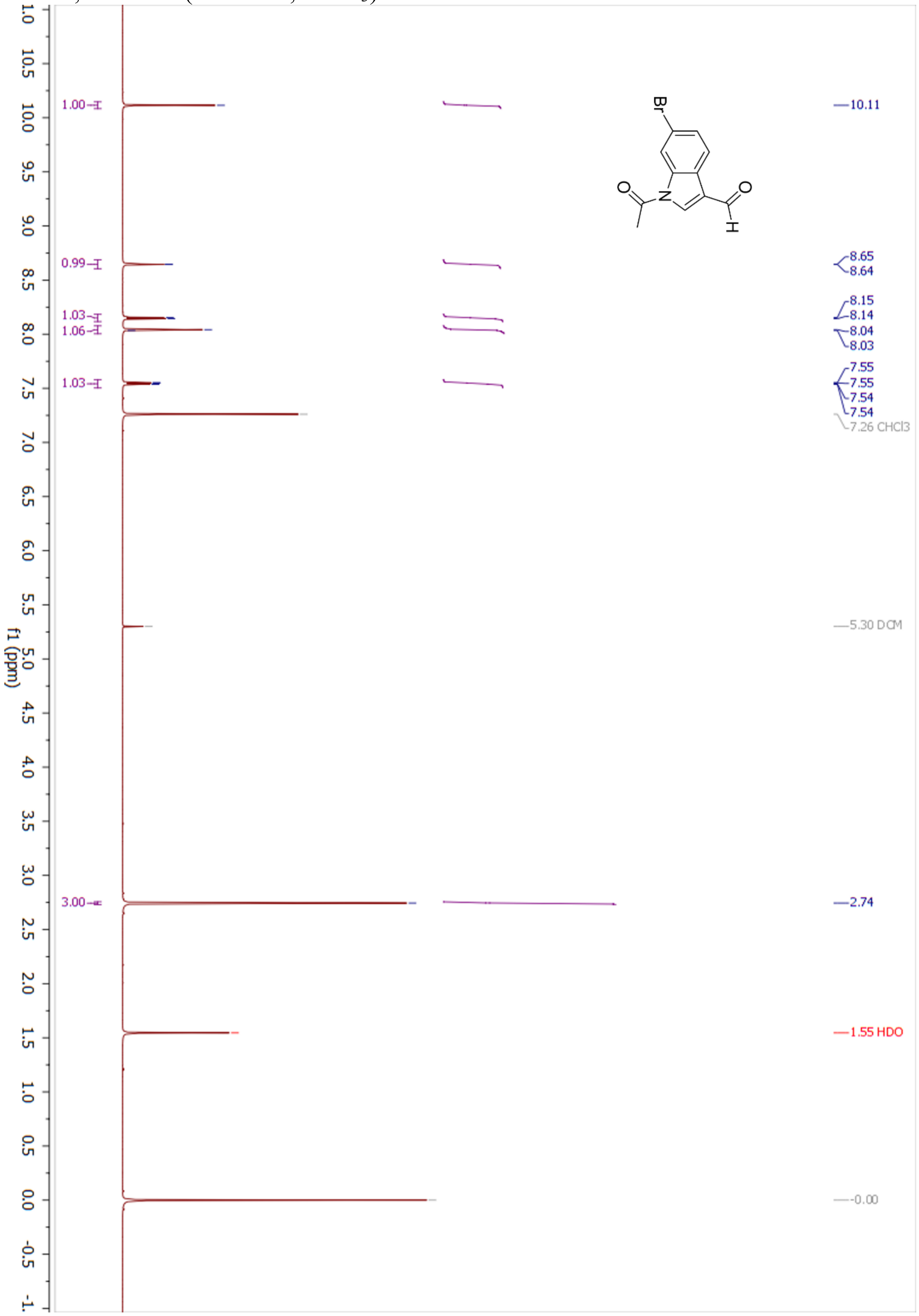

22-Br[6], ^{13}C NMR (175 MHz, $CDCl_3$)

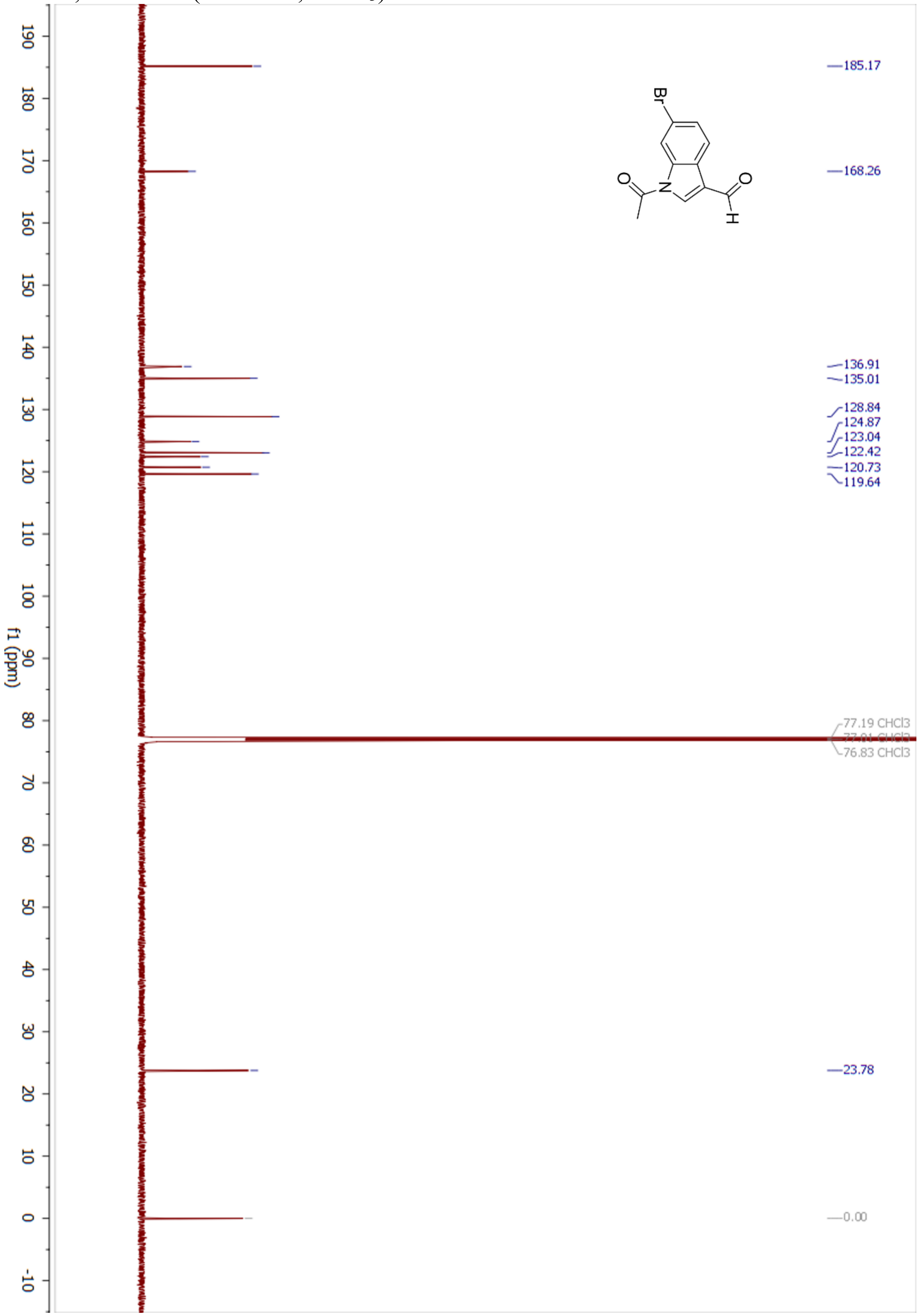

22-Br[7], ^1H NMR (700 MHz, $CDCl_3$)

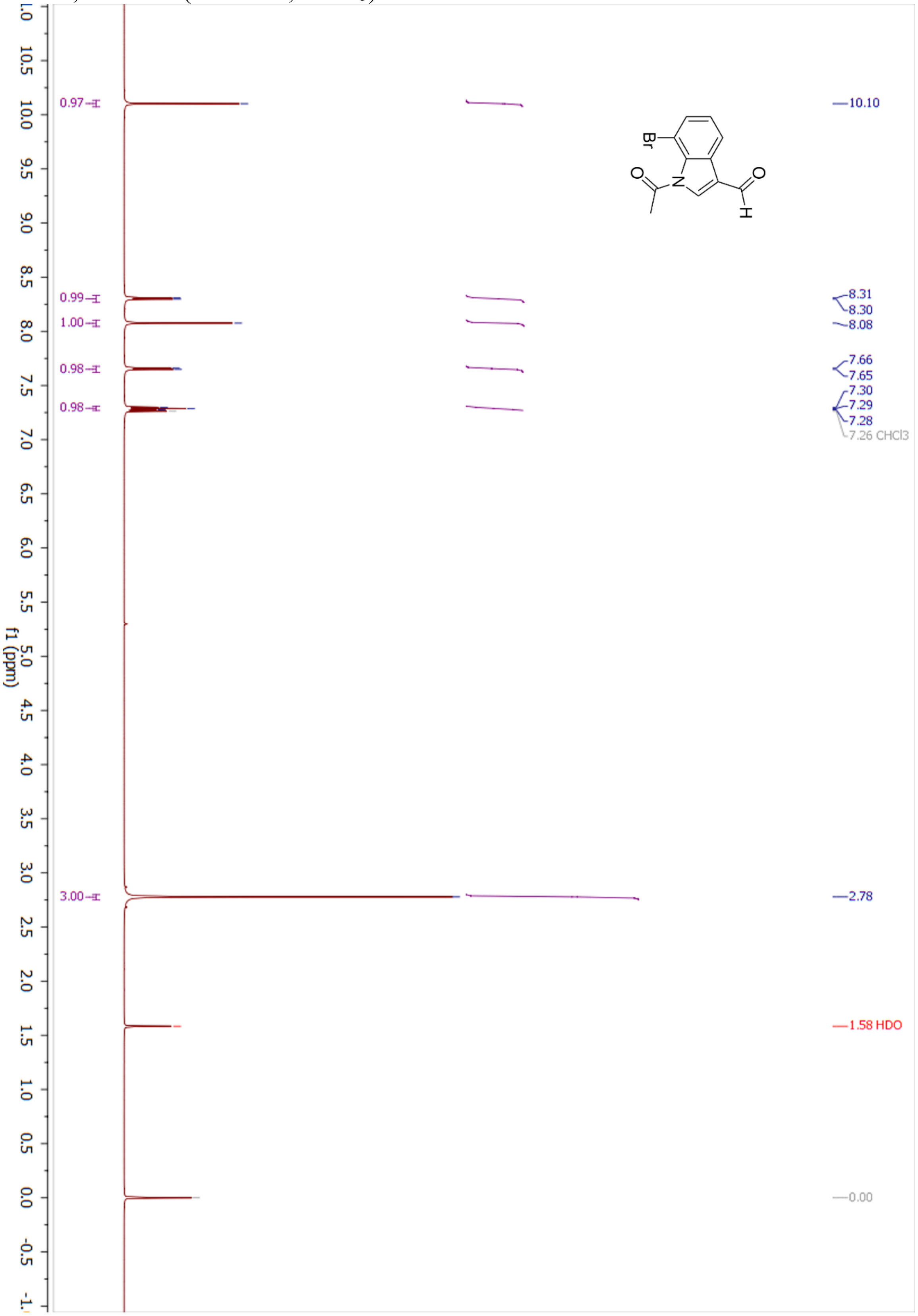

22-Br[7], ^{13}C NMR (175 MHz, $CDCl_3$)

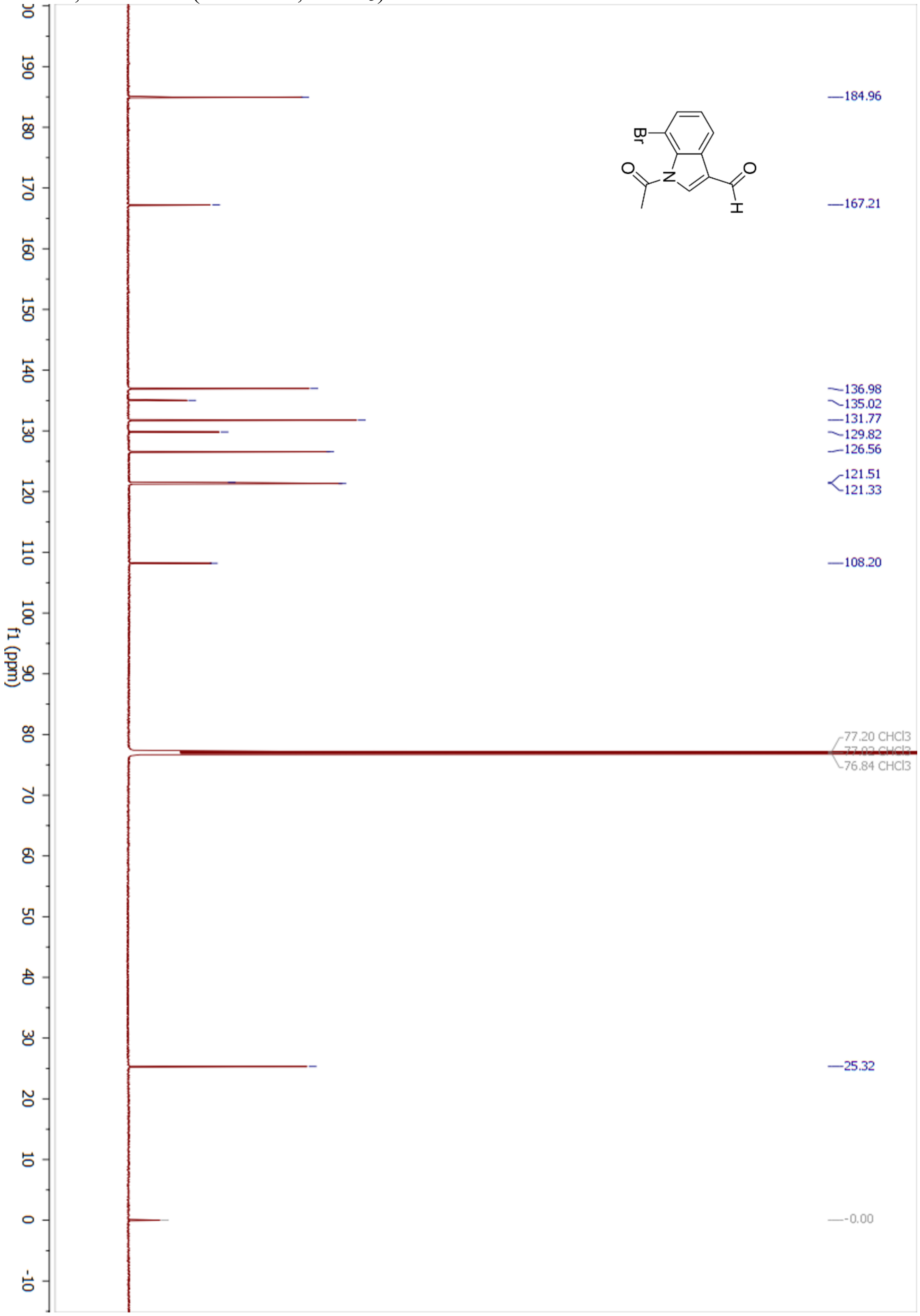

23-Br[5], ^{1}H NMR (700 MHz, $CDCl_3$)

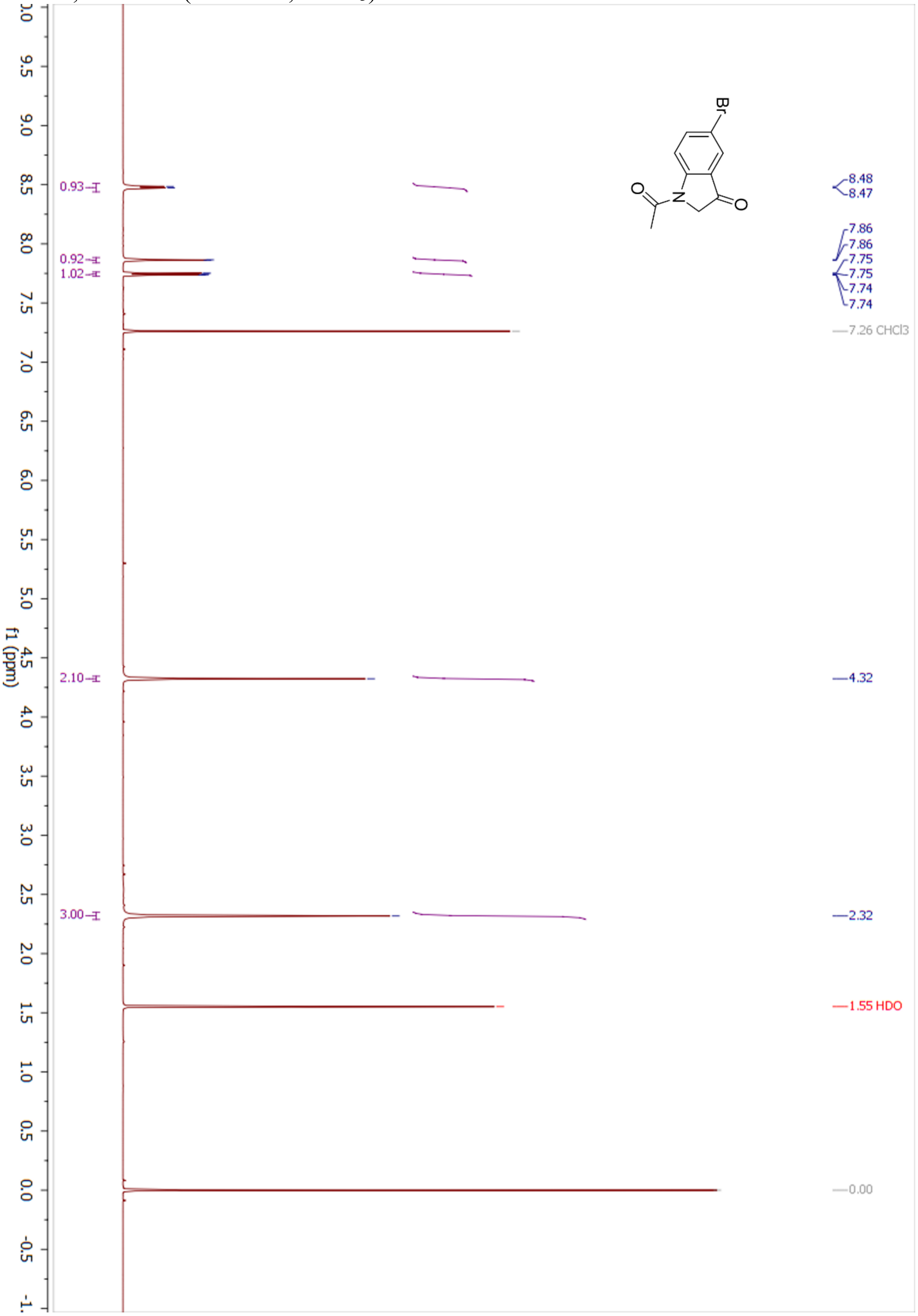

23-Br[5], ^{13}C NMR (175 MHz, $CDCl_3$)

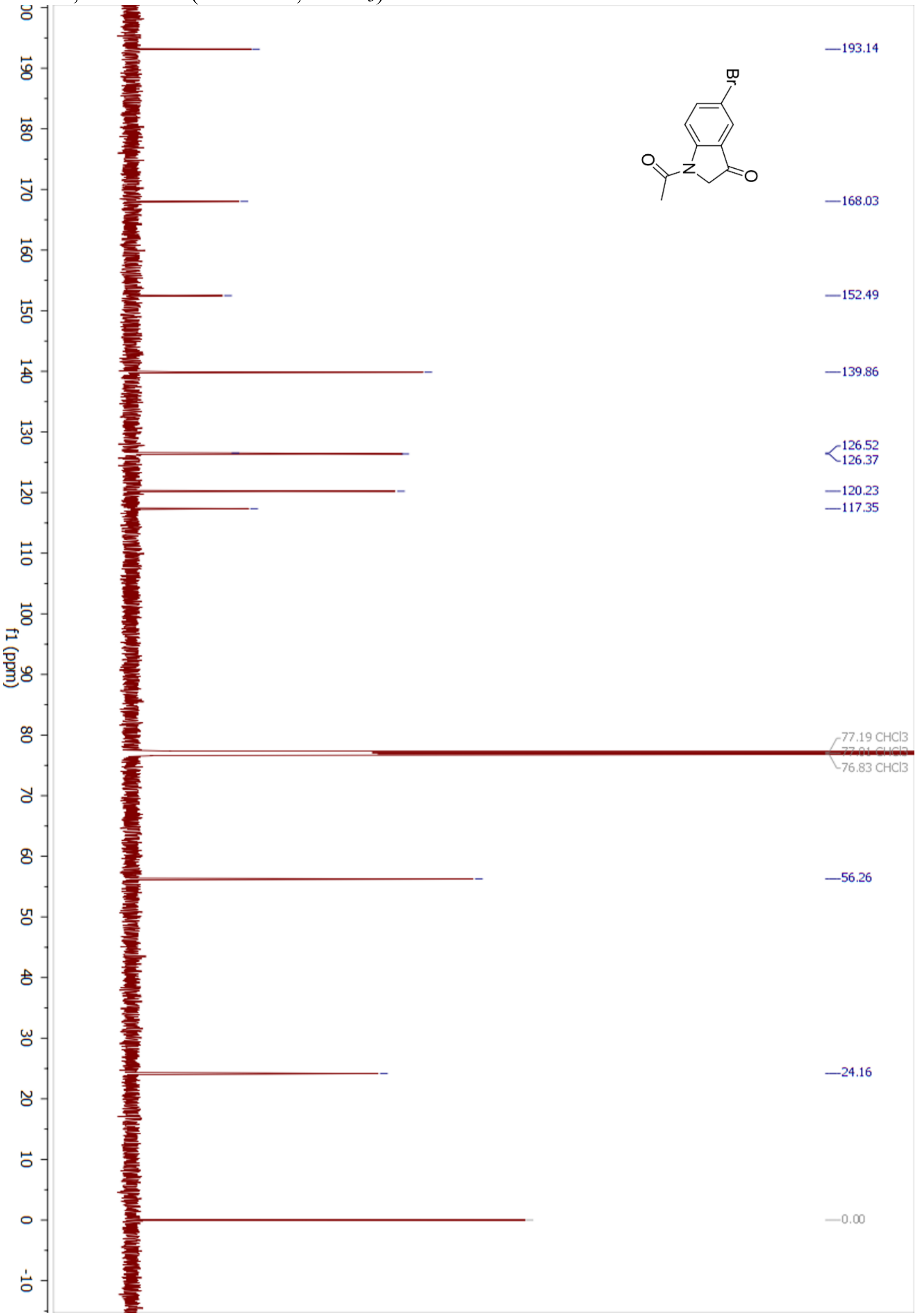

23-Br[6], ^{1}H NMR (700 MHz, $CDCl_3$)

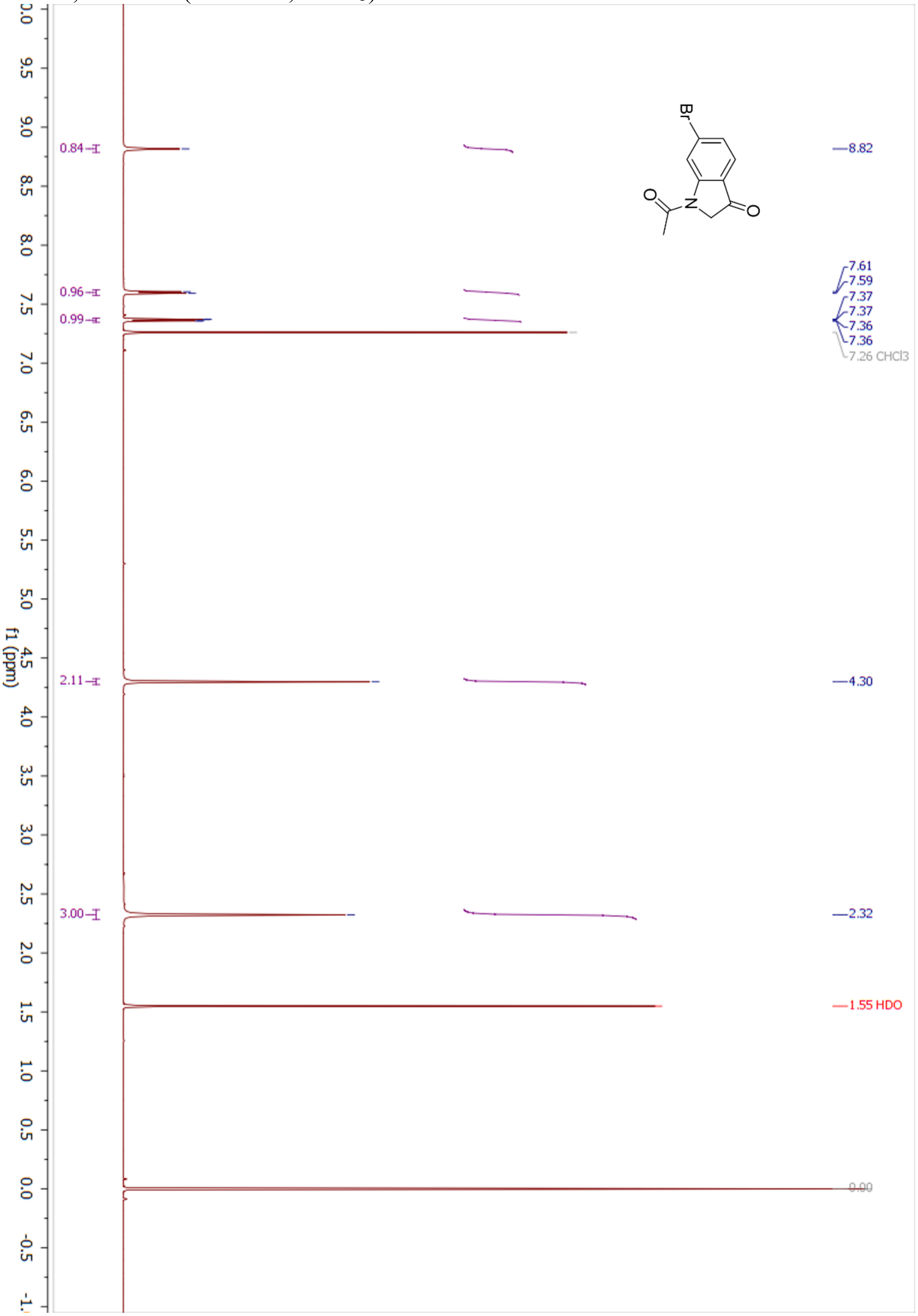

23-Br[6], ^{13}C NMR (175 MHz, $CDCl_3$)

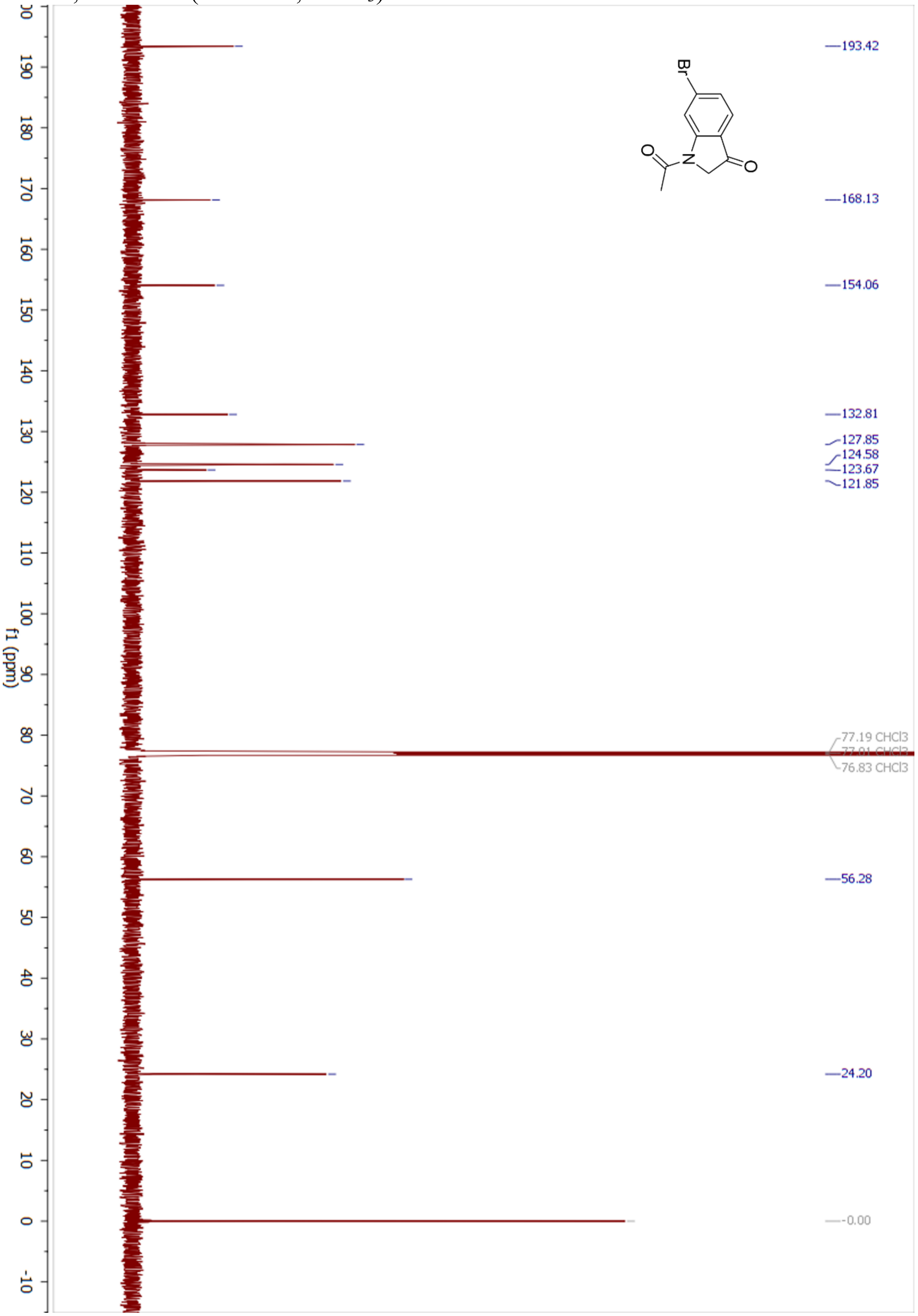

23-Br[7], ^{1}H NMR (700 MHz, $CDCl_3$)

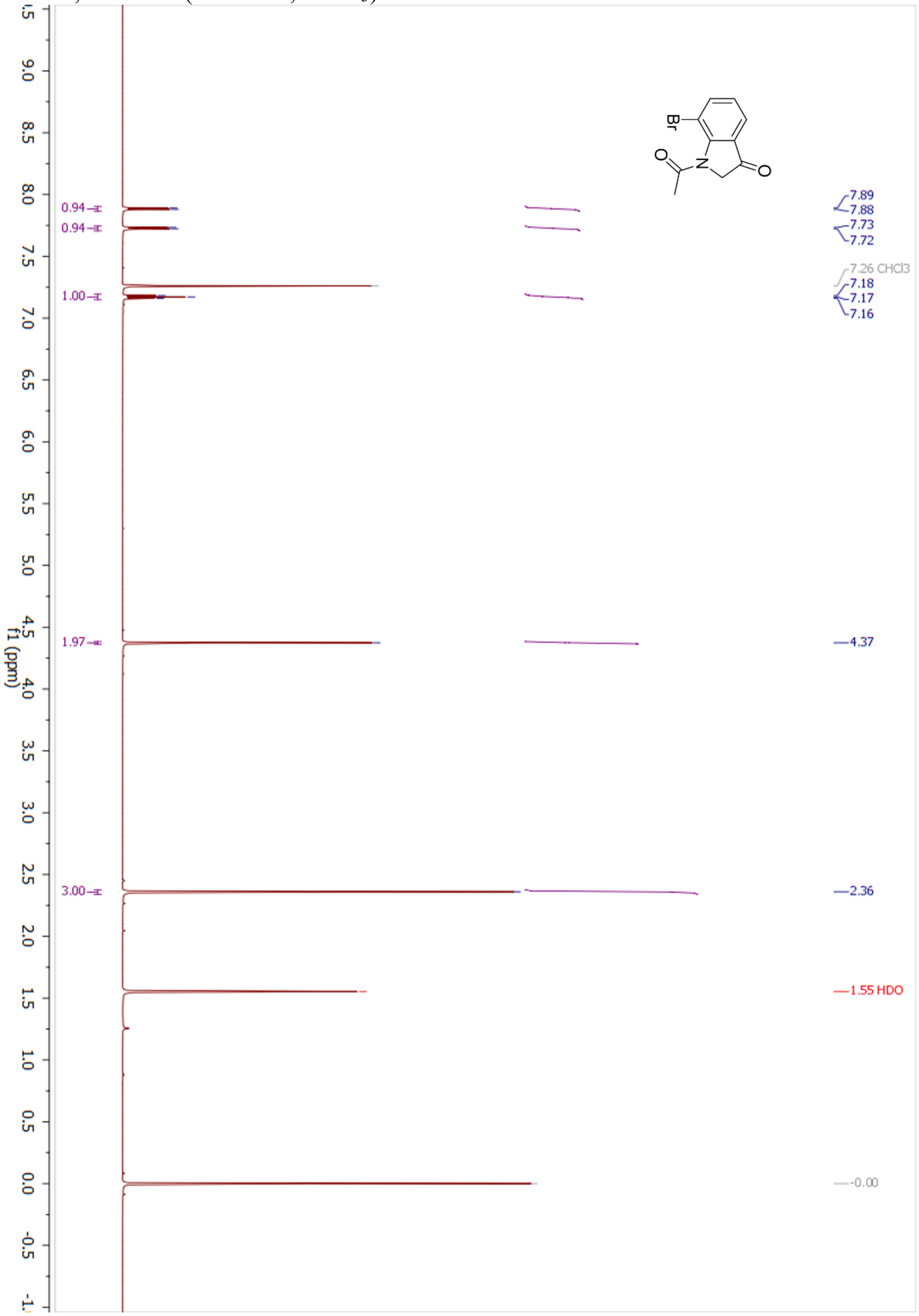

23-Br[7], ^{13}C NMR (175 MHz, $CDCl_3$)

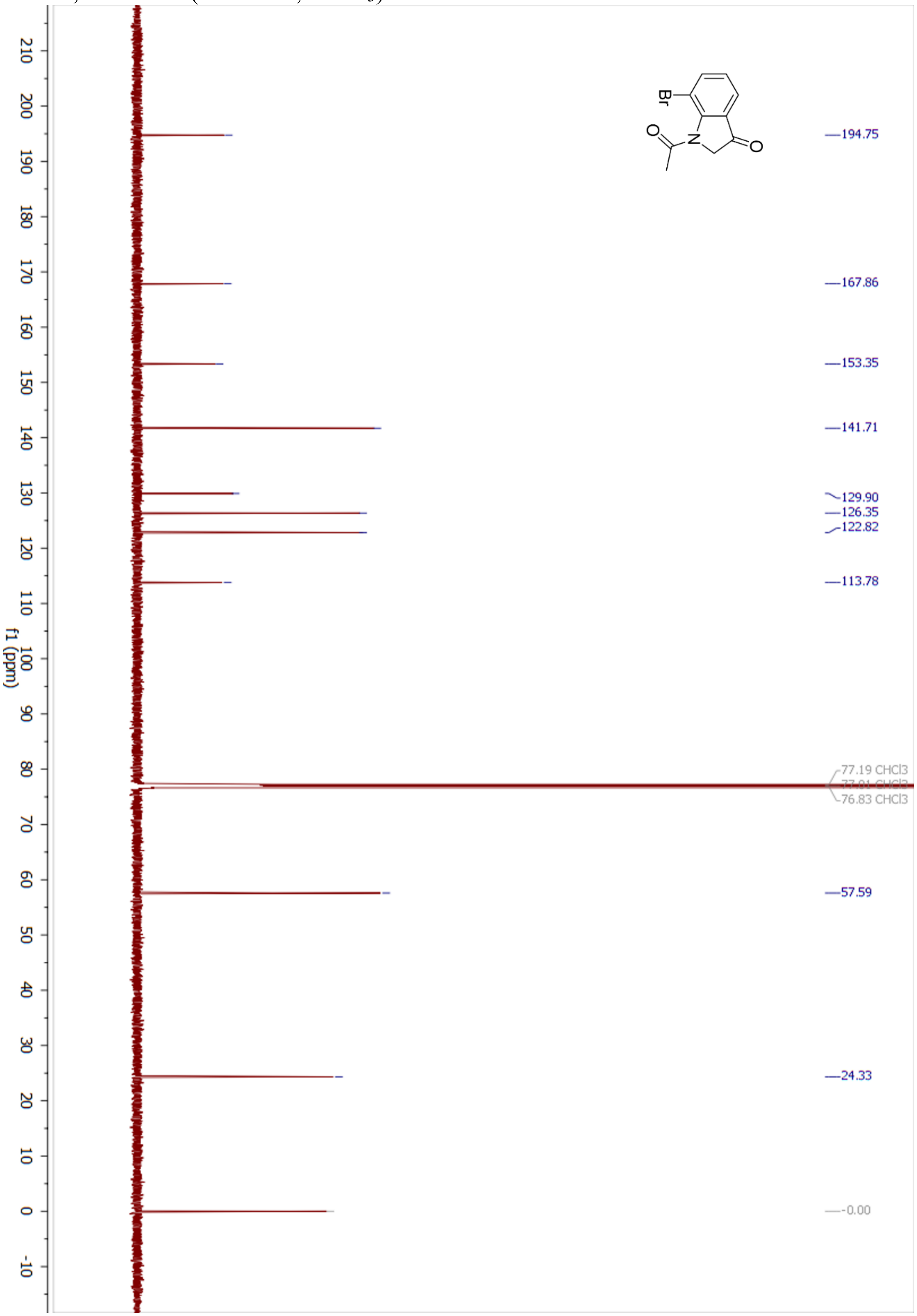

24-Br[5], ^{1}H NMR (500 MHz, $CDCl_3$)

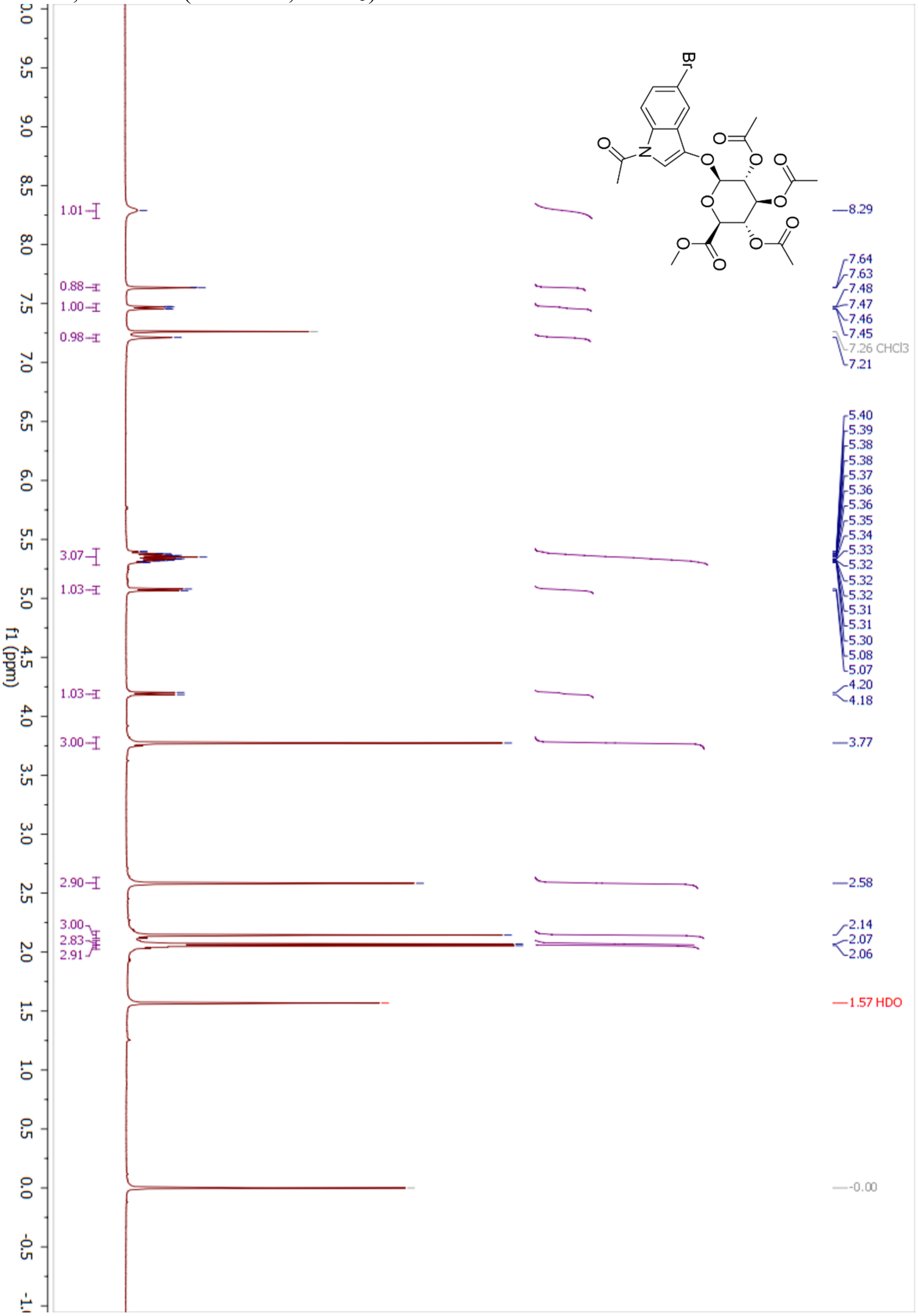

24-Br[5], ^{13}C NMR (175 MHz, $CDCl_3$)

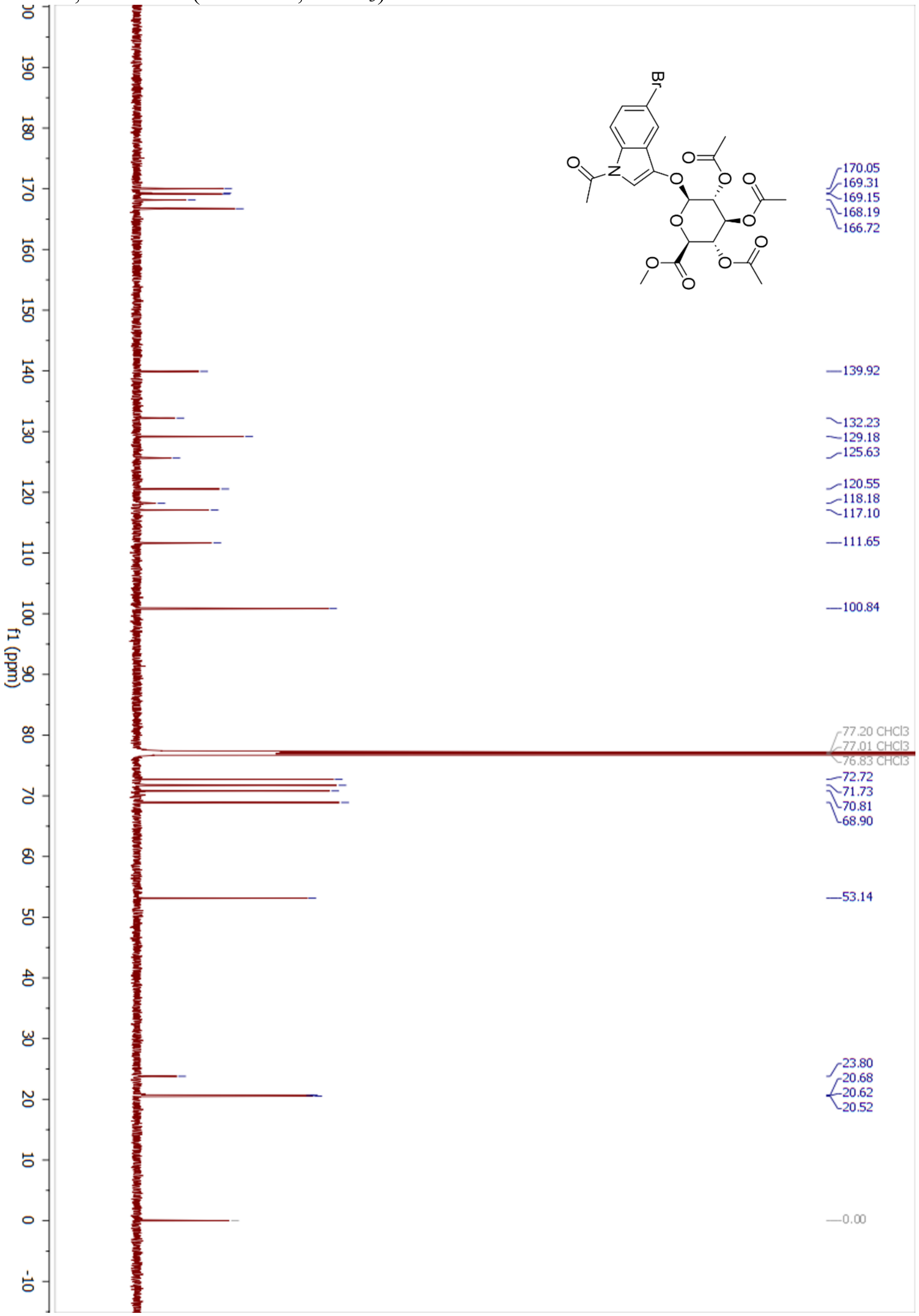

24-Br[6], ^{1}H NMR (700 MHz, $CDCl_3$)

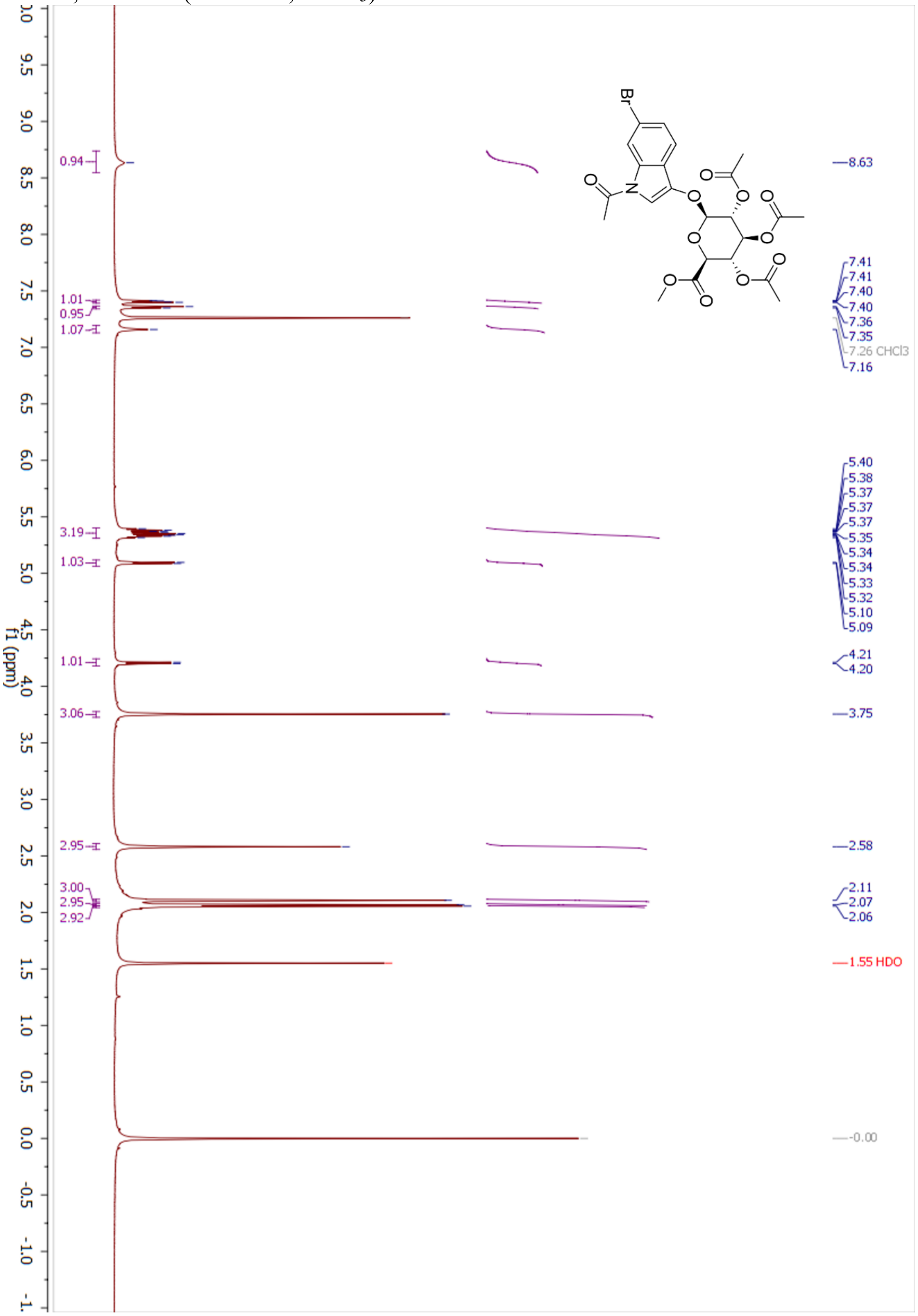

24-Br^6, ^{13}C NMR (175 MHz, $CDCl_3$)

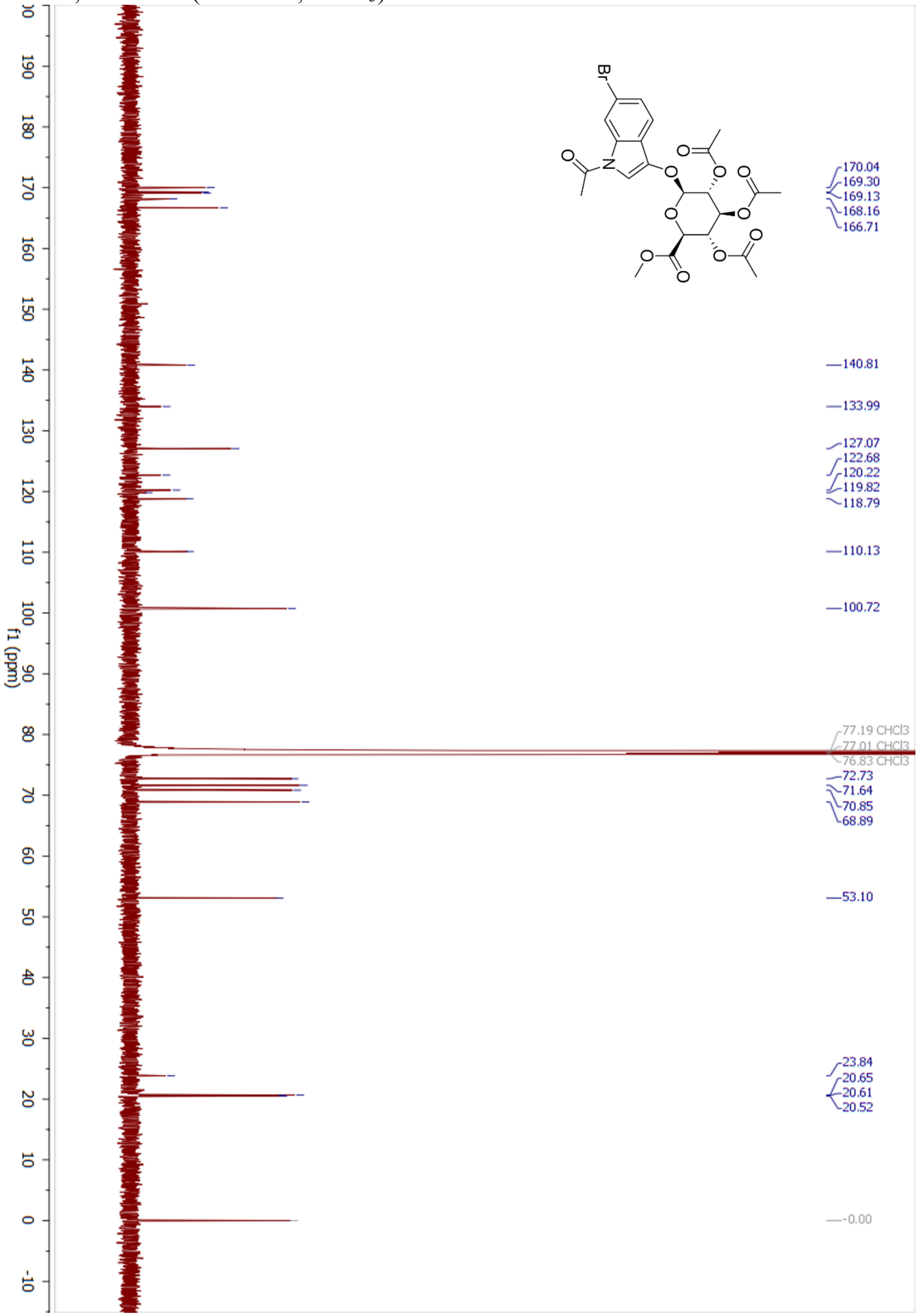

24-Br[7], ^{1}H NMR (700 MHz, $CDCl_3$)

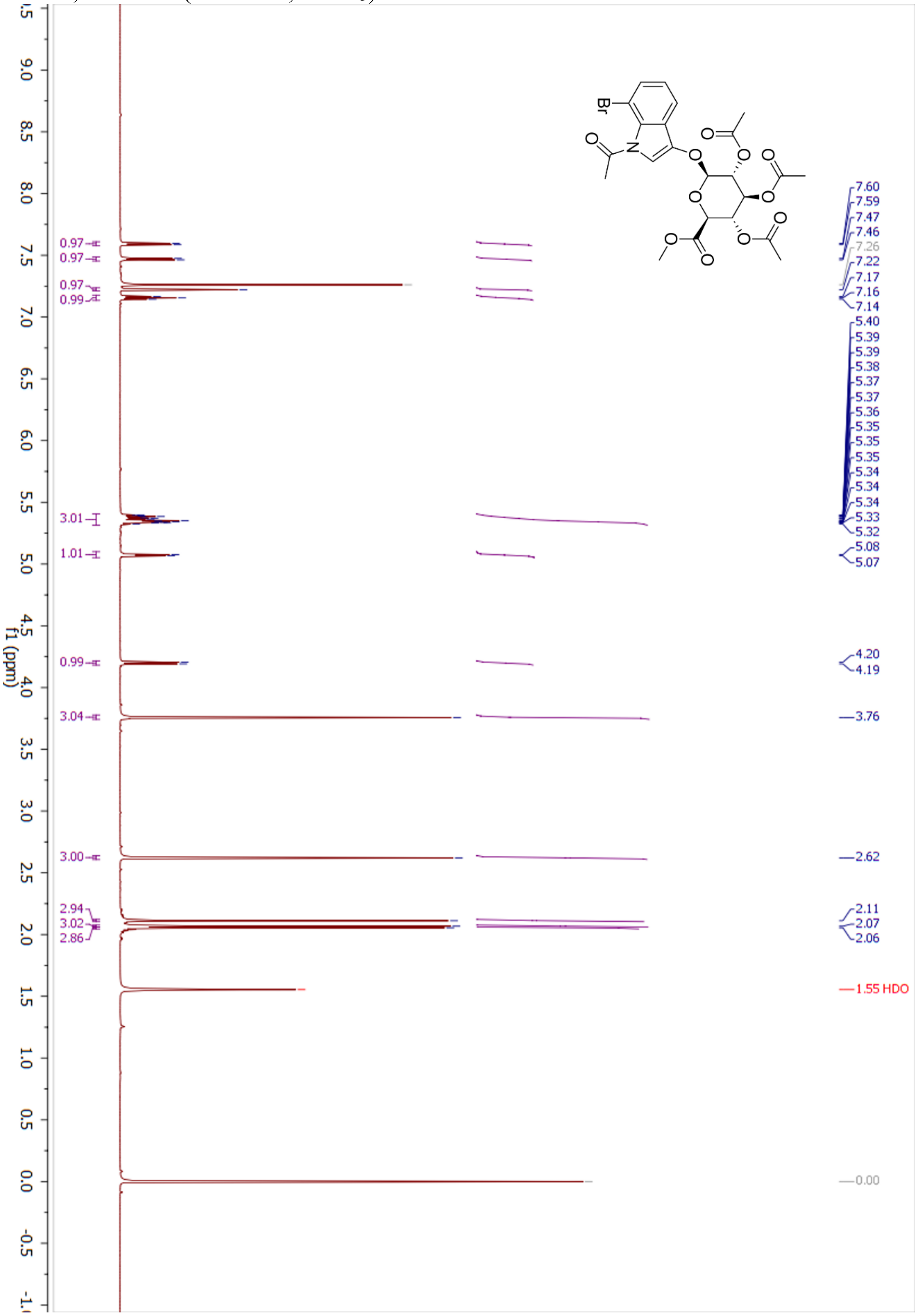

24-Br[7], ^{13}C NMR (175 MHz, $CDCl_3$)

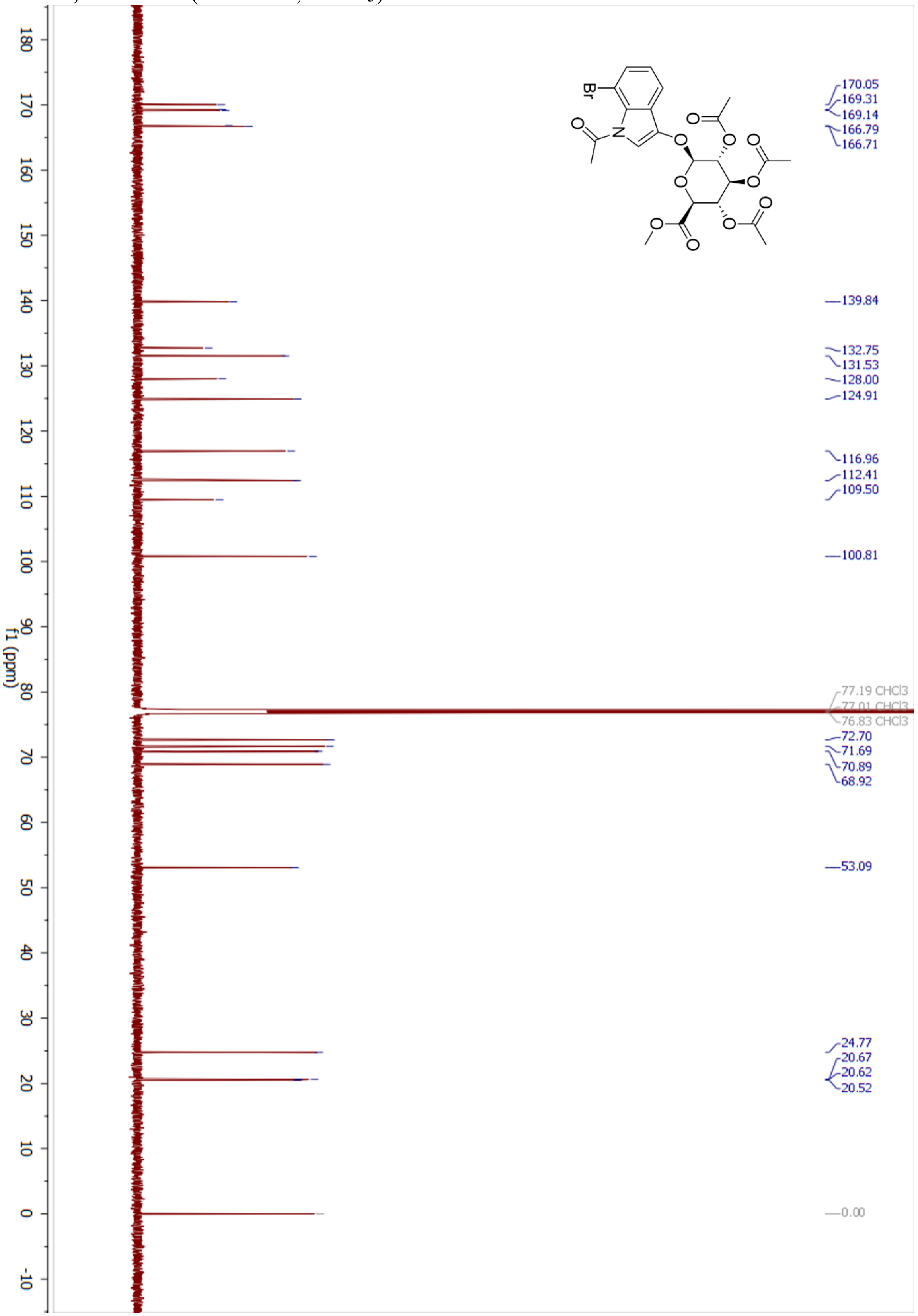

25, ^{1}H NMR (700 MHz, CD_3OD)

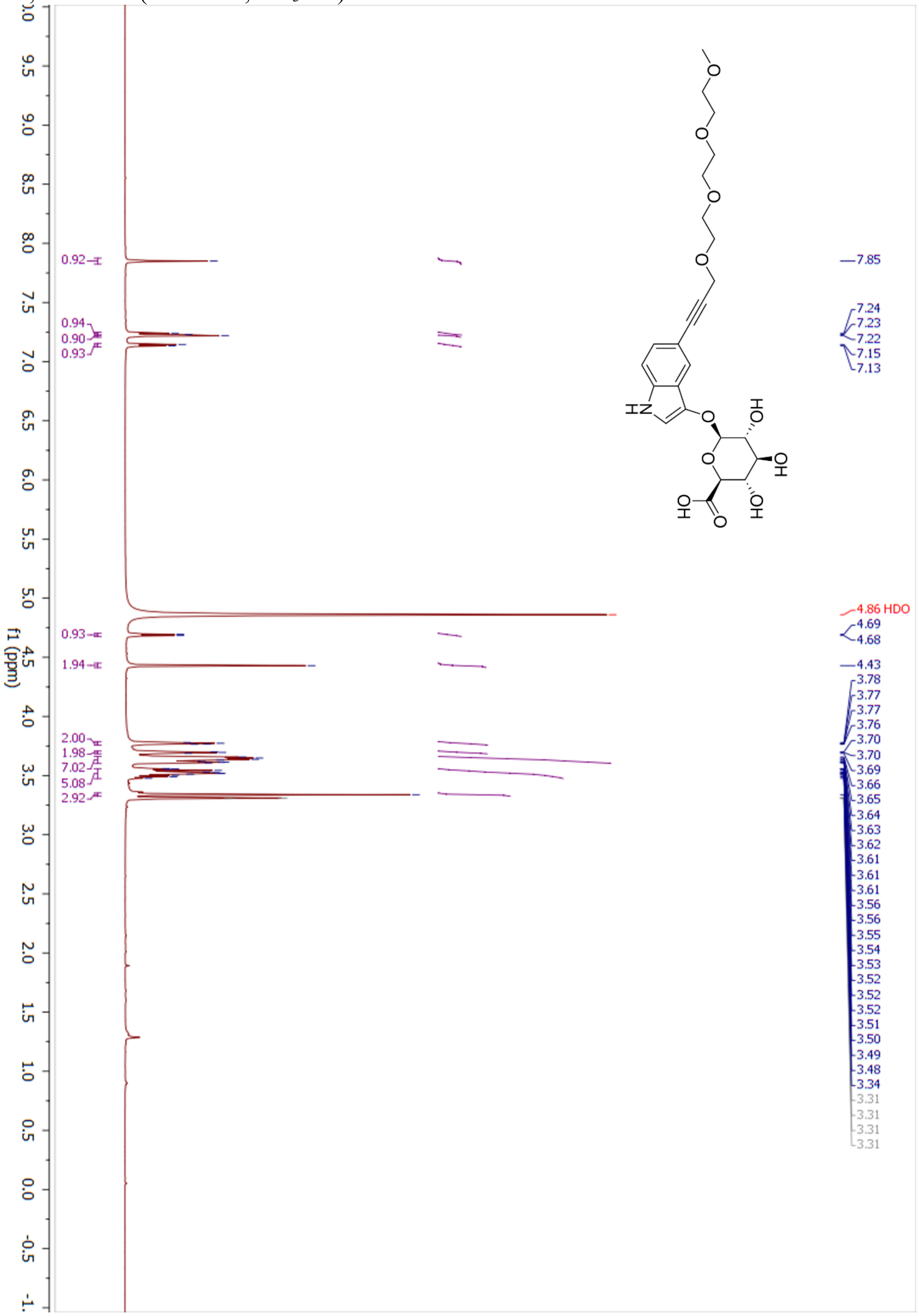

25, ^{13}C NMR (175 MHz, CD_3OD)

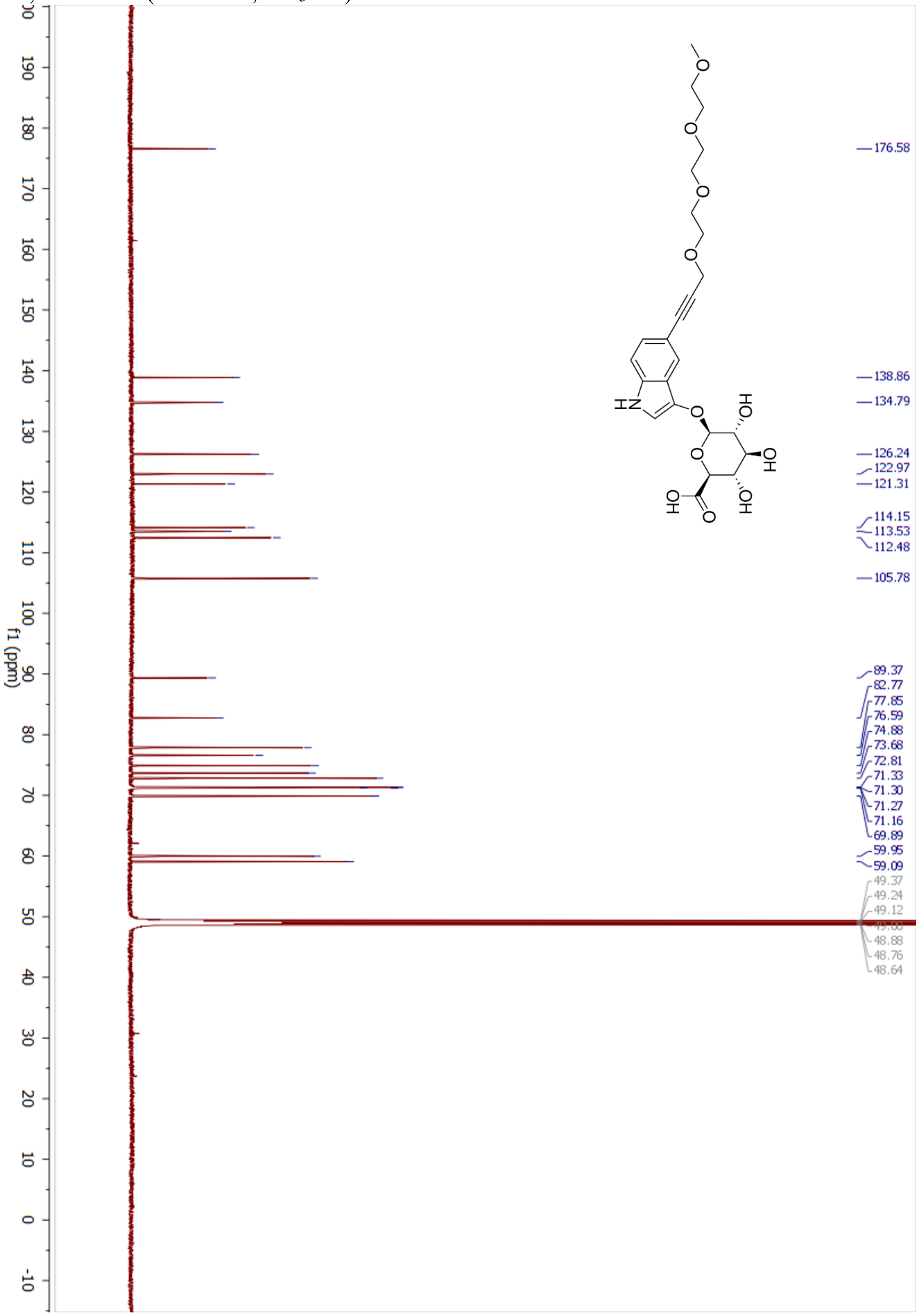

26, ^{1}H NMR (700 MHz, CD_3OD)

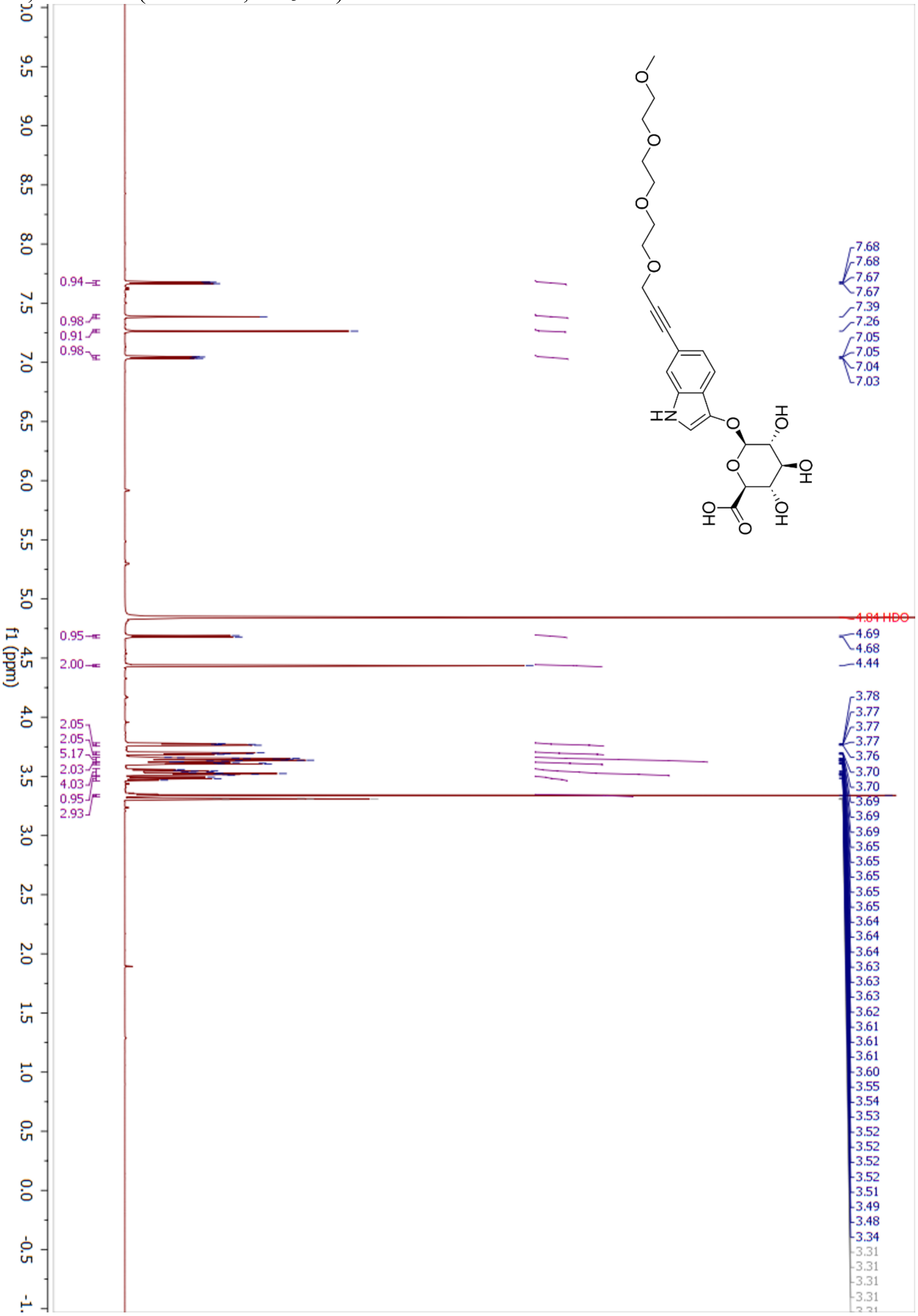

26, ^{13}C NMR (175 MHz, CD_3OD)

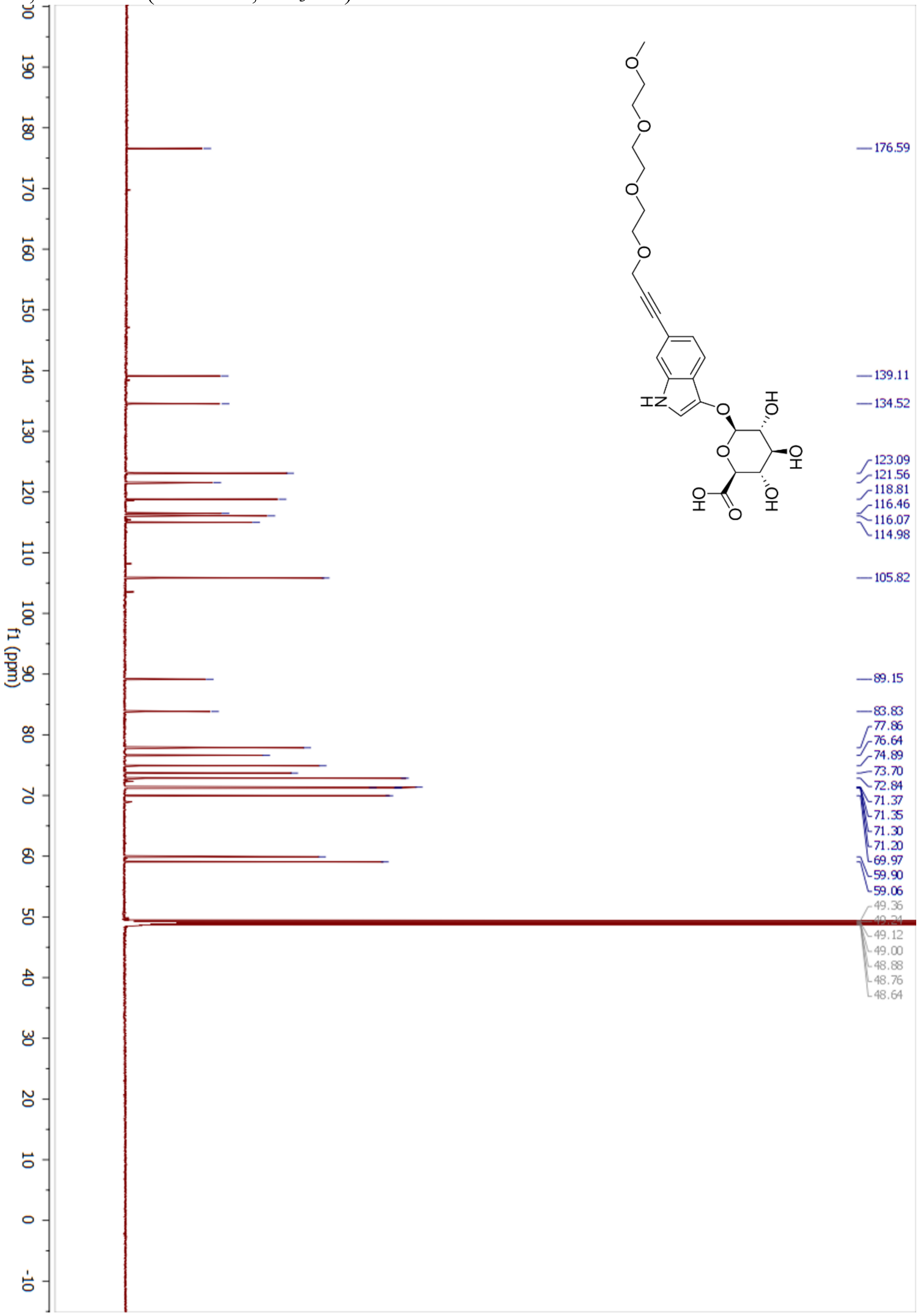

27, ^{1}H NMR (700 MHz, CD_3OD)

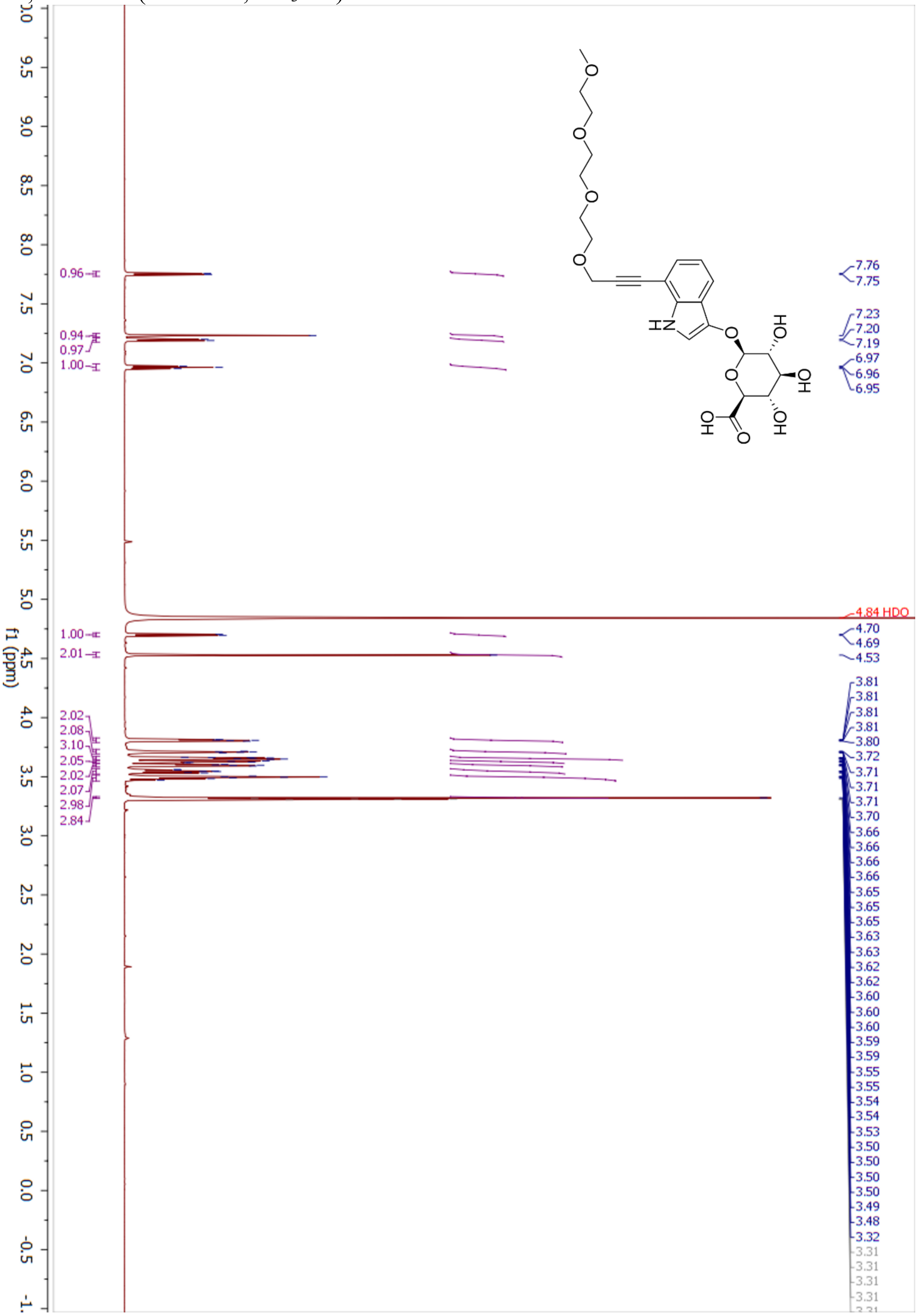

27, ^{13}C NMR (175 MHz, CD_3OD)

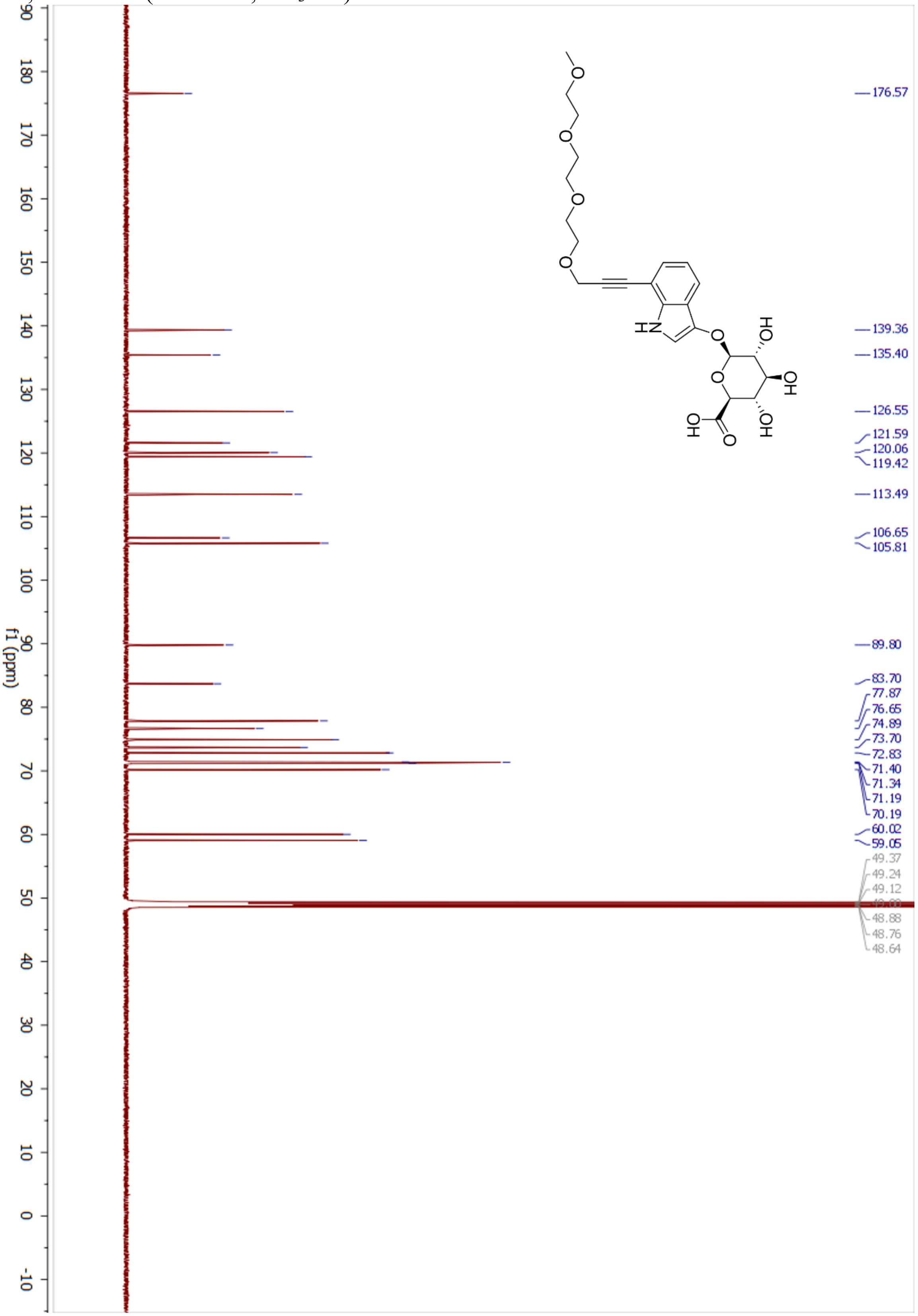

Ind(25)$_2$, ^{1}H NMR (700 MHz, $CDCl_3$)

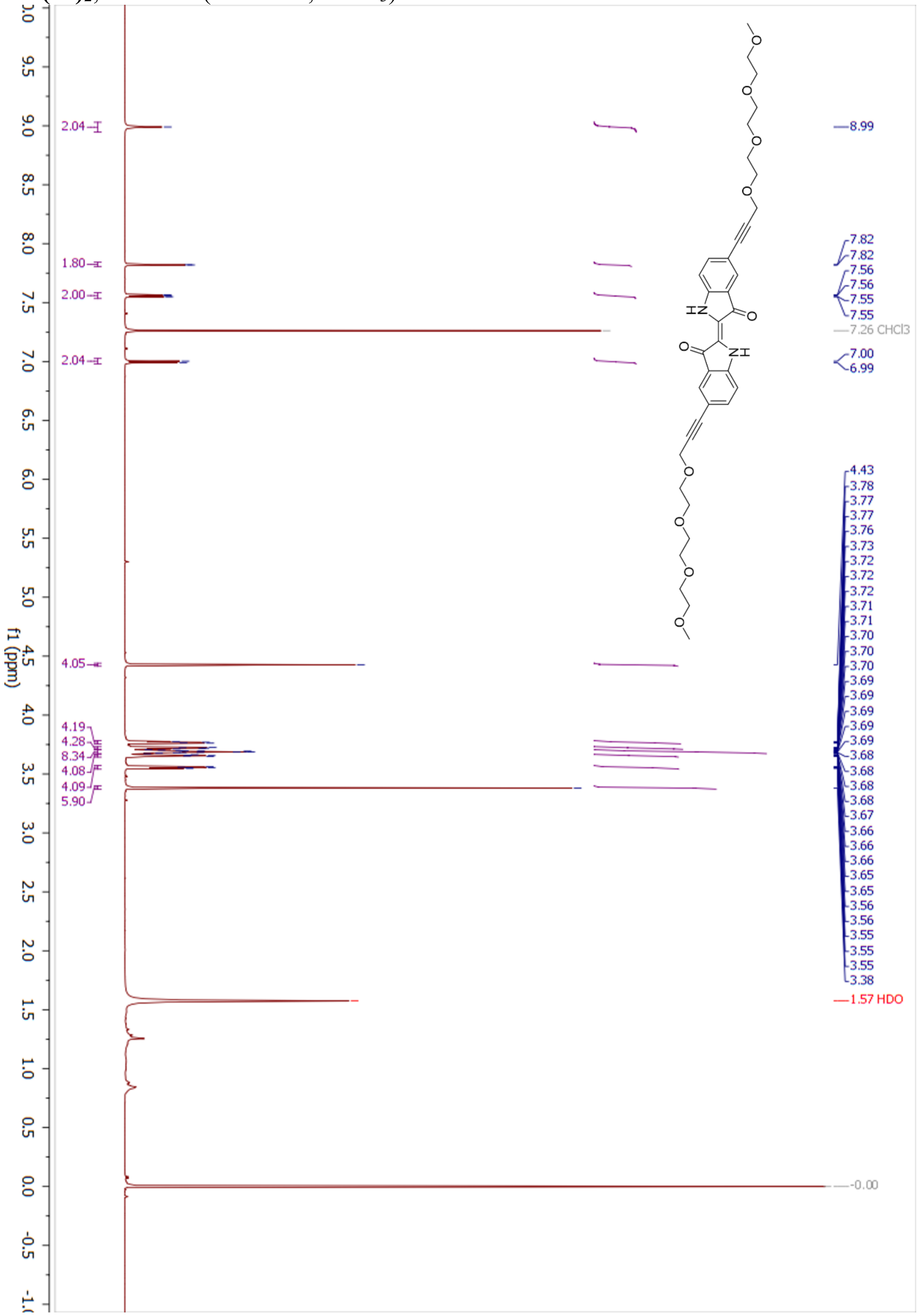

Ind(25)₂, ^{13}C NMR (175 MHz, $CDCl_3$)

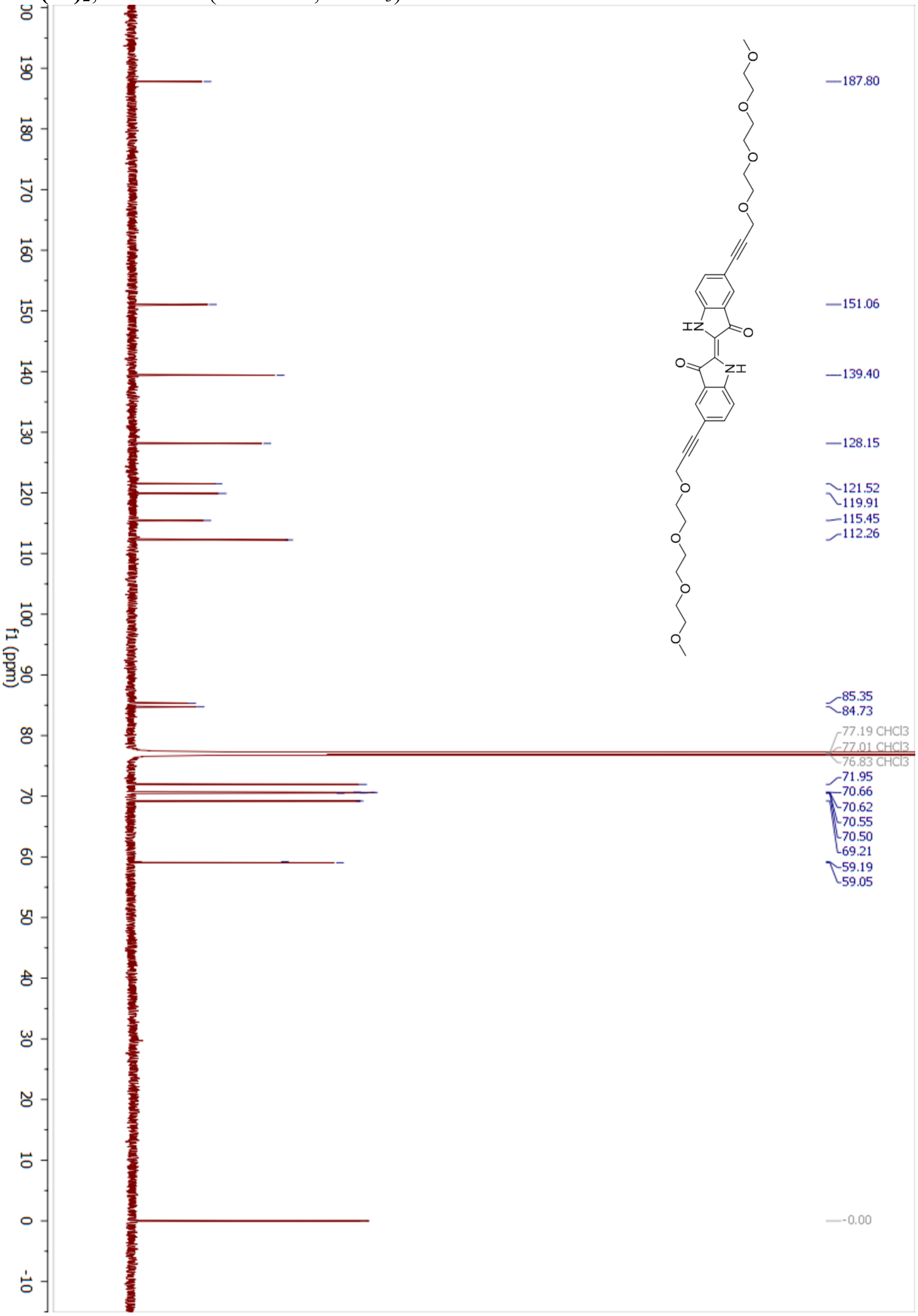

Ind(26)$_2$, ^{1}H NMR (700 MHz, $CDCl_3$)

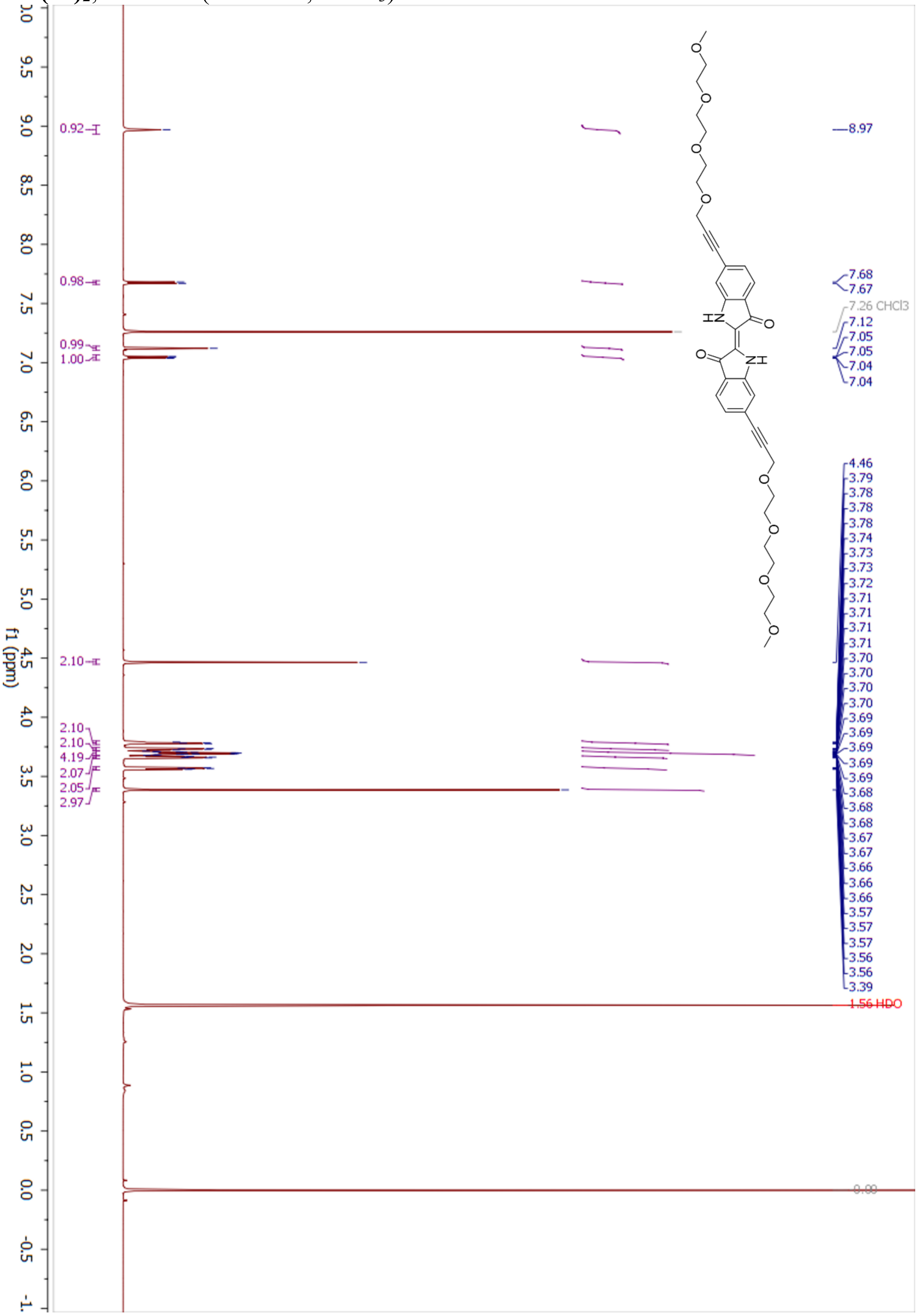

Ind(26)$_2$, ^{13}C NMR (175 MHz, $CDCl_3$)

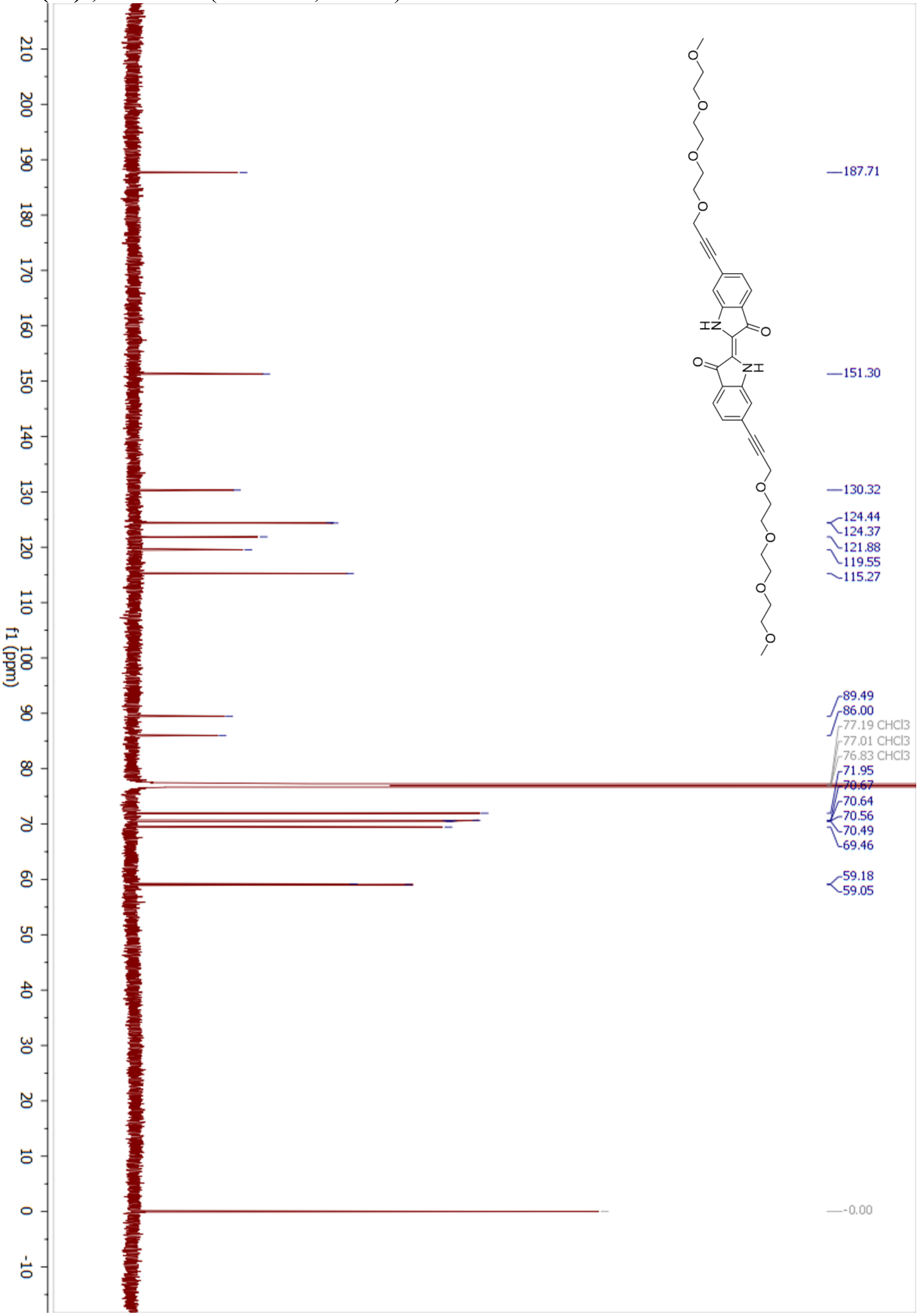

Ind(27)₂, ^{1}H NMR (700 MHz, $CDCl_3$)

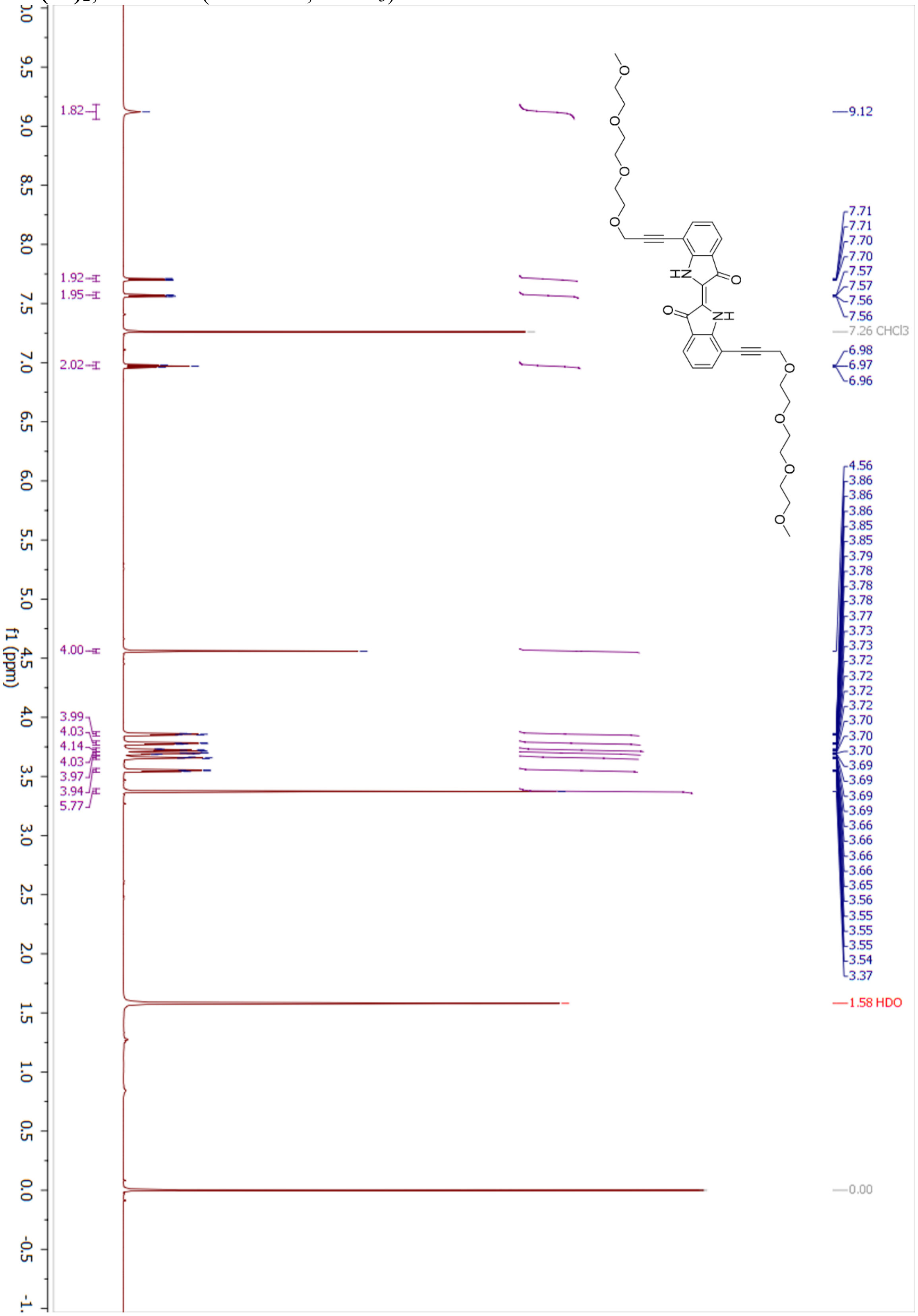

Ind(27)₂, ^{13}C NMR (175 MHz, $CDCl_3$)

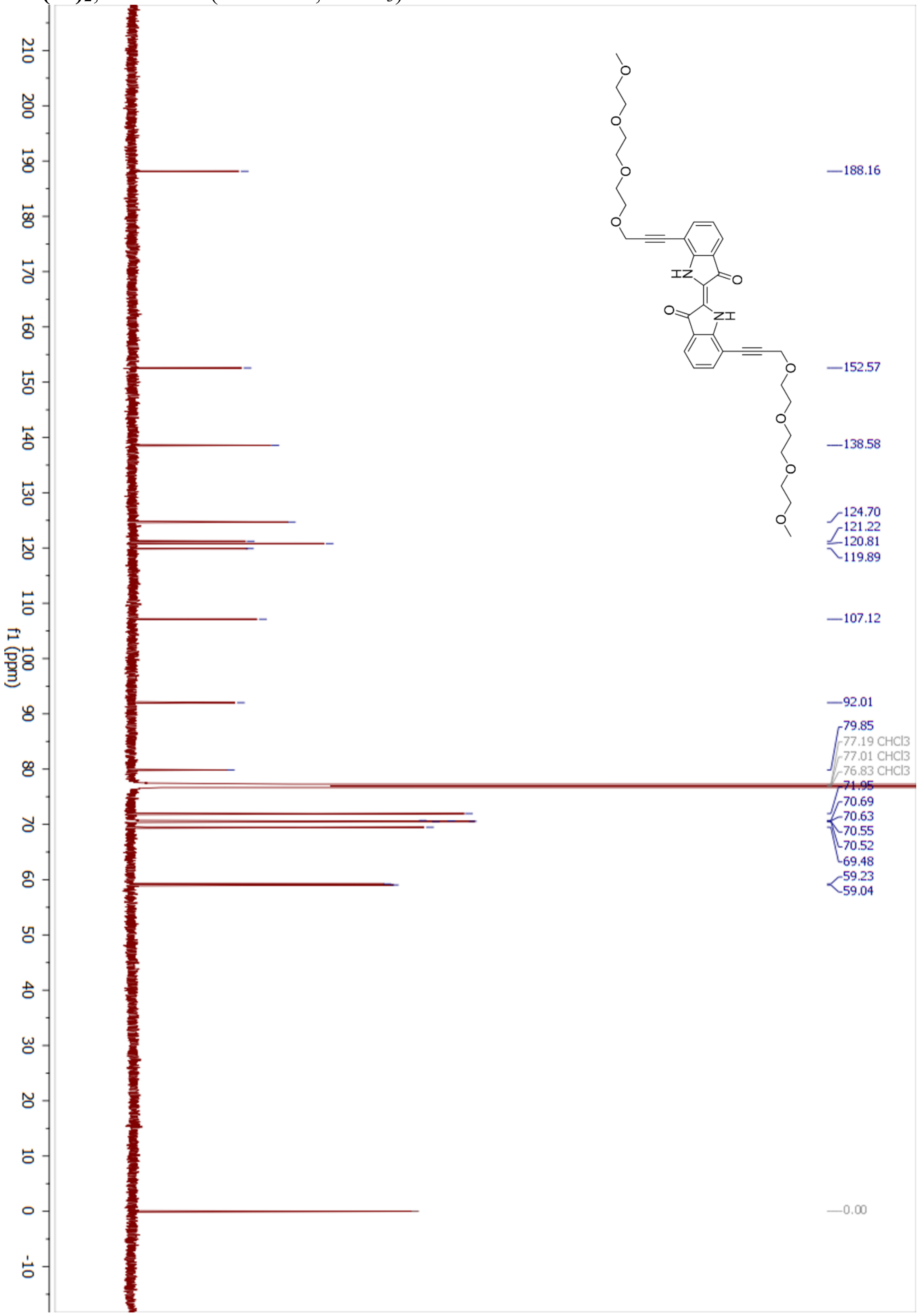

Supplement: Supplementary file 1 [file molecules-28-04143-s001.zip › molecules-2382483-supplementary.pdf]
